# Data generation and modeling during COVID-19: utility, barriers, and priorities for future investments in public health response

**DOI:** 10.3389/fpubh.2026.1718094

**Published:** 2026-02-23

**Authors:** Kristen Nixon, Shaun Truelove, Lauren Gardner

**Affiliations:** 1 Department of Civil and Systems Engineering, Johns Hopkins University, Baltimore, MD, United States; 2 Department of International Health, Johns Hopkins Bloomberg School of Public Health, Baltimore, MD, United States; 3 Department of Epidemiology, Johns Hopkins Bloomberg School of Public Health, Baltimore, MD, United States

**Keywords:** COVID-19, data, mathematical modeling, decision-making, epidemiology, infectious disease

## Abstract

During the COVID-19 pandemic, policymakers and business leaders had to rapidly make consequential decisions, and researchers rushed to provide useful information, making use of data, infectious disease models, and public health knowledge. Our study surveyed 112 individuals engaged in COVID-19 response in the US, including data collectors, modelers, and users of these tools to determine how useful different data-driven tools were for informing response work, the most impactful challenges, and the most promising opportunities for future investment. Respondents overwhelmingly found data, models, and collaborations with researchers to be useful. The most impactful challenges, and also the most promising areas for future investment, were in data availability and quality. Respondents wanted higher quality data, more granular data, and access to a variety of data types. The second most influential challenge was insufficient human resources, with public health institutions being particularly under-resourced. Academics also played a valuable role, despite incentives that often conflict with real-time response work. Our survey results also highlight the importance and challenges of translational work, including shortcomings in science communication and difficulty navigating political influences. This study provides concrete evidence of the value of data and modeling tools for epidemic response and outlines priorities for future investment.

## 1 Introduction

Researchers played a critical role in supporting COVID-19 response efforts, including informing intervention decisions, short-term forecasting, characterizing transmission and disease dynamics, and exploring potential long-term scenarios. Many researchers worked with public health institutions to define their work, share results, and directly support response efforts. In the United States, massive collaborative efforts like the US Forecast Hub and US Scenario Modeling Hub harnessed the work of many researchers to answer critical questions for decision-makers. The US Forecast Hub combined the efforts of over 90 teams to provide a publicly available, interactive visualization of predicted case, death, and hospitalization counts in the next 4 weeks (1). The US Scenario Modeling Hub partnered with public health decision-makers to provide crucial situational awareness and inform planning of interventions with long-term scenario models (2, 3). The Scenario Modeling Hub gave frequent presentations to US Federal Agencies and public health institutions, in addition to directly informing the Advisory Committee for Immunization Practices’ recommendations on multiple occasions (3).

Despite the outpouring of research effort to support COVID-19 response, concrete information on the usefulness of different projects for decision-makers is limited and is largely anecdotal. Several widely publicized stories bemoaned model-driven decision-making gone wrong (4). Other anecdotal (4, 5) and qualitative (6, 7) accounts highlight the role of data, models, and scientific evidence in decision-making. One global study interviewed 27 scientific advisors in early 2021, focusing on challenges to evidence-based decision-making (7). Another study from early 2021 targeted modelers in the UK and conducted a brief survey with 46 responses and 11 interviews (6). These studies identified diverse factors that impacted the ability to use data, modeling, and scientific evidence to drive epidemic decision-making. Many translational elements were influential, including handling and communicating uncertainty (6, 8), building trusting relationships between researchers and stakeholders (6, 8), the need for transparency (8), scientific literacy (8), and political factors (8). In addition, a lack of feedback on the utility of the researchers’ work and their limited understanding of policymaking processes provided challenges (7, 8).

Lipsitch et al. compiled the expertise of researchers and practitioners on infectious disease data surveillance via a qualitative manuscript on the insights from a symposium held in 2022 (9). This paper thoroughly outlined the data challenges during the COVID-19 pandemic and concrete steps to improve pandemic preparedness in the US. Investing in data infrastructure was of primary importance, including supporting a variety of data streams and high-quality data. To improve translation of data and modeling tools to support public health practice, they recommend stronger ties between academics and public health institutions, a workforce expansion plan for emergencies, and improving data literacy in public health institutions.

In contrast to prior, qualitative work, we provide quantitative evidence from a multiple-choice survey of US COVID-19 data collectors, modelers, and decision-makers about the use of data and models for epidemic response. We quantify how useful different data and modeling tools were for decision-makers, the impact of different challenges, and the most promising investments for improving use of data and modeling tools for public health response. The survey included 112 participants and was conducted in fall of 2023, so it captures a variety of phases of the COVID-19 pandemic, including the start of the pandemic, before and after vaccines, and variant-driven waves. This study provides strong evidence on a variety of critical questions about data-driven COVID-19 response, demonstrating that data and models were useful for informing public health decision making and identifying investment priorities for improving pandemic preparedness. These findings provide concrete evidence to justify investment in public health work and guidance on investment priorities at a time when scientific research funding and governmental capacity in public health is being dismantled in the US.

## 2 Methods

### 2.1 Survey design and distribution

We conducted a survey of public health professionals, infectious disease researchers and modelers, and health care professionals to quantify the impacts and use of data and modeling tools during COVID-19 response. The survey is mostly comprised of multiple-choice questions but also has a few open-response questions. Study participants were recruited through mass emails and solely included participants from the US due to funding restrictions. We analyzed the survey results using descriptive statistics and thematic analysis of the open response portions.

Study participants were recruited through multiple existing email listservs and networks, including listservs targeted at infectious disease researchers and modelers, public health professionals, and health care researchers and workers. The survey was conducted using Qualtrics. Participants were offered a $100 gift card for completion of the full survey using Tango. This study was reviewed and deemed exempt by the Johns Hopkins Bloomberg School of Public Health Institutional Review Board, since our survey is limited to asking about respondents’ professional experiences. To protect the privacy of our respondents, we share deidentified, aggregated data, which are included in the Supplementary data sheet 3. For privacy reasons, we do not provide data for questions with a sample size of less than 5, and we cannot make the responses to open response questions available.

### 2.2 Survey content

The survey was targeted both at those who made COVID-19 data and modeling tools and those who used them to inform decision-making. To tailor the questionnaire to each group, the survey was divided up into two main parts. Those that primarily built COVID-19 data or modeling tools were directed to the “tool builders branch” of the survey, while those that primarily used these tools were directed to the “tool users branch”. See Supplementary data sheet 1 for further information on survey content and Supplementary data sheet 2 for a complete list of survey questions.

## 3 Discussion

To prioritize readability of this manuscript for diverse stakeholders, this section contains a high-level overview of the key takeaways from the survey, whereas detailed analyses of survey responses can be found in Supplementary data sheet 1. Our survey received 112 responses, which included data collectors, modelers, and users of these tools. Of note, the sample sizes for individual questions vary substantially due to some questions only being applicable for a smaller fraction of the respondents, like those that participated in collaborations. We found that users of data and modeling tools overwhelmingly found them to be useful. The most impactful challenges of response work were in data quality and availability, followed by insufficient human resources. Translating data and modeling work to be useful for decision-makers was a major challenge, and direct collaborations between researchers and practitioners proved to be a promising avenue for more effective translation of these tools to support decision-making. Table 1 summarizes the investment priorities identified from the survey results.

**Table 1:** Summary of investment priorities based on survey results.

Identified investment priorities for epidemic preparedness and response
Priority 1. Provide higher quality, more granular, and broader types of data: Provide timely, reliable ground-truth data on cases, deaths, and hospitalizations Ensure consistency in data collection over time and across jurisdictions Minimize burden of data wrangling and harmonization Collect a variety of data types, in particular, serosurveillance, wastewater surveillance, genomic surveillance, behavioral data, and clinical data Provide more granular data, by geography and subgroups Priority 2. Build modeling capacity in critical areas for decision support: Determining effectiveness of interventions Managing resource allocation Predicting the near future Priority 3. Prioritize translational work: Facilitate collaborations between researchers and practitioners Communicate appropriate interpretations of model results to decision-makers and the public Invest in strategic, effective science communication methods Develop strategies for navigating political influences on public health Priority 4. Support a public health and academic workforce that is ready for public health emergency response: Attract and retain a skilled public health workforce Fund public health institutions, especially for the collection of critical datasets Modify academic incentive structures to reward contributions to response work

This study conducted a survey of 112 public health researchers and practitioners during the COVID-19 pandemic. Respondents in the survey were asked to identify the most impactful challenges to their response work and their top priorities for future investment, which are reflected in this summary of priorities.

### 3.1 Key finding 1: data, models, and collaborations with researchers were useful for decision-makers

Data and models are powerful tools for driving evidence-based pandemic response. When asked to rate the usefulness of data and modeling tools, users overwhelmingly found them to be useful for informing their COVID-19 response work (Q171, Q228). Direct collaborations between researchers and practitioners were particularly valuable (Q253). Our findings are in line with anecdotal evidence on the value of data-driven tools for pandemic response (2, 6) and also provide quantitative evidence to counteract some lingering distrust from some of the highly publicized negative anecdotes about uses of data-driven tools during COVID-19 (4).

### 3.2 Data

#### 3.2.1 Key finding 2: limitations in data quality and accessibility presented the most impactful challenges and are the most promising areas for future investment

Data are the foundation for effective pandemic response. Reliable, timely data are critical for situational awareness, allow us to learn from past situations, and are the foundation to more sophisticated analyses and modeling. Thus, it is unsurprising that our survey found that data quality and accessibility were the primary challenges to COVID-19 response work and that they present the most promising opportunities for future investment. For tool builders, data quality and availability were the most impactful challenges (Q152), and for tool users, data quality was first and data accessibility was third (Q263). Underlying many of the data quality and accessibility issues is the underfunded, decentralized government data infrastructure in the US. Investing to strengthen and standardize these systems, which are the bedrock for all response work, would have exponential returns for public health practice. A detailed resource on how to improve infectious disease surveillance, which identifies many of the same priorities we do using qualitative methods, is provided by Lipsitch et al. (9).

#### 3.2.2 Key finding 3: timely, reliable counts of cases, deaths, and hospitalizations were a critical foundation for response efforts

Respondents repeatedly expressed the critical nature of high-quality case, death, and hospitalization count data, which provide “ground truth” data on disease burden. These data types were rated as the most useful both by modelers and decision-makers (Q38, Q171), and they were in the top 5 investment priorities for users and top 4 for builders (Q264, Q153). These data provide the clearest measurement of the impact of an infectious disease, so data quality issues in these datasets undermine the entire enterprise of building data-driven tools for epidemic response (10). Without consistent, accurate ground truth data, models cannot be properly trained or calibrated, and the utility of more auxiliary signals, like wastewater or behavioral data, is much more difficult to evaluate.

#### 3.2.3 Key finding 4: the lack of consistency in data collection over time and across jurisdictions presented challenges

The challenges posed by inconsistent data collection were a recurring theme throughout the multiple choice and open response portions of the survey and have also been documented in prior work (10–12). Lack of standardization in data definitions was a top 5 challenge for a quarter of tool builders and was rated as an impactful challenge for data collectors and managers (Q152, Q80, Q165). For example, the lack of a standard definition for a COVID-19 case or death across US states posed a substantial barrier to efforts to establish consistent metrics for comparison or apply any information learned from one state to another. Changes in the types of tests available throughout the COVID-19 pandemic contributed to the discrepancy in case definitions, in addition to complicating the interpretation of valuable test positivity metrics. Inconsistent definitions of demographic characteristics hampered our ability to study inequities and identify groups that are most at-risk. For data to be a meaningful signal, it must be collected consistently and in a uniform manner.

#### 3.2.4 Key finding 5: data wrangling and harmonization were a time drain

Accessing, processing, and harmonizing data sources can be incredibly time-consuming. These issues were mentioned in open response portions of our survey and have also been discussed in prior work (11, 12). Analyses commonly make use of several data sources, and the time spent harmonizing these datasets is a significant detraction from work output. Many of the data issues previously discussed combine to compound the burden of data wrangling, including handling inconsistent data collection, miscellaneous data quality issues, and the need to incorporate several data sources to try and compensate for the limitations of each source (Q80). Data centralization efforts, like the JHU CSSE Dashboard (13) and the COVID-19 Delphi Epidata API (14), were very helpful in decreasing the burden of data wrangling on end users.

#### 3.2.5 Key finding 6: there was a demand for a variety of data types, in particular, serosurveillance, wastewater surveillance, genomic surveillance, behavioral data, and clinical data

Modelers and tool users found a variety of data types to be useful. All but one of the 16 data types asked about in the survey were rated as moderately useful or better (Q171), and when selecting top priorities for investment, every data source was chosen by at least 10% of tool users, with several being chosen by more than a third of respondents (Q264). Every data source, each with its own biases and limitations, contributes a unique piece to form a complete picture of the situation. Therefore, having access to a variety of different data sources is critical for response efforts (9, 10). Especially in the context of the disinvestment in reporting of cases, deaths, and hospitalizations data, alternative surveillance data are even more important. Some datasets that were seen as particularly promising by our respondents were serosurveillance, to get a more reliable estimate of the proportion of the population that has been infected and circumvent case reporting biases, wastewater surveillance, to provide an early warning signal of an outbreak, genomic surveillance, for a warning on new variants, behavioral data, to capture risk mitigation behaviors that may affect transmission, and individual health records, to directly quantify factors that influence health outcomes (Q264).

#### 3.2.6 Key finding 7: the lack of granular data, by geography and subgroups, was a barrier to the targeted analyses that are most useful for supporting local decision-makers

Across all data types, more than a third of tool users cited limited data at the geographic resolution of interest as a challenge and about a third selected limited data on subgroups of interest (Q172-Q187). In open response sections, multiple respondents noted that a lack of reliable data at a high geographic resolution was a barrier to providing analysis to support local decision-makers, whose questions were highly location-dependent. A 2024 report from the Council of State and Territorial Epidemiologists had a similar finding, specifically that 43% of US states and territories indicated that models were not relevant to or representative of their jurisdiction (15). We also need data broken down by subgroups of interest, like race and age, in order to support targeted, equitable epidemic response. Limited specificity in data in turn limits the depth of targeted questions that researchers and practitioners can answer, and more targeted insights are often the most useful and actionable.

### 3.3 Modeling

#### 3.3.1 Key finding 8: models are a powerful tool, but were limited by data challenges and require translational effort

Modeling is a powerful tool for understanding epidemics and informing response efforts. Built on a foundation of data, models can provide more sophisticated insights on questions like the effectiveness of interventions, optimal management of resources, and near future predictions, which were the 1st, 3rd, and 4th highest priorities for future investment from tool users (Q264). Additionally, respondents rated all five model types we ask about, nowcasting, forecasting, projections, optimization, and retrospective analysis, to be very useful (Q228).

According to modelers, one of the biggest challenges to their work was data issues (Q58). In the words of one respondent: “Good data is really key—we spent a lot of time trying to figure out how to make do with insufficient data”. Since all models are built on data to some extent, data issues are often the limiting factor of modeling efforts. Without reliable data, it is very difficult for models to produce accurate predictions or insights, and the interpretation of modeling results becomes more complicated. Data priorities that are particularly relevant for modelers include having reliable data on disease burden, consistent data definitions, access to a variety of alternative surveillance data, timeliness, and granularity by geography and subgroups (Q44). In addition to data issues, the uncertain nature of the pandemic presented serious challenges to modeling work (Q58). The rapidly evolving situation, due to factors like variants and vaccination, made it difficult to rely on past data to provide insight on the future.

In this hectic environment, thoughtful communication of modeling results was paramount. However, the combination of many models being publicly available, often with divergent predictions, limited model literacy, and a lack of clear communication about the assumptions and appropriate interpretations of these models, led to a loss of trust in modeling when predictions were perceived to be wrong (4).

### 3.4 Translating data and modeling tools for decision-making

#### 3.4.1 Key finding 9: collaborations between researchers and practitioners were valuable and impacted decision-making

Collaborations between researchers and practitioners are a powerful way to promote successful translation of data and modeling tools to support decision makers and prevent misunderstanding. Within trusting partnerships, stakeholders had the opportunity to glean the insights that come from a more nuanced understanding of models, the underlying data, their assumptions, and results, while researchers could develop a better understanding of the needs of practitioners. In our survey, 70% of tool users said that collaborations with researchers informed their situational awareness, and more than half said it directly impacted their decision-making (Q253). However, only a third of tool builders that collaborated with stakeholders got feedback on if and how their work was useful for informing response (Q124). It is critical that tool builders get constructive feedback on the utility of their work, so that they can direct their efforts toward what is useful for decision-makers. Beyond just communicating whether the tools provided were useful or not, elucidating the nuances of what makes a tool useful is extremely valuable, albeit a difficult question to answer.

An illustrative example of an effort that centered building relationships between researchers and stakeholders is the US Scenario Modeling Hub (SMH). The SMH worked with decision-makers to define the scenarios to model and actively participated in interpreting and communicating results for decision-makers (3). The SMH coordinated the efforts of several modeling teams, so the results would benefit from the combined insights of different modeling approaches and better communicate uncertainty, while avoiding the problem of overwhelming decision-makers with too many models with varying assumptions and purposes. Feedback from our respondents on the utility of the SMH was very positive, with users citing it as useful for strategic planning, understanding uncertainty, and situational awareness. One respondent noted that just discussing why certain scenarios were chosen provided useful situational awareness.

#### 3.4.2 Key finding 10: struggles with science communication and political influence hindered effective translation of data and models for use by decision-makers

The practice of communicating data and modeling results to audiences with limited data and model literacy presented many challenges and lessons during COVID-19 response (10). Our survey respondents struggled to communicate the goals, capabilities, and appropriate interpretations of these tools in a rapidly evolving environment. In the words of one respondent, some decision-makers felt that they could “only make decisions based on data, not speculation.” Clear communication of the limitations of data sources, assumptions behind modeling, uncertainties, and the types of insights provided by models could help dispel the false dichotomy between raw data and more sophisticated, “speculative” analyses and the misconception that inaccurate future predictions is an indication that models in general aren’t useful.

The influence of politics further complicated an already complex exchange between tool users and builders. In our survey, one-fourth of tool users thought that politics mainly dictated decision-making (Q258). While data and modeling tools can be incredibly useful, appropriate interpretation is often nuanced and not conducive to the straightforward answers some politicians might expect from data. In an extremely polarized environment, the lack of data and model literacy among the public enabled politicians to wield these tools to provide credence to their policies when convenient and ignore them when they did not support their agenda. To realize the full benefits of available data and modeling tools, we need to invest in science literacy among both the public and decision-makers. In addition, supporting public health work should be a non-controversial, bipartisan priority, but unfortunately, immense repairs are needed to depoliticize public health after the polarization that occurred during the COVID-19 pandemic.

### 3.5 Human and financial resources

#### 3.5.1 Key finding 11: insufficient human and financial resources were a consequential challenge, second only to data issues

To prepare an effective epidemic response system, we need broad investment in the field of public health, from sustaining a skilled workforce to providing funding for research projects and critical institutions. In our survey, insufficient human resources was the second highest overall challenge for tool builders and the third highest for tool users, being secondary only to data issues (Q152, Q263). Investment is essential for attracting, training, and retaining employees, particularly individuals with data and modeling skills, to build a strong public health workforce (9).

Funding presented obstacles to COVID-19 response work for builders, but groups largely overcame these challenges by reallocating funding from other projects or working on a project despite having no funding (Q152, Q21). Although some funding was available during the peak phases of COVID-19, investment in pandemic preparedness quickly dwindled as COVID-19 became a less pressing concern for the public. To respond effectively to epidemics, we need consistent investment in the necessary human resources and infrastructure during peacetime, not just when society is in crisis.

### 3.6 Critical roles of different stakeholders

#### 3.6.1 Key finding 12: public health institutions played a critical role in pandemic response but were especially under-resourced

Public health institutions have a vital role in epidemic response; they manage essential data sources, communicate information to the public, and work to combat health inequities (Q164, Q158). However, we found that this group was even more limited by a lack of human resources with the necessary expertise than other respondents (Q263, Q191, Q230). Similarly, a 2024 report from the Council of State and Territorial Epidemiologists found that the inability to hire a workforce with necessary skills was a top challenge to data modernization efforts (15). Public health institutions provide many of the critical datasets for response, from foundational cases, deaths, and hospitalizations data, to alternative forms of surveillance, like genomic and wastewater data (Q164). Although external researchers can provide valuable contributions to public health institutions, internal capacity is needed to perform functions like managing sensitive data and completing routine analyses. In order to reliably perform these vital functions, we need to fund data infrastructure and support a skilled workforce at public health institutions. Unfortunately, given the current political landscape in the US, where there have been concerted efforts to defund public health work, it is risky to rely on continued political support to establish critical response systems that need consistent funding to remain functional and ready for an emergency.

#### 3.6.2 Key finding 13: academics made valuable contributions, despite the conflicting nature of academic incentives and the demands of emergency response

Academics played a critical role in pandemic response by addressing big picture research objectives that were overshadowed by more pressing concerns, like resource allocation and near-future predictions, for many response organizations. Academics were able to focus on longer-term objectives like the effectiveness of different interventions (the top priority for investment for both builders and users), learning about factors that impact transmission (among the top priorities), and long-term scenario modeling (Q23). Academics were also more likely to model subgroups, like medical facilities or schools, and they advanced the state-of-the art use of alternative surveillance data, like genomic, serological, and wastewater data (Q36, Q37).

Many academics prioritized real-time response work during the COVID-19 pandemic, at the expense of other research endeavors with a clearer path to publication. Nearly a third of academics did not publish in a peer-reviewed journal nor post a preprint by the time of our survey in fall of 2023, and a majority communicated directly with stakeholders, many of which were public health institutions (Q91, Q108). Incentives in academia prize publications and citations, which are particularly critical for junior faculty that are in fierce competition to secure a tenured position. If we want academics to continue to play a significant role in real-time response work, we need to reward faculty that pursue these efforts and consider delegating the more routine response work to organizations like the CDC Center for Forecasting and Analytics (9, 10, 16).

### 3.7 Limitations

COVID-19 response efforts existed in a complex system where decisions were made based on a variety of inputs, which makes it difficult to isolate and measure the specific impact of data and modeling tools. Our approach directly asks users of these tools about their perception of the utility of these tools, which introduces subjectivity but also offers the opportunity to disentangle the specific role of data and models in response. The timing of the survey (fall 2023) allowed us to capture a variety of phases of the COVID-19 pandemic, but the significant passage of time since the early, acute phases of the pandemic may mean that respondents’ memories of this time are less clear.

While we attempted to reach any individuals that engaged in building or using data and modeling tools for COVID-19 response, it is likely that our respondents are biased toward those who are already connected to the academic modeling community due to the networks we leveraged for outreach. While the participants were a majority modelers (*n* = 63) and university-affiliates (*n* = 69), there were still sufficient numbers of respondents who were users of data and modeling tools (*n* = 30). Among the 30 tool users, 15 were also involved in developing internal models, which could color their opinion on the utility of modeling. However, due to their direct involvement in using tools to conduct response efforts, their opinion on the utility of models for this purpose is still informative. Notably, sample sizes vary substantially throughout the survey due to some questions only being applicable for a smaller fraction of the respondents, like those that participated in collaborations. Questions with sample sizes ranging from 10 to 112 were used to support our takeaways, with the subanalyses on public health institutions and data collectors being the only ones with a sample size of less than 20. Sample sizes for all questions are available in the Supplementary data sheet 3.

We rigorously report the results of the survey using descriptive statistics in Supplementary data sheet 1. Hypothesis testing was not feasible for our analysis due to the large size of the survey (>200 questions) with several different question and data types and substantial variation in sample sizes and distributions. The takeaways were supported by a straightforward, transparent reporting of the question results and are based on multiple pieces of evidence, including multiple choice questions and open response statements. Our findings are also consistent with prior work and expert knowledge on COVID-19 pandemic response.

In support of our goal to collect quantitative data, we aimed to capture as much information as possible via multiple choice questions, and in doing so, many questions only allowed predefined, categorical responses. While we carefully designed the questions and response options to capture all relevant options and piloted our survey with colleagues in academia and public health practitioners, we inevitably missed opportunities to capture some information. To compensate for this, we offered ‘Other, please specify’ options throughout the survey in addition to open response questions, but this cannot completely compensate for omitting an answer choice, for example, failing to list a challenge that some respondents found to be impactful.

Although policymakers were important decision-makers in COVID-19 response, our survey did not target them because we wanted to get information from experts in infectious disease response, like researchers and practitioners. These individuals did the on-the-ground implementation work and were often the critical providers of information to policymakers. We indirectly capture influence on policymakers by asking respondents about their collaborations with and influence on decision-makers, including policymakers.

## 4 Conclusion

Over the course of the COVID-19 pandemic, researchers, modelers, and public health professionals dedicated enormous amounts of effort and time to collecting, curating, and applying COVID-19 data and modeling tools. These efforts had immense effects on our lives, either through awareness of the evolving pandemic, or through the impact of actions taken that were evidenced by these efforts. While we cannot fully quantify the extent or the impacts of these efforts, we can assess instances of success or failure, challenges, the value they provided, and the gaps that existed and may still remain.

Our study offers strong evidence that COVID-19 data and modeling tools were useful for informing response efforts, and we identify the most promising areas to invest in for future preparedness, including data availability and quality, model capabilities, translational work, and workforce development. In the current political environment in the US, in which public health research and practice is under attack from the federal government, concrete evidence of the value of this work is an important contribution.

## Data Availability

The original contributions presented in the study are included in the article/Supplementary material, further inquiries can be directed to the corresponding author.

## Supplementary material

SUPPLEMENTARY Data Sheet 1 Supplementary Material A. This file contains additional information about the content and results of the survey.

SUPPLEMENTARY Data Sheet 2 Supplementary Material B: Survey Questions. This file contains a complete record of survey questions, answer choices, and logic.

SUPPLEMENTARY Data Sheet 3 Aggregated Survey Data. This JSON file contains aggregate survey response data.
