# Supplementary material for: Data generation and modeling during COVID-19: utility, barriers, and priorities for future investments in public health response
Source: Front Public Health. 2026 Feb 23;14:1718094. doi: 10.3389/fpubh.2026.1718094 (PMC12968176; doi:10.3389/fpubh.2026.1718094)
Supplement: SUPPLEMENTARY Data Sheet 1 — Supplementary Material A. This file contains additional information about the content and results of the survey. [file Data_Sheet_1.pdf]

Supplementary Material A

1. Survey Content 2

1.1. Tool Builders Branch 4

1.2. Tool Users Branch 4

2. Extended Results 6

2.1. Sample Characteristics 6

2.2. Data, Models, and Collaborations with Researchers were useful for pandemic response 7

2.3. Data 8

2.3.1. Data quality 9
2.3.2. Data accessibility 9
2.3.3. Challenges by data type 10
2.3.4. Challenges for dataset builders and managers 12

2.4. Modeling 13

2.5. Translation 15

2.5.1. Top challenges to translational work 15
2.5.2. Collaborations between researchers and decision-makers 15
2.5.3. Data and modeling tools' impact on decision-making 18
2.5.4. Lack of feedback for tool builders 18
2.5.5. The US Forecast and Scenario Hub 19
2.5.6. Science communication 19
2.5.7. Impact of politics 19

2.6. Human and Financial Resources 20

2.7. Top Priorities for Investment 20

2.8. Subgroup Analyses 21

2.8.1. Public Health Institutions 21
2.8.2. Academia 22

1. Survey Content

The following question (Q13) was used to determine whether to send users to the builders or users branch of the survey.

"What was your primary role in COVID-19 surveillance and response efforts?

If you work in academia, your default response would likely be "building" unless your role included making decisions about response, such as determining university COVID-19 response policy.

If you work in a practice or response focused organization (public health institution, healthcare institution, etc.), your default response will likely be either "using" or "both". An exception would be if your role was primarily focused on building models or datasets, and you were not making decisions or directing response efforts based on these tools.

- *Building models, tools, and/or data sets to support COVID-19 surveillance and response efforts, including adapting existing methods to inform COVID-19 response (usually academics, modelers, data collectors). This includes if you were developing tools for use by decision-makers*
- *Using models, tools, and/or data sets to make decisions or direct response efforts (usually public health practitioners, policymakers)*
- *I had significant involvement in both building models, tools, and/or data sets AND using them to make decisions or direct response efforts."*

The distinction between builders and users was blurry in practice, so we provided an option for users who felt that they had significant involvement both in building and using COVID-19 tools. Respondents that selected "both" were sent to the user branch of the survey, since each branch the survey was too long (about 30 minutes) to ask "both" respondents to fill out both the build and use branch of the survey, and we wanted to prioritize hearing about uses of these tools. Within the user branch, there was a heavily abbreviated version of the questions in the building branch for those that also were involved in building datasets or models.

When designing this survey, we attempted to capture as much information as possible in multiple choice questions, but we also offered an "Other, please specify" choice with a text input option whenever relevant. To gauge the relative impact of different challenges, for these questions we provide a list of challenges and ask respondents to rate how impactful each challenge was on a scale from 1 to 5 (not impactful, slightly impactful, moderately impactful, impactful, or strongly impactful), with an option to enter in other challenges. In addition, after each section of the survey, we have an open response question asking if there's anything else they would like to share.

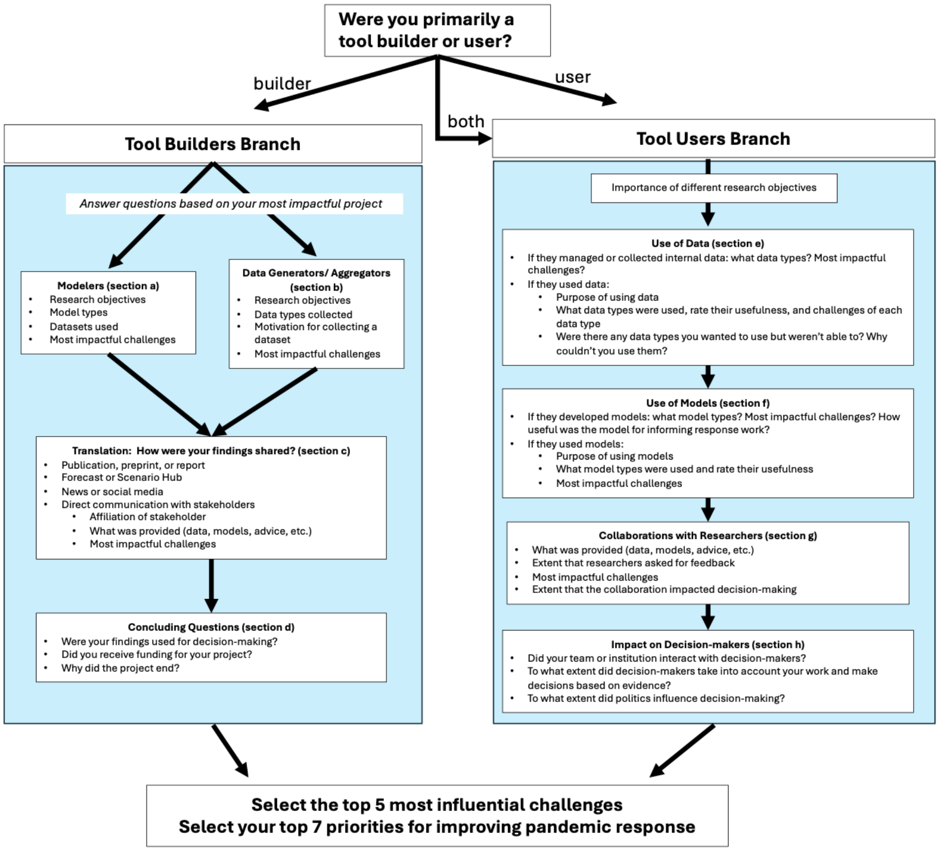

Figure 1: Schematic of survey question logic and content.

Respondents that selected building COVID-19 data or modeling tools as their primary role were asked to select their most impactful project to report on. Here are the instructions shown to respondents:

"For the remainder of the survey, you will answer questions based on one selected project. We recommend you select the most impactful project that you led.

In this survey we are primarily interested in "impact" on decision-makers (e.g. policymakers, public health institutions, healthcare institutions), and secondarily on academic impact (e.g. publications, citations). "

These respondents were then asked to indicate whether their selected project was modeling, data generation (producing data that were not previously available, like sequencing to produce genomic data), or data aggregation (collecting and centralizing existing data for ease of access, like the JHU CSSE dashboard, Q17). Respondents then answer a series of questions about the purpose, methods, challenges, and how they shared their work.

Respondents who selected either using tools or both building and using tools as their primary role were sent to the "tool user" branch of the survey. Within this branch, respondents were asked about their use of data or modeling tools and their collaborations with researchers, and whether these resources were useful.

Both tool builders and users conclude the survey by selecting the top 5 most impactful challenges to COVID-19 response work and the 7 highest priorities for future investment out of the data types and research objectives covered in this survey (Q152, Q153, Q263, Q264). On the builder side of the survey, there were more answer choices on the specific type of data accessibility and data quality challenges (Q152), whereas the users had more general answer choices (Q263).

1.1. **Tool Builders Branch**

The respondents that selected that their primary role was building COVID-19 data or modeling tools are then asked to indicate whether their selected project was modeling, data generation (producing data that were not previously available, like sequencing to produce genomic data), or data aggregation (collecting and centralizing existing data for ease of access, like the JHU CSSE dashboard, Q17).

Modelers, section a. objectives (Q23), modeling types (retrospective analysis, nowcasting, forecasting, projections, or optimization, Q24), datasets used and their utility (Q37, Q38), and the most impactful challenges (Q44, Q58).

Data Generators/Aggregators, section b. Respondents that select either data generation or aggregation are asked an analogous set of questions: research objectives (Q64), data types collected (Q62), motivation for collecting a dataset (Q63), and most impactful challenges (Q79, Q80, Q81).

Translation, section c. All tool builders were asked a series of questions about how their work was shared, whether by peer-reviewed publication, preprint, by news or social media, via the Forecast or Scenario Modeling Hub, or by direct communication with stakeholders (Q91). Those that selected direct communication with stakeholders were asked about the institutional affiliation of the stakeholder (public health institutions, other government officials, healthcare institutions, or other, Q108), what was provided to the stakeholders (data, models, advice, etc., Q114), and most impactful challenges (Q120).

Concluding Questions, section d. Tool builders were also asked if their findings were used to inform decision-making (Q147, Q148), whether they were funded (Q21, Q22), and why the project ended (Q151).

1.2. **Tool Users Branch**

Respondents that selected that their primary role was either using tools or both building and using tools are sent to the using tools branch of the survey. To account for respondents that indicated that their role included both building and using, respondents are asked if they managed datasets or built models, and if so, they answer a few basic questions about these efforts. Within

the tool user branch, respondents are asked about their use of data or modeling tools and their collaborations with researchers, and whether these resources were useful.

Tool users are asked to rate the importance of a set of research objectives during two time periods, early in the pandemic (around January to April 2020) and the middle or control phase (around late 2020 to mid-2021, when non-pharmaceutical interventions and vaccinations were widely used to control disease spread, Q158, Q159).

Use of Data, section e. Next, tool users were asked if they managed data collected or aggregated within their institution and if they used data to inform response efforts (Q163). If they indicated that they managed internal data, they were asked what data types they managed and what the most impactful challenges to this work were (Q164, Q165). If they indicated that they used data to inform response, we ask them what data types they used (Q166), the purpose of using data (Q169), to rate the usefulness of each data type (Q171), and the challenges of using each data type (Q172-Q187). Then, we ask if there were any data types they wanted to use but were not able to (Q192). For each of the selected choices, we ask why they weren't able to use this data type (Q193-Q208).

Use of Models, section f. Tool users are then asked if they developed or used models for COVID-19 response (Q213, Q217). If they developed models, we asked about the research objectives (Q218), model types (Q219), challenges (Q222), and how useful the models were for informing response work (Q225). If they used models, we asked about the purpose of using models (Q226), what model types they used (Q227), how useful each type was Q228), and the most impactful challenges (Q230).

Collaborations with Researchers, section g. We then ask tool users if they collaborated with researchers (Q234). If they did, we ask what the researchers provided (Q243), the extent to which researchers asked for feedback (Q246, Q248), the most impactful challenges (Q249), and the extent to which the collaboration impacted decision-making (Q253).

Impact on decision-makers, section h. To account for situations in which respondents in the tool user branch may not be the final decision-maker (e.g. a public health institution employee whose institutional leadership interacts with policymakers), we ask whether they interacted with relevant decision-makers and if the leadership of their institution did (Q257). We then ask respondents to rate the extent to which they agree with the following statements: decision-makers took into account our work; decision-makers made decisions based on data, research, and/or public health knowledge where possible; and politics mainly dictated decision-making (Q258).

A list of all survey questions can be found in Supplementary Material B.

2. Extended Results

2.1. Sample Characteristics

The survey had 112 respondents, and the average time taken to complete the survey was 24 minutes, if we assume that respondents with a duration of longer than an hour likely did not complete the survey in one sitting.

We provided a $100 incentive for completing the survey, however, some governmental organizations, including some public health institutions (PHIs), do not allow employees to accept gifts. We had 17 respondents from PHIs, 59% of which were able to accept funding, compared to 90% of other respondents (Q3). Upon inspecting the affiliations of the PHI respondents, there does not appear to be any geographical bias introduced from only some PHIs being able to accept incentives.

We received 26 respondents from the CSTE forecasting listserv (23%), 20 respondents from the MIDAS CDC listserv (18%), 16 from CSTE general listserv (14%), 15 from SPHERES (13%), 13 from the Scenario Modeling Hub (11%), 7 via contacts at Johns Hopkins Medicine (6%), 4 from the Forecast Hub (4%), 4 from the JHU Applied Modeling for Public Health Workshop (4%), and 3 or less from JHU Infectious Disease Dynamics and the INFORMS healthcare online forum. Notably, 13 out of our 17 PHI affiliated respondents came from the CSTE forecasting listserv. Additionally, 4 respondents affiliated with for-profit companies came from the SPHERES listserv, which is more than half of our 7 total respondents with this affiliation. University affiliated respondents came from several listservs, with 16 coming from MIDAS CDC, 12 from CSTE general, 11 from CSTE forecasting, 9 from the Scenario Modeling Hub, 7 from SPHERES, and at least 1 respondent from nearly every other listserv.

The survey had 82 respondents that selected building tools (73%), 8 that selected using tools (7%), and 22 (20%) that selected both (Q13). Among the 30 respondents in the tool users branch, 15 were involved in developing internal models (Q217). In terms of institutional affiliation, most of our respondents were affiliated with universities (69, 62%), followed by public health institutions (17, 15%), other for-profit companies (9, 8%), other nonprofit companies (6, 5%), healthcare institution (5, 4%) other government (5, 4%), other (3, 3%), and CDC (2, 2%, Q7). Within PHIs, 69% are from state PHIs and 31% are from county level PHIs (manual analysis from Q6—name of institution). Figure 2 visualizes the percent of each branch (build, use, or both) that is comprised of each affiliation. University affiliated respondents make up most of the building respondents, and about a fourth of both and using respondents. PHIs were the majority of the using branch, the relative majority of the both branch, and had a few respondents in the building branch. The both branch is primarily comprised of universities (24%), PHIs (38%), and other for-profit companies (14%). The using branch is 50% PHIs, 25% universities, 13% healthcare institutions, and 13% CDC.

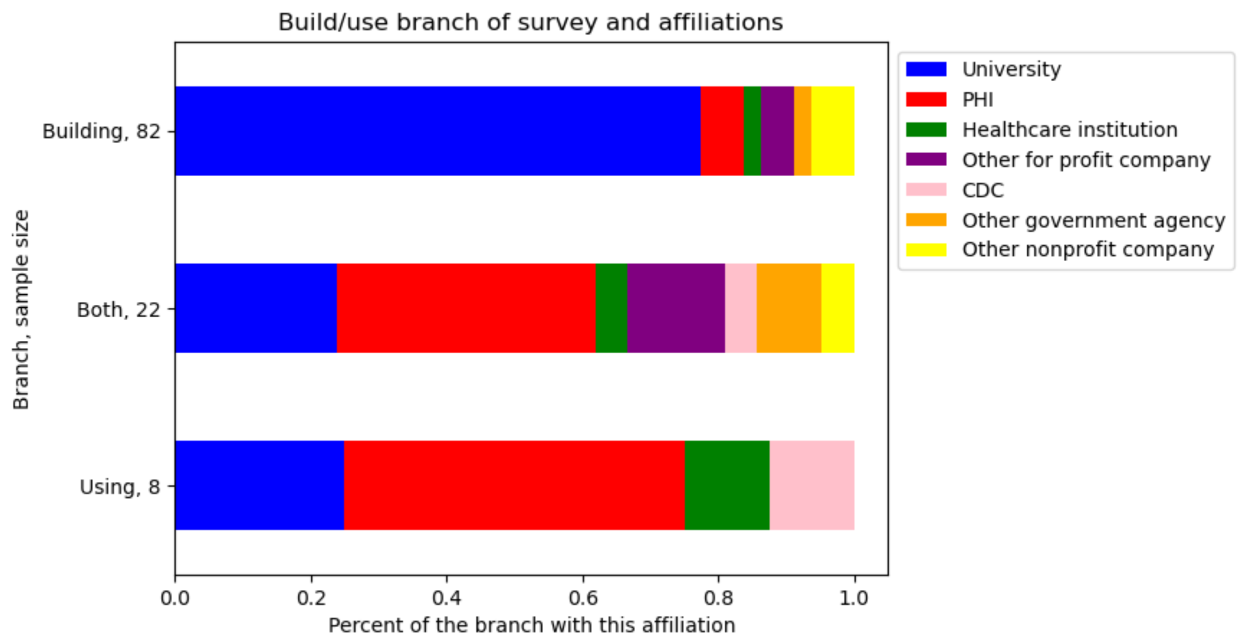

Figure 2: Breakdown of affiliations and survey branch selected

Within the building branch of the survey, 63 respondents reported on a modeling project (77%), 11 reported on a data aggregation project (13%), and 8 reported on a data generation project (10%, Q17).

The sample of our survey was skewed towards builders, university affiliates, and modelers. However, we still obtained a decent sample size for other groups of interest, including 30 respondents on the both or using branch of the survey, 17 PHI affiliates, and 19 builders that reported on a data collection project. In addition, due to the listservs we used for outreach, the sample of tool users is likely to be biased towards those that are already somewhat connected with the academic modeling community.

2.2. Data, Models, and Collaborations with Researchers were useful for pandemic response

The survey results overwhelmingly support the conclusion that data, models, and collaborations with researchers provided value. 97% of tool users used COVID-19 cases or deaths data, 90% used COVID-19 hospitalizations data, and 90% used COVID-19 testing data (Q166). When asked to rate the usefulness of each data type on a scale of 1 to 5 (not at all useful, slightly useful, moderately useful, very useful, extremely useful), the median rating for COVID-19 cases, deaths, and hospitalization was extremely useful (Q171, Figure 3). Out of all 16 data types covered in this survey, only one had a median rating below moderately useful. See Figure 3 for the sample sizes for this question, since it varies by data type.

For models, 77% of tool users used models (Q213), and all four model types (nowcasting, forecasting, projections, optimization, retrospective analysis) had a median rating of 4/very useful (Q228, Figure 5). See Figure 5 for the sample sizes for this question, since it varies by model type.

For collaborations, 77% of tool users collaborated with researchers (Q234). 70% of tool users said that what they learned from collaborations informed their situational awareness and 57% said it directly impacted their decision-making (Q253, sample size 23).

2.3. **Data**

A wide variety of data types were useful for tool users. The median rating for the usefulness of each data type was 5/extremely useful for hospitalizations and case or death data, 4.5 for vaccines allocated or administered data, 4/very useful for genomic surveillance data, EMR or individual-level health data, testing data, other demographic data, and health risk factors data, 3.5 for consumer data and COVID-19 response policy data, 3/moderately useful for other epidemiological data, serological surveillance data, mobility data, and wastewater surveillance data, and 2.5 for other behavioral data (Q171, Figure 3). Tool users' purposes of using data were primarily for situational awareness (97%), resource management (86%), and to learn about the near future (79%, Q169).

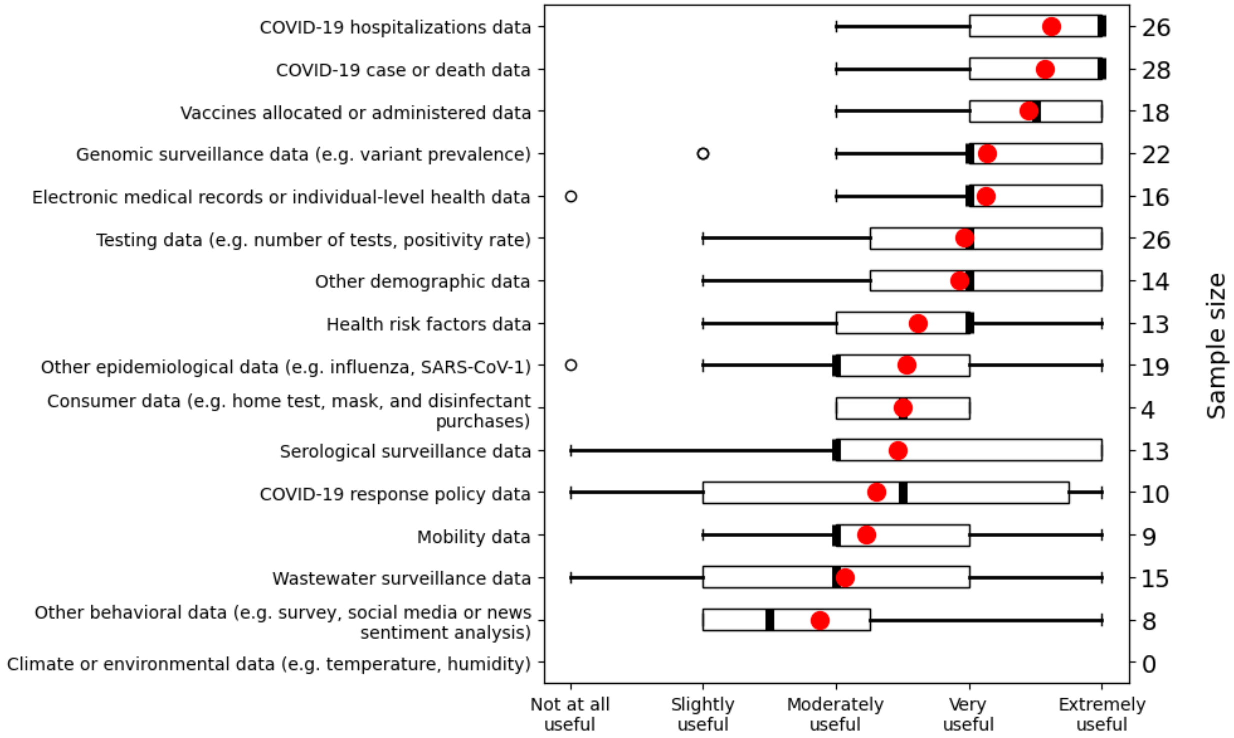

Figure 3. Plot showing the distributions of responses for Q171, usefulness of different data types for tool users. The red dots show the average value, which the y-axis values are sorted by. The sample sizes vary by choice because respondents were only shown data types that they used, as indicated in a prior question.

2.3.1. Data quality

In addition to being an incredibly useful resource, data provided some of the biggest challenges and opportunities for future investment. Data quality and accessibility issues were the most influential challenges for tool builders and users. When asked to select the most impactful 5 challenges to COVID-19 response work, the data quality challenges were the most frequently selected by both builders and users (80% users, 79% builders, Q263, Q152). Specifically, 63% of users selected data quality issues (rank 1) and 63% selected data are not timely (rank 1). Among builders, 45% selected data are not timely (rank 3), 45% selected other data quality issues (rank 4), 24% selected lack of standardization in data definitions (rank 10), and 22% selected sampling bias (rank 11, Q152).

Comments on data quality were frequent in the open response portion of the survey. Issues with timeliness, consistency and fidelity of reporting, and consistent data definitions across different jurisdictions were raised. Ease of accessing and processing data was also a major challenge, including maintaining timestamped data versions, handling data source and structure changes, harmonizing multiple data sources on a short timeframe, legal restrictions on data sharing and reuse, the difficulty of scraping data from dashboards, and working with inefficient, difficult to use data infrastructure systems.

2.3.2. Data accessibility

Data accessibility was also a top challenge for builders and users. When combining all the data accessibility options in the overall challenges questions, these challenges were the third most frequently selected for users (47%, Q263). For builders, data accessibility was essentially equivalent to the most frequently chosen option, with data accessibility at 78% and data quality at 79% (Q152). Notably, the most frequently selected challenge for tool builders was desired data were never collected (50%). The next most influential data accessibility challenges for builders were not publicly available (24%, rank 9) and privacy or data use agreement issues (18%, rank 12, Q152). For tool users, 33% selected desired data were never collected (rank 5) and 30% selected other data accessibility issues (rank 6, Q263).

In the open response questions, respondents mentioned wanting more access to certain data types, in addition to highlighting the need for more granular data. Respondents said that consistent, high-resolution serosurveillance data are critical information for determining the utility of other covariates in estimating disease burden. They also shared that data on behaviors like mask wearing, isolating when sick, and social contacts would have been helpful, although these data are difficult to collect and evaluate. Multiple respondents noted that a lack of reliable data at a high resolution was a barrier to providing analysis to support local decision-makers, whose questions were highly location-dependent. Respondents also highlighted the value of individual-level data. For

example, determining vaccine effectiveness for different variants is critical information and would be fairly straightforward to answer with individual-level health data, but with the aggregate data that were available, this analysis is much more difficult, less timely, and less reliable.

2.3.3. Challenges by data type

Figure 4 shows a heatmap of how frequently tool users selected different challenges as impactful for each data type. The most significant challenges overall were timeliness and reliability of the data, which were selected by 49% and 44% of respondents, respectively (Q172-Q187). These challenges were particularly influential for cases/deaths (89% timeliness, 61% reliability, Q172), hospitalizations (69%, 54%, Q173), and testing data (58%, 69%, Q176). Limited data that focused on geographic resolutions (38%) and subgroups of interest (31%) were also frequently chosen across all data types (Q172-Q187). Limited data at geographic resolution of interest were especially influential for wastewater surveillance (73%, Q180), cases/deaths (57%, Q172), and genomic surveillance data (50%, Q179). Lack of expertise to interpret or use data was a challenge for wastewater surveillance data (47%, Q180), mobility data (44%, Q181), and electronic medical records or individual-level health data (25%, Q178). Challenges related to privacy or data use agreements were present for electronic medical records or individual-level health data (38%, Q178), mobility data (33%, Q181), and hospitalizations data (23%, Q173).

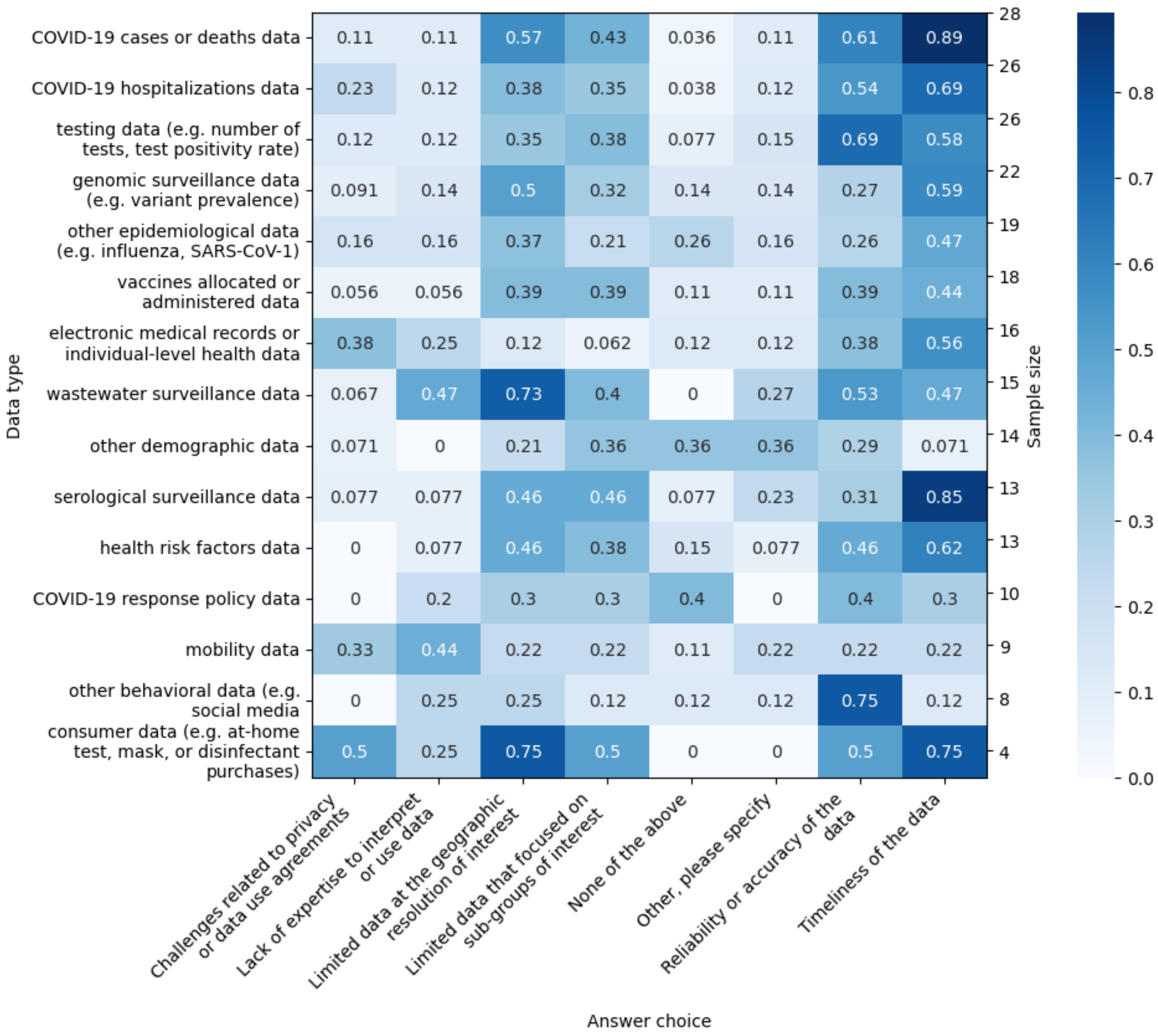

Figure 4. Heatmap of challenges selected for each data type.

In the open response portion, respondents expanded on specific challenges for some data types. For cases and deaths data, respondents noted that consistent testing and reporting practices across states would have significantly improved the quality of the data. Testing data had the additional complication of changing interpretation over time due to test availability, criteria, and types. For genomic surveillance data, respondents said that the lack of consistency in sample collection and data reporting, sampling bias, and low sample size were significant challenges. The lack of consistency in demographic categories across datasets also presented a challenge.

When asked if there were any data types that respondents wanted to use but were not able to, the top choices were EMR or individual-level health data (23%), consumer data (23%), and other behavioral data (20%, Q192). The most common barriers to using EMR

or individual-level health data were challenges related to privacy or data use agreements (71%), data were not available when I needed it (43%), and did not know how to access these data (29%, Q199). For consumer data, the most common barriers were did not know how to access the data (71%), no time to analyze or interpret these data (43%), and not available for the geographic resolution needed (29%, Q206). For other behavioral data, the top barriers were no time to analyze or interpret the data (50%) and data were not available when I needed it (50%, Q203).

2.3.4. Challenges for dataset builders and managers

Within the builder branch of the survey, 19 respondents aggregated or generated datasets. These respondents worked with every data type we asked about, except for climate data (Q62). The most frequent motivations for generating a new dataset were that the data were not available elsewhere (68%), existing data were not readily accessible (42%), and existing data were poor quality, unreliable, or incomplete (32%, Q63). For data generators (sample size 8), the most impactful challenges were difficulty translating data or modeling work to public health practice (median 3.5/between moderately impactful and impactful), insufficient human resources with necessary skills (3/moderately impactful), and maintaining a timely and regular reporting (3/median moderately impactful, Q81). For data aggregators (sample size 11), several challenges were impactful. The most impactful challenges were: adopting standard definitions of variables of interest and maintaining a timely and regular reporting schedule with a median of 5/strongly impactful; obtaining a complete data set (no missing values), adapting to the changing needs of data users, and difficulty translating data/modeling work to public health practice (e.g. informing decision-makers on policy making, resource allocation, etc.) with a median of 4/impactful; insufficient human resources with necessary skills, insufficient computational resources, and insufficient funding with a median of 3/moderately impactful. Additionally, data aggregators faced a unique set of challenges from needing to access and harmonize other data sources. When asked to rate the impact of the limitations of primary datasets, most of the listed limitations had a median rating of 4/impactful: incompleteness of data (missing values), reporting frequency irregularities, anomalies in data, lack of standardization in data definitions (e.g. across jurisdictions, over time), lack of documentation from data generators on how data were collected, anomalies in the data, etc., and data accessibility—not in machine readable format (Q80).

20 tool users in our survey managed internal data for their institution, and they managed every data type we asked about, except for climate data. The top challenges to managing internal data were issues with data systems (e.g. ease of use, lack of interoperability between data systems) with a median of 4/impactful; inconsistencies in reporting (e.g. between jurisdictions or departments) and insufficient human resources with necessary skills as 3/moderately impactful; and political (national, local, or institutional) influences, difficulties with adopting standard definitions of variables of interest, difficulties with data sharing or establishing data use agreements, data privacy concerns, and insufficient funding as slightly 2/impactful (Q165).

2.4. Modeling

77% of tool users made use of models (Q213). The most common purposes for using models were to predict the near future (91%), to inform resource management (78%), and for situational awareness (74%, Q226). Out of those that used models, 78% used forecasting, 74% used projections, 52% used retrospective analyses, 39% used nowcasting, and 35% used optimization models (Q227). Among those that used each model type, all model types had a median rating of 4/very useful (Q228), as shown in Figure 5. However, projections had an interquartile range nearly twice as large as those of the other model types, with the quartile 1 value at 2/a little useful. The most influential challenges when using models were reliability of modeling results, timeliness of results, and competing priorities or limited resources as 4/impactful; and limited modeling at the geographic resolution of interest and limited modeling on subgroups of interest as 3/moderately impactful (Q230).

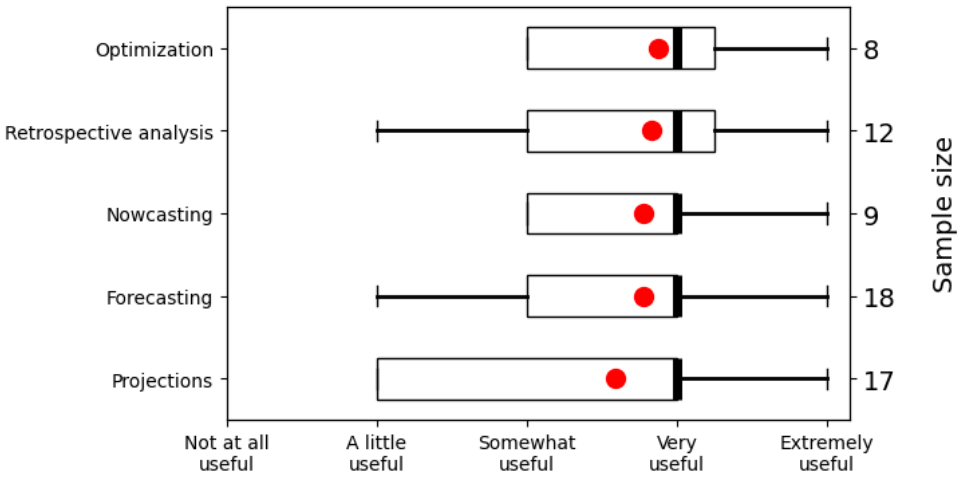

Figure 5. Plot showing the distributions of responses for Q228, usefulness of different model types. The red dots show the average value, which the y-axis values are sorted by. The sample sizes vary by choice because respondents were only shown model types that they used, as indicated in a prior question.

For model builders, the most impactful challenges were unknowable factors as 4/impactful; poor data quality, poor data availability, insufficient human resources with necessary skills, model performance issues, and difficulty translating modeling work to public health practice as 3/moderately impactful (Q58).

A wide variety of data types were useful for modelers. The median rating for the usefulness of each data type was 5/extremely useful for case or death data, other demographic data, hospitalizations data, vaccines allocated or administered, serological surveillance, and electronic medical records or individual-level health data, 4/very useful

for genomic surveillance data, testing data, other epidemiological data, health risk factors data, and other behavioral data, 3.5/between very and somewhat useful for COVID-19 response policy data and consumer data, and 3/somewhat useful for mobility data, wastewater surveillance data, and climate or environmental data (Q38, Figure 6).

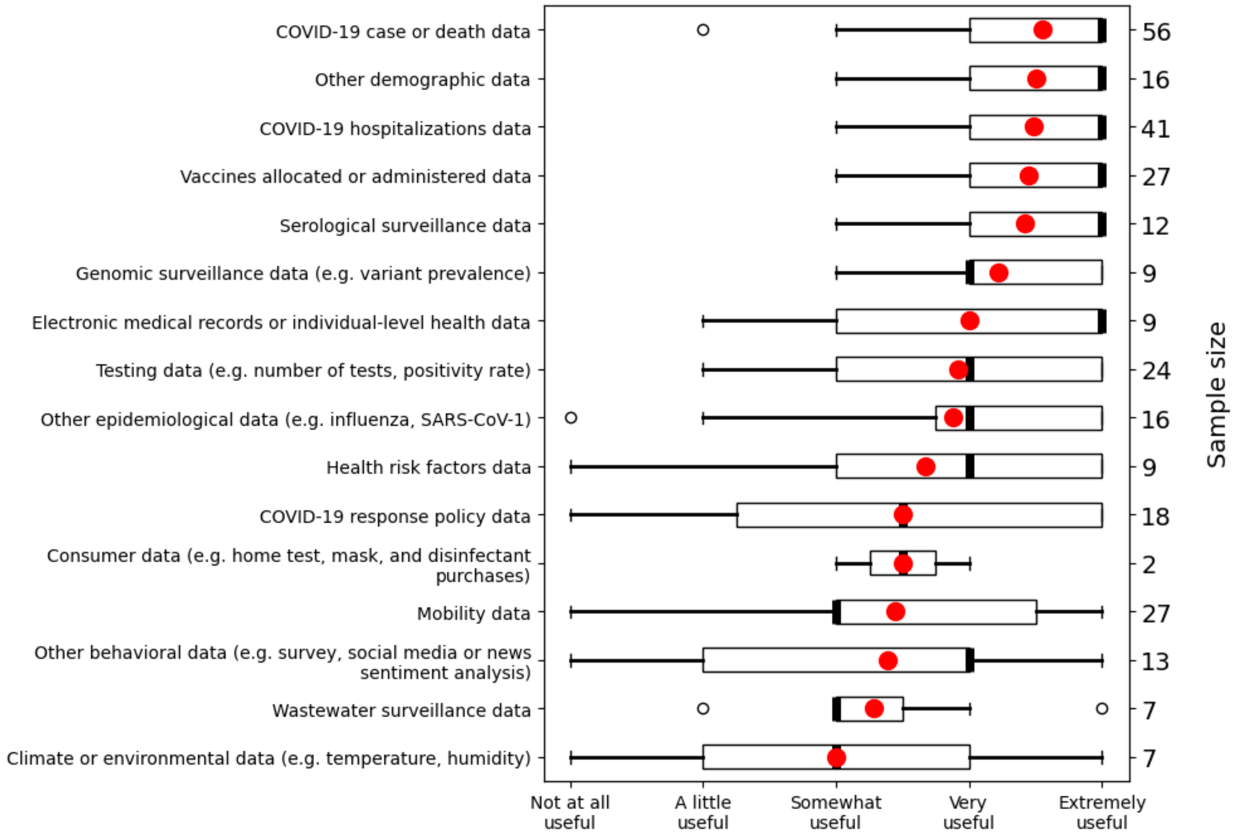

Figure 6. Plot showing the distributions of responses for Q38, usefulness of different data types for modelers. The red dots show the average value, which the y-axis values are sorted by. The sample sizes vary by choice because respondents were only shown data types that they used, as indicated in a prior question.

Among modelers, the top challenges of using data were data availability (desired data were never collected), incompleteness of data (missing values), anomalies in data, and timeliness of data (too much lag between when data are current and when it's made available) with a median of 4/impactful, and reporting frequency irregularities, biases in the data, data accessibility—not publicly available, lack of standardization in data definitions (e.g. across jurisdictions, over time), and lack of documentation from data curators on how data were collected, anomalies in the data, etc., with a median of 3/moderately impactful (Q44). When asked if a higher quality dataset for the following data types would have a high potential to improve their modeling work, the most common choices were COVID-19 case or death data (63%), hospitalizations data (49%), serological surveillance data (35%), testing data (35%), mobility data (32%), other

behavioral data (30%), vaccine data (25%), and wastewater surveillance data (25%, Q41).

In the open response section, respondents highlighted some of the challenges of modeling for COVID-19 response. Multiple respondents commented on the negative effect of the constantly changing situation, including variants, vaccination, and waning immunity, on producing timely and accurate models. One tool user commented that this was the first time their institution had used modeling, and it was disjointed and poorly communicated to stakeholders. Another tool user wished there had been models available that they could've applied to their local data. According to one tool user, there were too many competing models to be helpful during the pandemic, so they relied on working with trusted partners.

2.5. Translation

2.5.1. Top challenges to translational work

Challenges in translating data and modeling work to be useful for decision-makers were significant in COVID-19 response work. When tool users were asked to select the top 5 challenges to COVID-19 response, 37% selected difficulty translating data/modeling work to public health practice and 20% selected researchers unable to produce results fast enough for decision-making timelines (Q263). For tool builders, 44% selected difficulty translating data/modeling work to public health practice, 32% selected difficulty producing results fast enough for decision-making timelines, and 27% selected lack of direct communication with stakeholders (Q152).

2.5.2. Collaborations between researchers and decision-makers

A variety of challenges were impactful in collaborations between researchers and decision-makers. When tool users were asked to rate the extent that specified challenges impacted their selected collaboration with researchers, none of the challenges had a median higher than slightly impactful (Q249, Figure 7). The following challenges had a median rating of slightly impactful: limited time or capacity for collaborations with researchers, difficulty with data sharing or establishing data use agreements, lack of data or information available to answer our questions, researchers unable to produce results fast enough for decision-making timelines, and researchers struggling to adapt to our changing needs. However, the interquartile ranges for the top 3 challenges are quite large, with the first quartile at 1/not impactful and the third quartile at 4/impactful. For the top 5 challenges, the whiskers range to 5/strongly impactful. These measures of spread indicate that for some collaborations, each of these challenges are very impactful, although they may not be particularly common across many collaborations.

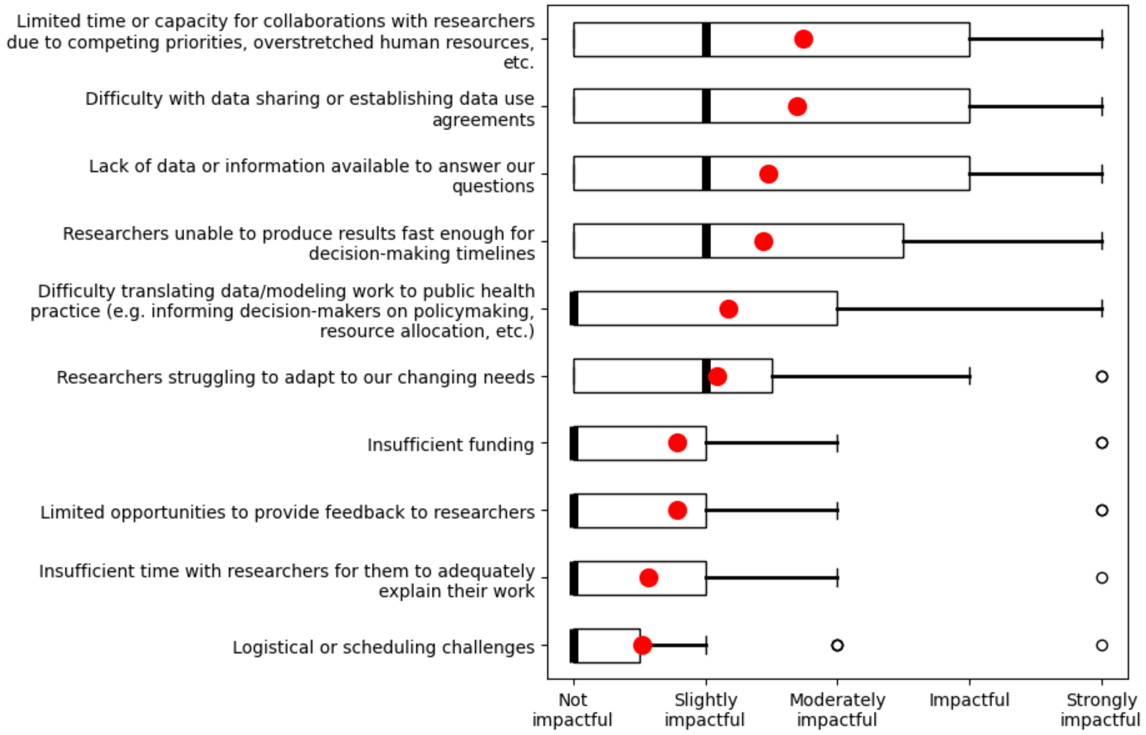

Figure 7. Plot showing the distributions of responses for Q249, challenges to collaborations reported by tool users. The red dots show the average value, which the y-axis values are sorted by. The sample size for this question was 23.

Tool builders reported that the specified challenges were more impactful. For builders, the following challenges had a moderately impactful rating: difficulty producing results fast enough for decision-making timelines, limited time or capacity for collaborations with stakeholders, and difficulty translating data/modeling work to public health practice (Q120, Figure 8). All other challenges had a median rating of slightly impactful, except for logistical or scheduling challenges. Compared to tool users, tool builders had even more challenges with large interquartile and whisker ranges. The top 6 have an interquartile range from 1/not impactful to 4/impactful, and all challenges except one have whiskers that range to 5/strongly impactful.

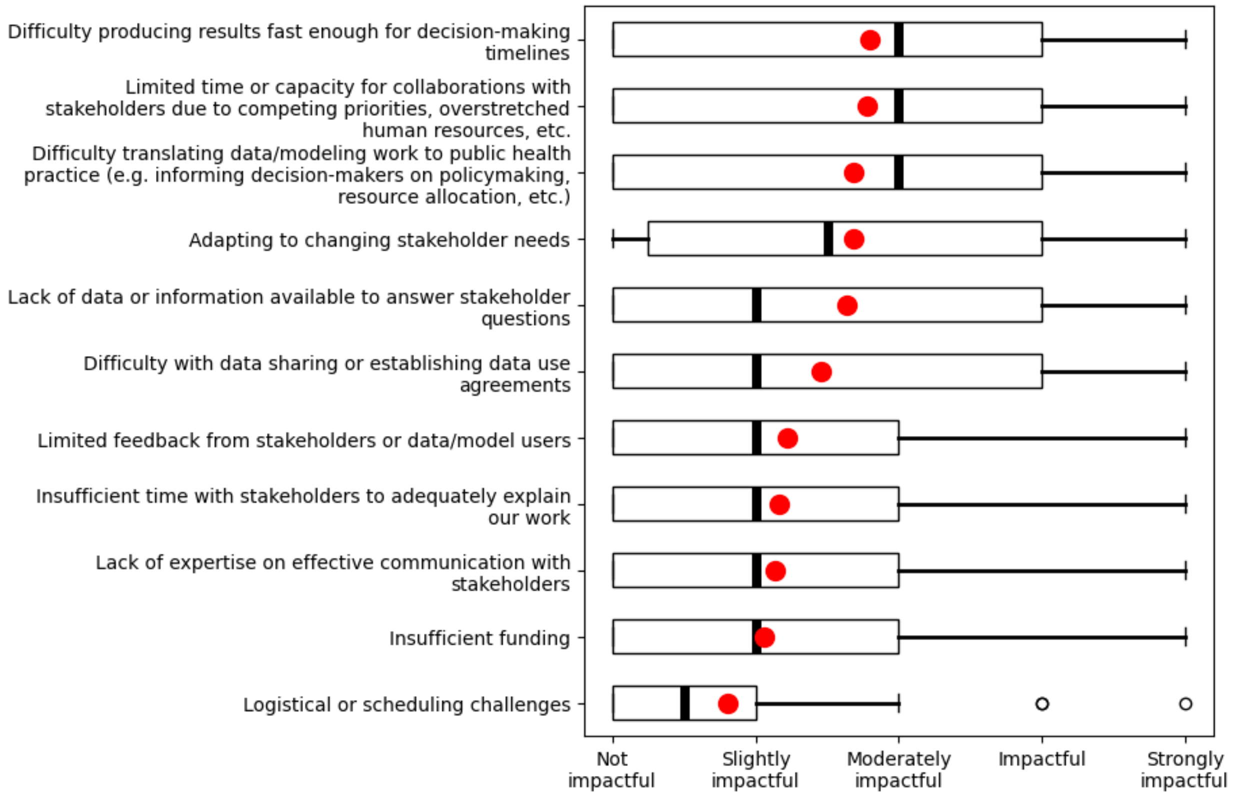

Figure 8. Plot showing the distributions of responses for Q120, challenges to collaborations reported by tool builders. The red dots show the average value, which the y-axis values are sorted by. The sample size for this question was 50.

According to our survey, tool builders largely adopted good practices for successful collaborations. Here, we will summarize the results from the questions asked to tool users about collaborations, but the results when builders were asked about their collaborations were similar. Among tool users who did not collaborate with researchers, the most impactful challenge was limited time or capacity (Q236). Among tool users that did collaborate with researchers, 65% had never communicated with those researchers before (Q240). In the open response section, two respondents highlighted the importance of having existing relationships before emergencies happen. 61% of tool users that collaborated with researchers provided data that were not publicly available. These collaborations provided tool users with access to modeling expertise (70%), synthesis of current COVID-19 data or information (70%), access to expert advice or policy recommendations (43%), and access to data that were not publicly available (30%, Q243). 65% of collaborators met 2 weeks or more often during the peak of their effort (Q244). Tool users reported that their needs had influence on the modeling tools researchers provided: 44% reported that researchers customized an existing model to meet their needs, 38% said they developed a model from scratch to meet our needs, and only 19% said that they did not adopt a model to our needs (Q245). Tool users reported that 62% of modelers asked for feedback once the model was operational, 56% did so in

the early stages of model development, and only 12% did not ask for feedback (Q246). For tool users that accessed data through their collaboration, the reported prevalence of researchers taking into account their needs and feedback was similar, except that data providers did not ask for feedback a bit more often than modelers (24% versus 19%, Q248). Tool users reported that they built a positive working relationship with researchers and that researchers communicated the uncertainty, limitations, and appropriate interpretations of their work (Q252). In the open response section, respondents noted that the changing needs of stakeholders over time and the variable needs of different stakeholders, which could be highly location dependent, presented a challenge.

2.5.3. Data and modeling tools' impact on decision-making

According to our respondents, data and modeling tools had substantial impact on decision-making. When tool users were asked to what extent their engagement with researchers informed their public health work, 70% said it informed their situational awareness, 57% said it directly impacted their decision-making, and only 13% said it did not impact their public health work (Q253). In the open response section, a few respondents commented on the value of providing situational awareness, explaining that although work that provides situational awareness often cannot be directly traced to a decision, this contextual information is critical for decision-makers. When tool users were asked if they interacted with relevant decision-makers, 77% said their team directly interacted with decision-makers, 63% said that the leadership of their institution interacted with decision-makers, and none said that their institution did not interact with decision-makers (Q257). Tool users overall felt that decision-makers took into account their recommendations and made decisions based on data, research, and/or public health knowledge where possible (Q258).

2.5.4. Lack of feedback for tool builders

While tool builders often got positive feedback on their work, they did not get specific feedback on if or what it was used for. When tool builders who collaborated with stakeholders were asked to what extent their work informed the stakeholders' public health work, 56% got positive feedback on the usefulness of their work but were not sure if or how it was used, 36% said that stakeholders informed them how their findings were used to inform their work, and 8% did not receive feedback on whether their work was useful (Q124). When tool builders were asked if their work was used to inform any of a comprehensive list of decision-making types, like resource allocation or implementation of NPIs, 28% said not that they are aware of (Q147). When this group was asked why they think their findings were not used for decision-making, 39% said they think it's likely that their findings were used to inform decision-making, they're just not aware of it (Q148). In the open response portion, several tool builders discussed the lack of specific feedback on their work from tool users. Multiple respondents commented that it was unclear if or how their work was used, and sometimes they did not find out how it was used until much later.

2.5.5. The US Forecast and Scenario Hub

To solicit feedback on the utility of the US COVID-19 Forecast and Scenario Hub, we asked the 7 respondents who used either of these resources to tell us if they were useful and how they were used. Out of the four responses, two discussed not using the Forecast Hub due to unreliability during critical points, especially once the impacts of new variants become dominant. One respondent did find the Forecast Hub to be useful for situational awareness. For the Scenario Modeling Hub, three out of four respondents said this resource was useful, and the only one that did not use this hub explained that anti-mask and vaccine sentiment in their jurisdiction made the scenarios not applicable. Respondents used the Scenario Modeling Hub to set vaccination recommendations and policies, for longer-term situational awareness, and for strategic planning. One respondent from a public health institution shared that just discussing the scenarios and why they were selected provided useful background information for discussions with leadership. This respondent also shared that the ensemble projection was useful for capturing the effects of different unknowns, and that seeing individual model predictions was useful for illustrating structural uncertainties about the future.

2.5.6. Science communication

In the open response portion, respondents outlined some of the difficulties in translating data and modeling tools for use by decision-makers. One tool builder cited "a fundamental disconnect about the goals of modeling." Another noted that there was a cultural disconnect between academics' desire for accuracy versus the faster, "good enough" answers wanted by decision-makers. Respondents also expressed the challenges of communicating the nuances of data-driven tools to decision-makers. One source of difficulty was the constantly changing nature of the COVID-19 pandemic, which meant that analysis of past data could not always produce reliable insights for the future. For example, the interpretation of testing data changed throughout the pandemic due to several factors, so identifying the hard thresholds for action desired by decision-makers was not feasible. In response to the failures of data-driven analyses to predict the future, some decision makers developed "an explicit preference for reactive decision-making," preferring to make judgements based on raw data rather than more sophisticated analyses. One tool builder shared that a decision-maker they worked with said that "I can only make decisions based on data, not speculation." Another related challenge was explaining uncertainty and setting realistic expectations for what models can do. One tool builder said that decision-makers also listening to untrained "experts" who were difficult to refute presented a challenge.

2.5.7. Impact of politics

In the multiple-choice sections of the survey, many respondents indicated that politics had influence on decision-making. When tool users were asked to rate the extent to which they agree with the statement "politics (national, local, or institutional) mainly dictated decision-making" the median was between "neither agree nor disagree" and "agree," with one-fourth of respondents choosing a rating of "agree" or "strongly agree" (Q258). Among tool builders that shared findings within their institution, the median rating for politics mainly dictating decision-making was "agree" (Q99). In the open

response section, several respondents commented on the oversize influence on politics on public health decision-making. In the words of one respondent, there were "always strong political undertones." One respondent shared that their visuals were used when it supported a political point, but were shared in a way that implied a causal link when the respondent did not make this claim. Another respondent said that their results were sometimes used for political expediency, so leaders could say that they were consulting scientists, but then their results were ignored when politically inconvenient.

2.6. Human and Financial Resources

Lack of human resources presented a significant challenge to COVID-19 response work, both for builders and users. Insufficient human resources was the third highest priority for users, selected by 50% (Q263), and was the second highest priority for builders, selected by 46% (Q152). This challenge was a little more frequently chosen by academics than other builders (50% versus 35%, Q152), and much more frequently chosen by public health institutions (PHIs) than other users (75% versus 33%, Q263). In particular, lack of relevant expertise was a bigger challenge for PHIs. For both using data and models, lack of expertise within their institution to interpret or use these tools had a median rating of moderately impactful for the PHI group, and not impactful for the rest of the users (Q191, Q230).

Acquiring funding also had a significant impact on COVID-19 response work, especially for builders. Among tool users, 17% selected insufficient funding as a top challenge, which was the 8th ranked choice out of 10 options (Q263). In contrast, 33% of builders selected insufficient funding as one of their top 5 overall challenges, which was the 6th ranked choice out of 16 options (Q152). When builders were asked if their selected project received funding, 54% selected funding was specifically awarded for this project, 35% selected funding for other projects was reallocated for this project, and about 20% said that the project was not funded (Q21). Among those that received funding specifically for their project, 36% were working for more than 3 months before they received funding, 34% were funded within 3 months of starting the project, and 30% got funding before they started working on the project (Q22). When asked why the project ended, 20% of builders selected because they ran out of funding (Q151).

Among builders, there were differences in funding access between the 7 data generators, 11 data aggregators, and 63 modelers. Data generators were most likely to get funding specifically for their project (88%), followed by data aggregators (64%), and then modelers (48%, Q21). Modelers were more likely to reallocate funds (43% versus 11%) or not receive funds for their project (22% versus 11%, Q21). Among those that received funding for their project, data generators were most likely to wait more than 3 months before receiving funding (57% versus 33% for modelers and 29% for aggregators, Q22).

2.7. Top Priorities for Investment

Both builders and users were asked to select their 7 top priorities for investment to improve future pandemic response out of the 22 data types and research objectives covered by this survey. Every respondent saw this question, so the sample size was 82 for tool builders and 30 for tool users. For both builders and users, determining the

effectiveness of different interventions (e.g. policies or vaccinations) or surveillance systems was most frequently chosen (60% builders, 63% users), followed by hospitalizations data (50% builders, 53% users, Q153, Q264). For builders, the next highest 3 priorities were situational awareness (50%), cases or deaths data (45%), and determining factors that impact transmission or severity of disease (44%, Q153). For users, the next priorities were optimal management or allocation of resources (53%), predictions of the near future (50%), cases or deaths data (47%), and genomic surveillance (47%, Q264). Note that the situational awareness answer choice was accidentally omitted from the users' priorities question. Among tool users, every data source was chosen by at least 10%, with several being chosen by more than a third of respondents. When comparing builders to users, users more frequently selected the following choices: resource allocation (53% versus 26%), predictions for the near future (50% versus 20%), genomic surveillance (47% versus 26%), and EMR or individual level health data (43% versus 21%). Builders more often selected serological surveillance data (33% versus 10%) and other behavioral data (24% versus 7%).

Respondents chose a wide variety of priorities. For builders, the distribution of the percent of respondents that selected each choice had a quartile 1 value of 16%, a median of 23%, and a quartile 3 value of 37%. For users, the quartile 1 value was 10%, the median was 23%, and the quartile 3 value was 47%. So for both groups, half of the answer choices were selected by more than 23% of respondents. For builders, three-fourths of the answer choices were selected by more than 16% of respondents, and for users, three-fourths were selected by more than 10% of respondents.

2.8. Subgroup Analyses

2.8.1. Public Health Institutions

We completed a subanalysis on the respondents that indicated that they were affiliated with a state or local public health institution (PHI) and compared the responses within this group to the rest of the respondents. 17 individuals affiliated with PHIs completed the survey, but note that the findings below are based on PHIs in the user branch, which has a sample size of 12. PHIs had different research priorities than the rest of user respondents. For the early phase of the pandemic, approximately January-April 2020, PHIs gave higher importance to learning which populations are most at risk (median 5/extremely important versus median very 4/important), learning about factors that impact transmission (average value 4.6 versus 3.5, median 5/extremely important versus 4/very important), and communicating information to the public (average 4.7 versus 3.8, median 5/extremely important for both, Q158). For the middle/control phase of the COVID-19 pandemic, approximately late 2020 to mid-2021, the difference between PHIs and other respondents increased for the importance of communicating information to the public (5/median extremely important versus 3/somewhat important, Q159), whereas for all other research objectives, PHIs were similar to other respondents.

PHI respondents had access to a wider range of data sources than other respondents. When asked to select the 5 most impactful challenges to COVID-19 response work, only 8% of PHIs selected data accessibility issues, compared to 44% among other users (Q263). PHIs that took our survey internally managed nearly every data type we asked

about, excluding only COVID-19 response policy data, mobility data, and climate data (Q164). 100% of PHI respondents managed case, death, and hospitalizations data, 90% managed testing data, 90% managed other epidemiological data, 90% electronic medical records or other individual health data, 70% genomic surveillance data, 70% vaccine data, 60% other demographic data, 50% health risk factors data, 40% wastewater surveillance data, 20% serological surveillance data, 20% consumer data (e.g. test purchases), and 10% other behavioral data (Q164). Some of these data types were frequently selected by tool users as data they wanted to use but weren't able to: EMR or individual-level health data (23%) health risk factors data (17%), and wastewater surveillance data (10%, Q192). In addition to having access to more data types, PHIs used a wider variety of data types. PHIs were much more likely to use EMR or individual-level health data (73% versus 44%), wastewater surveillance data (73% versus 39%), other demographic data (73% versus 33%), and health risk factors data (73% versus 28%, Q166).

2.8.2. Academia

We also analyzed how the 69 respondents that are affiliated with universities differed from other respondents. Academics were more likely to be working on research objectives that did not address immediate needs, like learning about the effectiveness of different policy interventions and/or surveillance systems (55% versus 31%), learning about factors that impact transmission (35% versus 15%), and learning about possible long-term scenarios (43% versus 31%, Q23). Notably, learning about the effectiveness of interventions was the top priority for future investment for both builders and users (Q153, Q264). Academics were also more likely to model subgroups, like medical facilities, schools, nursing homes, or prisons (50% versus 23%, Q36). Academics were also more likely to use alternative surveillance data: mobility data (48% versus 23%), serological surveillance data (22% versus 8%), genomic surveillance data (18% versus 0%), and wastewater surveillance data (14% versus 0%, Q37). When citing reasons why the selected project ended, academics were more likely to select burn-out from COVID-19 work (22% versus 9%) or ran out of funding (22% versus 9%, Q151).

Many of the academics in our survey formed direct collaborations with practitioners, and a substantial portion did not publish a paper on their selected project. 29% of academics did not publish in a peer-reviewed journal or post a preprint on their selected project by the time of this survey, fall 2023 (Q91). Of note, we do ask builders to select their most impactful project to report on, and we explicitly say that we are more interested in impact on decision-makers than on publications. 63% of academics directly communicated with stakeholders (Q91), in particular, academics were much more likely than other respondents to collaborate with public health institutions (67% versus 36%, Q108). Academics also reported that their team directly interacted with relevant decision-makers more often than other respondents (74% v 33%, Q98). Academics were more likely to develop a model or data collection process from scratch to meet stakeholder needs (models 52% versus 14%, Q116; data collection 67% versus 50%, Q118). However, academics were more likely to have less frequent communication with their collaborator, with 46% of academics communicating every 2 weeks or more often compared to 100% of the other 11 builders that collaborated (Q115). When asked to select the top 5 most

influential challenges to COVID-19 response work, academics were more likely to select lack of expertise on effective communication with stakeholders (13% versus 0%, Q152).
