# Supplementary material for: Data generation and modeling during COVID-19: utility, barriers, and priorities for future investments in public health response
Source: Front Public Health. 2026 Feb 23;14:1718094. doi: 10.3389/fpubh.2026.1718094 (PMC12968176; doi:10.3389/fpubh.2026.1718094)
Supplement: SUPPLEMENTARY Data Sheet 2 — Supplementary Material B: Survey Questions. This file contains a complete record of survey questions, answer choices, and logic. [file Data_Sheet_2.pdf]

Supplementary Material B:
Survey Questions

Table of Contents

Eligibility and Consent 3
Introduction 4
Building Datasets or Models – Introduction 9
Building Datasets or Models – Methodology – Modelers 12
Geographic Coverage 16
Data Used 33
Methods 46
Challenges 54
Building Datasets or Models – Methodology – Data Aggregators or Generators . 57
Geographic Coverage 5
Methods 19
Challenges 23
Building Datasets or Models – Translation 29
News or Social Media 31
Findings Shared Within Your Institution 33
Communication with Stakeholders 35
Communication with Researchers 53
Building Datasets and Models – Concluding Questions 67
Using Datasets or Models – Introduction 75
Using Datasets or Models – Use of Data 80
Challenges of Using Data 96
Data Not Used 117
Using Datasets or Models – Use of Models 138

Internally Developed Models **140**

Using Models **146**

Using Datasets or Models – Collaborations with Researchers ***153***

Using Datasets or Models – Concluding Questions ***166***

Information Sources **166**

Impact on Decision-makers **168**

Concluding Questions **170**

Eligibility and Consent

Q1 Did you generate or use COVID-19 modeling, data, or surveillance tools for the US?

- ◯ Yes
- ◯ No

Q2 Are you a US resident?

- ◯ Yes
- ◯ No

Display question:

If Q2 = No

Due to funding restrictions we are unable to provide the $100 gift card to non-US residents. However, we welcome and encourage you to still fill out the survey.

Q3 Does your institution allow you to accept compensation, in the form of a $100 gift card, for completing this survey? (Federal or state employees often cannot accept payment or gifts)

- ◯ Yes
- ◯ No

Q4 Please enter your work email address. Use a .edu, .gov, or .org email if you have one in order to enable faster validation of your survey response.   This information will only be used to validate your response and send the gift card.

____

Introduction

The following personal information will only be used to confirm the validity of your survey response. When survey results are publicly shared, there will not be any identifying information for individuals or their institutions.

Q5 Please enter your name.

____

Q6 Please provide the name of the institution that is your primary affiliation.

____

Q7 What type of institution are you primarily affiliated with?

- ☐ University
- ☐ State or local public health department
- ☐ CDC
- ☐ Other government agency, contractor, or office involved in COVID-19 policymaking
- ☐ Healthcare institution
- ☐ Other for profit company
- ☐ Other nonprofit company
- ☐ Other, please specify ____

Q8 Is your institution primarily doing COVID-19 research (e.g. academia) or response work (e.g. public health institutions, healthcare institutions)?

- ○ Research
- ○ Response

Display this question:

If Q7 = Healthcare institution

Q9 What type of healthcare institution are you affiliated with?

- ☐ Hospital
- ☐ Health clinic or outpatient care facility
- ☐ Nursing home
- ☐ Rehabilitation facility
- ☐ Other institution devoted to the care of sick, infirm, or aged person
- ☐ Other, please specify

____

Display this question:

If Q7 = Other government agency, contractor, or office involved in COVID-19 policymaking

Q10 Which level of government are you affiliated with?

- ☐ Mayor's office or equivalent
- ☐ City council members or equivalent
- ☐ Local or municipal courts
- ☐ Other local level, please specify
- ☐ Governor's office
- ☐ State congress
- ☐ State courts
- ☐ Other state level, please specify

____

- ☐ President's office
- ☐ US Center for Disease Control
- ☐ US Department of Health and Human Services
- ☐ Other executive branch department
- ☐ Federal congress
- ☐ Federal courts

☐ Other federal level, please specify ____

☐ Tribal jurisdiction

☐ Other, please specify ____

Q11 What is your job title or role at your institution?

____

Q12 Are you willing to be contacted by the research team for follow-up questions on your responses?

○ Yes

○ No

Please answer the following question carefully. It will determine whether the rest of the survey accurately captures your work.

Q13 What was your primary role in COVID-19 surveillance and response efforts? If you work in academia, your default response would likely be “building” unless your role included making decisions about response, such as determining university COVID-19 response policy. If you work in a practice or response focused organization (public health institution, healthcare institution, etc.), your default response will likely be either “using” or “both”. An exception would be if your role was primarily focused on building models or datasets, and you were not making decisions or directing response efforts based on these tools.

○ Building models, tools, and/or data sets to support COVID-19 surveillance and response efforts, including adapting existing methods to inform COVID-19 response (usually

academics, modelers, data collectors). This includes if you were developing tools for use by decision-makers

◯ Using models, tools, and/or data sets to make decisions or direct response efforts (usually public health practitioners, policymakers)

◯ I had significant involvement in both building models, tools, and/or data sets AND using them to make decisions or direct response efforts.

Building Datasets or Models – Introduction

The “Building Datasets or Models” sections of the survey are shown to respondents that selected “Building models, tools, and/or datasets” for Q13

This survey will consist of a few introductory questions and a few concluding questions, with the bulk of the survey divided into 2 sections: Methodology and Translation. The methodology section should take 10-15 minutes to complete, and the time needed to complete the translation section could be up to 15 minutes, depending on how much translational work you did for your selected project (e.g. communicating with media or stakeholders).

Q14 Before the COVID-19 pandemic, had you ever worked on public health-related projects?

◯ Yes, I had worked in epidemiology specifically

◯ Yes, I had worked in other fields in public health

◯ No, I had not worked in public health

Q15 To what extent did your COVID-19 work focus on the US?

◯ US only

◯ Mostly US, but some international

◯ Global, including US

Q16 Which of the following US COVID-19 research efforts did you engage in? Select all that apply.

- ☐ Modeling or other forms of data analysis
- ☐ Data Aggregation: collecting and centralizing existing data for ease of access (e.g. JHU CSSE, Delphi Epidata API)
- ☐ Data Generation: generating data that was not previously available (e.g. Facebook COVID-19 Trends and Impact Survey, sequencing to produce genomic data, conducting wastewater surveillance)

For the remainder of the survey, you will answer questions based on one selected project. We recommend you select the most impactful project that you led.   In this survey we are primarily interested in "impact" on decision-makers (e.g. policymakers, public health institutions, healthcare institutions), and secondarily on academic impact (e.g. publications, citations). To ensure that only one survey response is provided per project, we recommend the lead of the project (e.g. principal investigator, lead author, etc.) completes the survey, or that the project lead designates someone to complete the survey on their behalf.

Q17 What kind of effort is your selected project?

- ○ Modeling or other forms of data analysis
- ○ Data Aggregation: collecting and centralizing existing data for ease of access (e.g. JHU CSSE, Delphi Epidata API)
- ○ Data Generation: generating data that was not previously available (e.g. Facebook COVID-19 Trends and Impact Survey, sequencing to produce genomic data, conducting wastewater surveillance)

Q18 In this survey, we want to capture only one response per project. To help us identify “duplicate” responses (i.e., multiple people reporting about the same project), please provide a distinctive 1-2 sentence description of the project. For example, “We submitted a 1 to 4 week forecasting model for all US states to the US Forecasting hub with the model name JHU_CSSE-DECOM.”

____

____

Q19 Please provide a link to your most recent publication or documentation on this project.

____

When answering these questions about your selected project, if the answers to these questions changed over the lifespan of the project, please answer the questions based on the peak operation of the project.

Q20 How large was the team working on this project at its peak?

- ◯ Less than 3 people
- ◯ 3-5 people
- ◯ 5-10 people
- ◯ 10-15 people
- ◯ 15-30 people
- ◯ More than 30 people

Q21 Did this project receive funding?

- ☐ Funding was awarded specifically for this project
- ☐ Funding for other projects was reallocated towards this project
- ☐ No
- ☐ I don’t know

Display this question:

If Q21 = Funding was awarded specifically for this project

Q22 How long was the gap between when you started working on this project and when you received funding?

- ○ We got funding before we began working on this project
- ○ We were funded within 3 months of beginning this project
- ○ We were working on this project for more than 3 months before getting funding

Building Datasets or Models – Methodology – Modelers

This section is shown if “Modeling or other forms of data analysis” was selected for Q16

Continue to answer these questions based on your one selected project.

Q23 Which of the following research objectives did your project address?

- ☐ To learn about the current situation and increase situational awareness
- ☐ To predict the near future
- ☐ To obtain virus and transmission characteristics (e.g. transmissibility, infection fatality ratio, generation time, asymptomatic proportion of cases)
- ☐ To learn about factors that impact SARS-CoV-2 transmission
- ☐ To learn about the effectiveness of vaccination
- ☐ To learn about the effectiveness of different policy interventions and/or surveillance systems
- ☐ To learn about possible long-term scenarios
- ☐ To learn which populations are most at-risk
- ☐ To inform resource management (e.g. hospital capacity, medical supplies, testing capacity, vaccine allocation)
- ☐ To communicate information about COVID-19 and/or risk reduction behaviors to the public
- ☐ Other, please specify ____

Q24 Which of the following types of modeling did your project include?

☐ Retrospective analysis: analyzes past data to better understand characteristics of SARS-CoV-2 or COVID-19 (such as drivers of COVID-19 dynamics or the impact of interventions)

☐ Nowcasting: focused on understanding current data, usually addressing reporting issues

☐ Forecasting: predicts near future outcomes without making assumptions about how the situation will change in the longer-term

☐ Projections: predicts longer-term outcomes, which requires making assumptions about the future (e.g. new variants, future interventions)

☐ Optimization: the model outputs provide recommendations for optimal decisions, like how to best allocate resources

☐ Other, please specify ____

Display this question:

If Q24 = Retrospective analysis: analyzes past data to better understand characteristics of SARS-CoV-2 or COVID-19 (such as drivers of COVID-19 dynamics or the impact of interventions)

Or Q24 = Nowcasting: focused on understanding current data, usually addressing reporting issues

Or Q24 = Forecasting: predicts near future outcomes without making assumptions about how the situation will change in the longer-term

Or Q24 = Projections: predicts longer-term outcomes, which requires making assumptions about the future (e.g. new variants, future interventions)

Q25 What types of methodologies did you use for this model?

- [ ] Compartmental (SIR and variations)
- [ ] Statistical
- [ ] Agent-based
- [ ] Other, please specify ____

Display this question:

If Q24 = Retrospective analysis: analyzes past data to better understand characteristics of SARS-CoV-2 or COVID-19 (such as drivers of COVID-19 dynamics or the impact of interventions)

Or Q24 = Nowcasting: focused on understanding current data, usually addressing reporting issues

Or Q24 = Forecasting: predicts near future outcomes without making assumptions about how the situation will change in the longer-term

Or Q24 = Projections: predicts longer-term outcomes, which requires making assumptions about the future (e.g. new variants, future interventions)

Q26 Did you use any of the following statistical methods?

- ☐ Regression
- ☐ Support vector machines
- ☐ Decision tree-based methods
- ☐ Neural networks
- ☐ Bayesian methods
- ☐ Other, please specify ____
- ☐ We did not use statistical methods

Geographic Coverage

Q27 What was your model's geographic resolution?

- ▢ National
- ▢ Regional (smaller than national, larger than state)
- ▢ State
- ▢ County
- ▢ City or metropolitan area
- ▢ Smaller than county or city level (e.g. census tract)
- ▢ Other, please specify

____

Display this question:

If Q27 = Regional (smaller than national, larger than state)

Q28 Which regions best describe your geographic coverage?

- ☐ New England (Connecticut, Maine, Massachusetts, New Hampshire, Rhode Island, Vermont)
- ☐ Middle Atlantic (New Jersey, New York, Pennsylvania)
- ☐ East North Central (Indiana, Illinois, Michigan, Ohio, Wisconsin)
- ☐ West North Central (Iowa, Kansas, Minnesota, Missouri, Nebraska, North Dakota, South Dakota)
- ☐ South Atlantic (Delaware, DC, Florida, Georgia, Maryland, North Carolina, South Carolina, Virginia, West Virginia)
- ☐ East South Central (Alabama, Kentucky, Mississippi, Tennessee)
- ☐ West South Central (Arkansas, Louisiana, Oklahoma, Texas)
- ☐ Mountain (Arizona, Colorado, Idaho, New Mexico, Montana, Utah, Nevada, Wyoming)
- ☐ Pacific (Alaska, California, Hawaii, Oregon, Washington)
- ☐ Puerto Rico
- ☐ Guam
- ☐ US Virgin Islands
- ☐ Northern Mariana Islands
- ☐ American Samoa

☐ Other, please specify

____

Display this question:

If Q27 = State

Q29 Which option best describes your state level geographic coverage?

○ All states, including territories (Puerto Rico, Guam, American Samoa, Northern Mariana Islands, etc.)

○ All states and Puerto Rico

○ All states, no territories

○ States in certain regions of the US

○ 5 states or less

○ Other, please specify ____

Display this question:

If Q29 = States in certain regions of the US

Q30 Which regions best describe your state level geographic coverage?

- ☐ New England (Connecticut, Maine, Massachusetts, New Hampshire, Rhode Island, Vermont)
- ☐ Middle Atlantic (New Jersey, New York, Pennsylvania)
- ☐ East North Central (Indiana, Illinois, Michigan, Ohio, Wisconsin)
- ☐ West North Central (Iowa, Kansas, Minnesota, Missouri, Nebraska, North Dakota, South Dakota)
- ☐ South Atlantic (Delaware, DC, Florida, Georgia, Maryland, North Carolina, South Carolina, Virginia, West Virginia)
- ☐ East South Central (Alabama, Kentucky, Mississippi, Tennessee)
- ☐ West South Central (Arkansas, Louisiana, Oklahoma, Texas)
- ☐ Mountain (Arizona, Colorado, Idaho, New Mexico, Montana, Utah, Nevada, Wyoming)
- ☐ Pacific (Alaska, California, Hawaii, Oregon, Washington)
- ☐ Puerto Rico
- ☐ Guam
- ☐ US Virgin Islands
- ☐ Northern Mariana Islands
- ☐ American Samoa

☐ Other, please specify

____

Display this question:

If Q29 = 5 states or less

Q31 Which US states?

- ☐ Alabama
- ☐ Alaska
- ☐ Arizona
- ☐ Arkansas
- ☐ California
- ☐ Colorado
- ☐ Connecticut
- ☐ Delaware
- ☐ District of Columbia
- ☐ Florida
- ☐ Georgia
- ☐ Hawaii
- ☐ Idaho
- ☐ Illinois
- ☐ Indiana

- [ ] Iowa
- [ ] Kansas
- [ ] Kentucky
- [ ] Louisiana
- [ ] Maine
- [ ] Maryland
- [ ] Massachusetts
- [ ] Michigan
- [ ] Minnesota
- [ ] Mississippi
- [ ] Missouri
- [ ] Montana
- [ ] Nebraska
- [ ] Nevada
- [ ] New Hampshire
- [ ] New Jersey

- [ ] New Mexico
- [ ] New York
- [ ] North Carolina
- [ ] North Dakota
- [ ] Ohio
- [ ] Oklahoma
- [ ] Oregon
- [ ] Pennsylvania
- [ ] Puerto Rico
- [ ] Rhode Island
- [ ] South Carolina
- [ ] South Dakota
- [ ] Tennessee
- [ ] Texas
- [ ] Utah
- [ ] Vermont

☐ Virginia

☐ Washington

☐ West Virginia

☐ Wisconsin

☐ Wyoming

Display this question:

If Q27 = County

Q32 Which option best describes your county level geographic coverage?

○ All or most counties in the US and equivalent resolution in the territories

○ All or most counties in the in US, excluding territories

○ All or most counties in certain regions of the US

○ All or most counties in 5 or less states

○ If less than 5 counties, please list the counties
____

○ Other, please specify ____

Display this question:

If Q32 = All or most counties in certain regions of the US

Q33 Which regions best describe your county level geographic coverage?

- ☐ New England (Connecticut, Maine, Massachusetts, New Hampshire, Rhode Island, Vermont)
- ☐ Middle Atlantic (New Jersey, New York, Pennsylvania)
- ☐ East North Central (Indiana, Illinois, Michigan, Ohio, Wisconsin)
- ☐ West North Central (Iowa, Kansas, Minnesota, Missouri, Nebraska, North Dakota, South Dakota)
- ☐ South Atlantic (Delaware, DC, Florida, Georgia, Maryland, North Carolina, South Carolina, Virginia, West Virginia)
- ☐ East South Central (Alabama, Kentucky, Mississippi, Tennessee)
- ☐ West South Central (Arkansas, Louisiana, Oklahoma, Texas)
- ☐ Mountain (Arizona, Colorado, Idaho, New Mexico, Montana, Utah, Nevada, Wyoming)
- ☐ Pacific (Alaska, California, Hawaii, Oregon, Washington)
- ☐ Puerto Rico
- ☐ Guam
- ☐ US Virgin Islands
- ☐ Northern Mariana Islands
- ☐ American Samoa

☐ Other, please specify

____

Display this question:

If Q32 = All or most counties in 5 or less states

Q34 Which US states best describe your county level coverage?

- ☐ Alabama
- ☐ Alaska
- ☐ Arizona
- ☐ Arkansas
- ☐ California
- ☐ Colorado
- ☐ Connecticut
- ☐ Delaware
- ☐ District of Columbia
- ☐ Florida
- ☐ Georgia
- ☐ Hawaii
- ☐ Idaho
- ☐ Illinois
- ☐ Indiana

- [ ] Iowa
- [ ] Kansas
- [ ] Kentucky
- [ ] Louisiana
- [ ] Maine
- [ ] Maryland
- [ ] Massachusetts
- [ ] Michigan
- [ ] Minnesota
- [ ] Mississippi
- [ ] Missouri
- [ ] Montana
- [ ] Nebraska
- [ ] Nevada
- [ ] New Hampshire
- [ ] New Jersey

- [ ] New Mexico
- [ ] New York
- [ ] North Carolina
- [ ] North Dakota
- [ ] Ohio
- [ ] Oklahoma
- [ ] Oregon
- [ ] Pennsylvania
- [ ] Puerto Rico
- [ ] Rhode Island
- [ ] South Carolina
- [ ] South Dakota
- [ ] Tennessee
- [ ] Texas
- [ ] Utah
- [ ] Vermont

- ☐ Virginia
- ☐ Washington
- ☐ West Virginia
- ☐ Wisconsin
- ☐ Wyoming

Display this question:

If Q27 = Smaller than county or city level (e.g. census tract)

Or Q27 = City or metropolitan area

Q35 Please briefly describe the geographic resolution and coverage of your model (e.g. Census tract level in Baltimore city)

____

Q36 Did you explicitly model any of the following populations?

- ☐ Prisons
- ☐ Schools, excluding universities
- ☐ Universities
- ☐ Nursing homes
- ☐ Medical facilities
- ☐ Frontline workers (medical personnel)
- ☐ Essential workers (non-medical personnel)
- ☐ Other, please specify ____
- ☐ ⊗None of the above

Data Used

Q37 Which of the following data sources did you use for this modeling project?

- ☐ COVID-19 case or death data
- ☐ COVID-19 hospitalizations data
- ☐ Other epidemiological data (e.g. influenza, SARS-CoV-1)
- ☐ Vaccines allocated or administered data
- ☐ Testing data (e.g. number of tests, positivity rate)
- ☐ Serological surveillance data
- ☐ Electronic medical records or individual-level health data
- ☐ Genomic surveillance data (e.g. variant prevalence)
- ☐ Wastewater surveillance data
- ☐ Mobility data
- ☐ Other behavioral data (e.g. survey, social media or news sentiment analysis)
- ☐ COVID-19 response policy data
- ☐ Climate or environmental data (e.g. temperature, humidity)
- ☐ Consumer data (e.g. home test, mask, and disinfectant purchases)

- ☐ Health risk factors data
- ☐ Other demographic data
- ☐ Other, please specify ______________________
- ☐ Other, please specify ______________________
- ☐ Other, please specify ______________________

Q38 Rate the usefulness of each data type for your modeling work.

Only data types selected in Q37 are shown

	Not at all useful	A little useful	Somewhat useful	Very useful	Extremely useful
COVID-19 case or death data	◯	◯	◯	◯	◯
COVID-19 hospitalizations data	◯	◯	◯	◯	◯
Other epidemiological data (e.g. influenza, SARS-CoV-1)	◯	◯	◯	◯	◯
Vaccines allocated or administered data	◯	◯	◯	◯	◯
Testing data (e.g. number of tests, positivity rate)	◯	◯	◯	◯	◯
Serological surveillance data	◯	◯	◯	◯	◯
Electronic medical records or individual-level health data	◯	◯	◯	◯	◯
Genomic surveillance data (e.g. variant prevalence)	◯	◯	◯	◯	◯
Wastewater surveillance data	◯	◯	◯	◯	◯
Mobility data	◯	◯	◯	◯	◯
Other behavioral data (e.g. survey, social media or news sentiment analysis)	◯	◯	◯	◯	◯
COVID-19 response policy data	◯	◯	◯	◯	◯
Climate or environmental data (e.g. temperature, humidity)	◯	◯	◯	◯	◯
Consumer data (e.g. home test, mask, and disinfectant purchases)	◯	◯	◯	◯	◯

Health risk factors data	○	○	○	○	○
Other demographic data	○	○	○	○	○
Other, please specify	○	○	○	○	○
Other, please specify	○	○	○	○	○
Other, please specify	○	○	○	○	○

Q39 Did you use datasets from any of the following sources?

- [ ] JHU CSSE COVID-19 case and death data or dashboard
- [ ] HHS Hospitalization Data
- [ ] Delphi Epidata API (COVIDcast data, data about other diseases)
- [ ] The COVID Tracking Project
- [ ] Apple Mobility Trends Reports
- [ ] Talus Analytics policy/intervention data (COVID AMP)
- [ ] Oxford COVID-19 Government Response Tracker (OxCGRT)
- [ ] SafeGraph mobility data
- [ ] Covariants.org
- [ ] GISAID variant tracker
- [ ] US Census
- [ ] Real-time Assessment of Community Transmission (REACT) Studies – Imperial College London
- [ ] Cuebiq mobility data
- [ ] Policy/intervention data

- [ ] Epidemiological data (e.g. cases or deaths, including for a particular sub-group such as prisons or colleges)
- [ ] Mask-wearing survey data
- [ ] Other data from the New York Times
- [ ] Wastewater surveillance data
- [ ] Testing data
- [ ] Vaccination data
- [ ] Epidemiological data (e.g. cases, deaths)
- [ ] Seroprevalence data
- [ ] Other data from CDC
- [ ] Vaccine data
- [ ] Other data from Our World in Data
- [ ] COVID-19 Trends and Impact Survey
- [ ] Mobility data
- [ ] Other data from Facebook
- [ ] Community Mobility Reports

- ☐ Search Trends
- ☐ Other data from Google
- ☐ Other, please specify ____
- ☐ ⊗None of the above

Q40 What portion of the data you used was publicly available?

- ○ None
- ○ Some, but less than half
- ○ About half
- ○ Most
- ○ All

Q41 Would a higher quality dataset for any of the following inputs have a high potential to improve your model?

- ☐ COVID-19 case or death data
- ☐ COVID-19 hospitalizations data
- ☐ Other epidemiological data (e.g. influenza, SARS-CoV-1)
- ☐ Vaccines allocated or administered data
- ☐ Testing data (e.g. number of tests, positivity rate)
- ☐ Serological surveillance data
- ☐ Electronic medical records or individual-level health data
- ☐ Genomic surveillance data (e.g. variant prevalence)
- ☐ Wastewater surveillance data
- ☐ Mobility data
- ☐ Other behavioral data (e.g. survey, social media or news sentiment analysis)
- ☐ COVID-19 response policy data
- ☐ Climate or environmental data (e.g. temperature, humidity)
- ☐ Consumer data (e.g. home test, mask, and disinfectant purchases)
- ☐ Health risk factors data

☐ Other demographic data

☐ Other, please specify ____

Display this question:

If Q24 = Retrospective analysis: analyzes past data to better understand characteristics of SARS-CoV-2 or COVID-19 (such as drivers of COVID-19 dynamics or the impact of interventions)

Or Q24 = Nowcasting: focused on understanding current data, usually addressing reporting issues

Or Q24 = Forecasting: predicts near future outcomes without making assumptions about how the situation will change in the longer-term

Or Q24 = Projections: predicts longer-term outcomes, which requires making assumptions about the future (e.g. new variants, future interventions)

Q42 Did you calibrate or fit your model to any of the above data?

☐ Yes, the model was statistically trained or fitted to data

☐ Yes, we performed posthoc calibration of outputs

☐ Other method of calibration or fitting

☐ No

Display this question:

If Q24 = Optimization: the model outputs provide recommendations for optimal decisions, like how to best allocate resources

Q43 Was your optimization model data-driven?

○ Yes

○ No

Q44 Please rate to what extent each of the following challenges impacted your efforts to use data.

	Not Impactful	Slightly Impactful	Moderately Impactful	Impactful	Strongly Impactful
Anomalies in data	○	○	○	○	○
Incompleteness of data (missing values)	○	○	○	○	○
Reporting frequency irregularities	○	○	○	○	○
Timeliness of data (too much lag between when data is current and when it's made available)	○	○	○	○	○
Data availability: desired data was never collected	○	○	○	○	○
Data accessibility: not publicly available	○	○	○	○	○
Data accessibility: not in machine readable format	○	○	○	○	○
Data accessibility: cost prohibitive (data available but too expensive)	○	○	○	○	○
Data accessibility: privacy or data use agreement issues	○	○	○	○	○
Lack of standardization in data definitions (e.g. across jurisdictions, over time)	○	○	○	○	○
Sampling biases in the data	○	○	○	○	○
Lack of documentation from data curators on how data was collected, anomalies in the data, etc.	○	○	○	○	○

Q45 Were there any other significant challenges that impacted your efforts to use data?

- ◯ Yes
- ◯ No

Display this question:

If Q45 = Yes

Q46 Please list and rate the impact of any other significant challenges that impacted your efforts to use data.

	Not impactful	Slightly impactful	Moderately impactful	Impactful	Strongly impactful
Challenge 1:	◯	◯	◯	◯	◯
Challenge 2:	◯	◯	◯	◯	◯
Challenge 3:	◯	◯	◯	◯	◯
Challenge 4:	◯	◯	◯	◯	◯
Challenge 5:	◯	◯	◯	◯	◯

Methods

Display this question:

If Q24 = Nowcasting: focused on understanding current data, usually addressing reporting issues

Or Q24 = Forecasting: predicts near future outcomes without making assumptions about how the situation will change in the longer-term

Or Q24 = Projections: predicts longer-term outcomes, which requires making assumptions about the future (e.g. new variants, future interventions)

Q47 What were the target prediction variables of your model?

- ☐ Infections
- ☐ Reported cases
- ☐ Deaths
- ☐ Hospitalizations
- ☐ ICU admissions
- ☐ Rt
- ☐ Peak cases/deaths
- ☐ Growth rate
- ☐ Other, please specify ________________

Display this question:

If Q24 = Nowcasting: focused on understanding current data, usually addressing reporting issues

Or Q24 = Forecasting: predicts near future outcomes without making assumptions about how the situation will change in the longer-term

Or Q24 = Projections: predicts longer-term outcomes, which requires making assumptions about the future (e.g. new variants, future interventions)

Q48 Was your target variable continuous or categorical?

- ☐ Continuous
- ☐ Categorical
- ☐ Other, please specify ____

Display this question:

If Q24 = Retrospective analysis: analyzes past data to better understand characteristics of SARS-CoV-2 or COVID-19 (such as drivers of COVID-19 dynamics or the impact of interventions)

Or Q24 = Nowcasting: focused on understanding current data, usually addressing reporting issues

Or Q24 = Forecasting: predicts near future outcomes without making assumptions about how the situation will change in the longer-term

Or Q24 = Projections: predicts longer-term outcomes, which requires making assumptions about the future (e.g. new variants, future interventions)

Q49 How did you express quantitative uncertainty?

- ☐ Confidence or prediction intervals on the target variable
- ☐ Confidence or prediction intervals on parameters
- ☐ Sensitivity analysis
- ☐ Scenario analysis
- ☐ Other, please specify ____
- ☐ We did not express quantitative uncertainty

Display this question:

If Q24 = Retrospective analysis: analyzes past data to better understand characteristics of SARS-CoV-2 or COVID-19 (such as drivers of COVID-19 dynamics or the impact of interventions)

Or Q24 = Nowcasting: focused on understanding current data, usually addressing reporting issues

Or Q24 = Forecasting: predicts near future outcomes without making assumptions about how the situation will change in the longer-term

Or Q24 = Projections: predicts longer-term outcomes, which requires making assumptions about the future (e.g. new variants, future interventions)

Q50 Did you evaluate the performance of your model?

▢ Yes, with error metrics comparing point predictions to ground truth data

▢ Yes, by visual assessment

▢ Other, please specify ____

▢ No

Display this question:

If Q24 = Nowcasting: focused on understanding current data, usually addressing reporting issues

Or Q24 = Forecasting: predicts near future outcomes without making assumptions about how the situation will change in the longer-term

Or Q24 = Projections: predicts longer-term outcomes, which requires making assumptions about the future (e.g. new variants, future interventions)

Q51 Did you use any error metric that evaluates the performance of uncertainty estimates, like confidence intervals or quantiles?

○ Yes

○ No

Display this question:

If Q24 = Nowcasting: focused on understanding current data, usually addressing reporting issues

Or Q24 = Forecasting: predicts near future outcomes without making assumptions about how the situation will change in the longer-term

Or Q24 = Projections: predicts longer-term outcomes, which requires making assumptions about the future (e.g. new variants, future interventions)

Q52 Did your model provide outcomes (such as cases, deaths, or another target variable) broken down by specific sub-groups?

- ▢ Race or ethnicity
- ▢ Age
- ▢ Frontline workers (medical personnel)
- ▢ Essential workers (non-medical personnel)
- ▢ Other, please specify ____
- ▢ ⊗None of the above

Display this question:

If Q24 = Nowcasting: focused on understanding current data, usually addressing reporting issues

Or Q24 = Forecasting: predicts near future outcomes without making assumptions about how the situation will change in the longer-term

Or Q24 = Projections: predicts longer-term outcomes, which requires making assumptions about the future (e.g. new variants, future interventions)

Q53 Did you measure how the performance of your model varied between different populations?

- ☐ Yes, by location
- ☐ Yes, by race
- ☐ Yes, by age
- ☐ Other, please specify ____
- ☐ ⊗No

Display this question:

If Q24 = Optimization: the model outputs provide recommendations for optimal decisions, like how to best allocate resources

Q54 What was the decision variable of your optimization model?

- [ ] Vaccines
- [ ] PPE
- [ ] Ventilators
- [ ] Other, please specify ____
- [ ] Patient transfers
- [ ] Staffing
- [ ] Capacity Management
- [ ] Other, please specify ____
- [ ] Other, please specify ____

Display this question:

If Q24 = Optimization: the model outputs provide recommendations for optimal decisions, like how to best allocate resources

Q55 Which of the following factors were part of the objective function of your optimization model?

- ☐ Cost
- ☐ Health outcomes (e.g. minimizing deaths)
- ☐ Fairness
- ☐ Other, please specify ________________

Display this question:

If Q24 = Optimization: the model outputs provide recommendations for optimal decisions, like how to best allocate resources

Q56 How did your optimization model handle uncertainty?

- ☐ Stochastic optimization
- ☐ Robust optimization
- ☐ Scenario analysis
- ☐ Sensitivity analysis
- ☐ Other, please specify ________________
- ☐ ⊗We did not address uncertainty

Q57 Did you get input from relevant stakeholders or potential model users as you developed your model?

- ☐ Yes, we got input in the early stages of model development
- ☐ Yes, we got feedback once our model was operational
- ☐ No, we did not get input

Challenges

Q58 Please rate the extent to which the following challenges impacted this project.

	Not impactful	Slightly impactful	Moderately impactful	Impactful	Strongly impactful
Model performance issues					
Poor data quality					
Poor data availability					
Difficulty producing timely results					
Poor generalizability of modeling results (the transferability of findings from one specific population to another)					
Unknowable factors (cannot be known at the time of building the model, like future variants or interventions implemented)					
Insufficient funding					
Insufficient human resources with necessary skills					
Insufficient computational resources					
Stakeholder or model user priorities changing					
Limited feedback from stakeholders or model users					
Difficulty translating data/modeling work to public health practice (e.g. informing decision-makers on policymaking, resource allocation, etc.)					

Q59 Were there any other significant challenges that impacted this project?

○ Yes

○ No

Display this question:

If Q59 = Yes

Q60 Please list and rate the impact of any other significant challenges that impacted this project.

	Not impactful	Slightly impactful	Moderately impactful	Impactful	Strongly impactful
Challenge 1:	○	○	○	○	○
Challenge 2:	○	○	○	○	○
Challenge 3:	○	○	○	○	○
Challenge 4:	○	○	○	○	○
Challenge 5:	○	○	○	○	○

Q61 Is there anything else you would like to share about your experience developing models? Optional.

____

Building Datasets or Models – Methodology – Data Aggregators or Generators

This section is shown if “data aggregation” or “data generation” was selected for Q16

Continue to answer these questions based on your one selected project.

Q62 What types of data did you collect?

- ☐ COVID-19 case or death data
- ☐ COVID-19 hospitalizations data
- ☐ Other epidemiological data (e.g. influenza, SARS-CoV-1)
- ☐ Vaccines allocated or administered data
- ☐ Testing data (e.g. number of tests, positivity rate)
- ☐ Serological surveillance data
- ☐ Electronic medical records or individual-level health data
- ☐ Genomic surveillance data (e.g. variant prevalence)
- ☐ Wastewater surveillance data
- ☐ Mobility data
- ☐ Other behavioral data (e.g. survey, social media or news sentiment analysis)
- ☐ COVID-19 response policy data
- ☐ Climate or environmental data (e.g. temperature, humidity)
- ☐ Consumer data (e.g. home test, mask, and disinfectant purchases)
- ☐ Health risk factors data

- [ ] Other demographic data
- [ ] Other, please specify

____

Q63 What motivated you to generate a new data set?

- [ ] Data is not available elsewhere
- [ ] Existing data is not readily accessible
- [ ] Data available but is of poor quality, unreliable, or incomplete
- [ ] Data available but not at a high spatial resolution
- [ ] Data available but not at a high temporal resolution
- [ ] Data available generally but not for a specific population or group
- [ ] Other, please specify

____

Q64 Which of the following research objectives did your project address?

- ☐ To learn about the current situation and increase situational awareness
- ☐ To predict the near future
- ☐ To obtain virus and transmission characteristics (e.g. transmissibility, infection fatality ratio, generation time, asymptomatic proportion of cases)
- ☐ To learn about factors that impact SARS-CoV-2 transmission
- ☐ To learn about the effectiveness of vaccination
- ☐ To learn about the effectiveness of different policy interventions and/or surveillance systems
- ☐ To learn about possible long-term scenarios
- ☐ To learn which populations are most at-risk
- ☐ To inform resource management (e.g. hospital capacity, medical supplies, testing capacity, vaccine allocation)
- ☐ To communicate information about COVID-19 and/or risk reduction behaviors to the public
- ☐ Other, please specify ____

Geographic Coverage

Q65 What was your data's geographic resolution?

- ☐ National
- ☐ Regional (smaller than national, larger than state)
- ☐ State
- ☐ County
- ☐ City or metropolitan area
- ☐ Smaller than county or city level (e.g. census tract)
- ☐ Other, please specify ________________

Display this question:

If Q65 = Regional (smaller than national, larger than state)

Q66 Which regions best describe your geographic coverage?

- ☐ New England (Connecticut, Maine, Massachusetts, New Hampshire, Rhode Island, Vermont)
- ☐ Middle Atlantic (New Jersey, New York, Pennsylvania)
- ☐ East North Central (Indiana, Illinois, Michigan, Ohio, Wisconsin)
- ☐ West North Central (Iowa, Kansas, Minnesota, Missouri, Nebraska, North Dakota, South Dakota)
- ☐ South Atlantic (Delaware, DC, Florida, Georgia, Maryland, North Carolina, South Carolina, Virginia, West Virginia)
- ☐ East South Central (Alabama, Kentucky, Mississippi, Tennessee)
- ☐ West South Central (Arkansas, Louisiana, Oklahoma, Texas)
- ☐ Mountain (Arizona, Colorado, Idaho, New Mexico, Montana, Utah, Nevada, Wyoming)
- ☐ Pacific (Alaska, California, Hawaii, Oregon, Washington)
- ☐ Puerto Rico
- ☐ Guam
- ☐ US Virgin Islands
- ☐ Northern Mariana Islands
- ☐ American Samoa

☐ Other, please specify ____

Display this question:

If Q65 = State

Q67 Which option best describes your state level geographic coverage?

○ All states, including territories (Puerto Rico, Guam, American Samoa, Northern Mariana Islands, etc.)

○ All states and Puerto Rico

○ All states, no territories

○ States in certain regions of the US

○ 5 states or less

○ Other, please specify ____

Display this question:

If Q67 = States in certain regions of the US

Q68 Which regions best describe your state level geographic coverage?

- ▢ New England (Connecticut, Maine, Massachusetts, New Hampshire, Rhode Island, Vermont)
- ▢ Middle Atlantic (New Jersey, New York, Pennsylvania)
- ▢ East North Central (Indiana, Illinois, Michigan, Ohio, Wisconsin)
- ▢ West North Central (Iowa, Kansas, Minnesota, Missouri, Nebraska, North Dakota, South Dakota)
- ▢ South Atlantic (Delaware, DC, Florida, Georgia, Maryland, North Carolina, South Carolina, Virginia, West Virginia)
- ▢ East South Central (Alabama, Kentucky, Mississippi, Tennessee)
- ▢ West South Central (Arkansas, Louisiana, Oklahoma, Texas)
- ▢ Mountain (Arizona, Colorado, Idaho, New Mexico, Montana, Utah, Nevada, Wyoming)
- ▢ Pacific (Alaska, California, Hawaii, Oregon, Washington)
- ▢ Puerto Rico
- ▢ Guam
- ▢ US Virgin Islands
- ▢ Northern Mariana Islands
- ▢ American Samoa

☐ Other, please specify

____

Display this question:

If Q67 = 5 states or less

Q69 Which US states?

- ☐ Alabama
- ☐ Alaska
- ☐ Arizona
- ☐ Arkansas
- ☐ California
- ☐ Colorado
- ☐ Connecticut
- ☐ Delaware
- ☐ District of Columbia
- ☐ Florida
- ☐ Georgia
- ☐ Hawaii
- ☐ Idaho
- ☐ Illinois
- ☐ Indiana

- [ ] Iowa
- [ ] Kansas
- [ ] Kentucky
- [ ] Louisiana
- [ ] Maine
- [ ] Maryland
- [ ] Massachusetts
- [ ] Michigan
- [ ] Minnesota
- [ ] Mississippi
- [ ] Missouri
- [ ] Montana
- [ ] Nebraska
- [ ] Nevada
- [ ] New Hampshire
- [ ] New Jersey

- [ ] New Mexico
- [ ] New York
- [ ] North Carolina
- [ ] North Dakota
- [ ] Ohio
- [ ] Oklahoma
- [ ] Oregon
- [ ] Pennsylvania
- [ ] Puerto Rico
- [ ] Rhode Island
- [ ] South Carolina
- [ ] South Dakota
- [ ] Tennessee
- [ ] Texas
- [ ] Utah
- [ ] Vermont

☐ Virginia

☐ Washington

☐ West Virginia

☐ Wisconsin

☐ Wyoming

Display this question:

If Q65 = County

Q70 Which option best describes your county level geographic coverage?

○ All or most counties in the US, and equivalent resolution in the territories

○ All or most counties in the in US, excluding territories

○ All or most counties in certain regions of the US

○ All or most counties in 5 or less states

○ If less than 5 counties, please list the counties

____

○ Other, please specify ____

Display this question:

If Q70 = All or most counties in certain regions of the US

Q71 Which regions best describe your county level geographic coverage?

▢ New England (Connecticut, Maine, Massachusetts, New Hampshire, Rhode Island, Vermont)

▢ Middle Atlantic (New Jersey, New York, Pennsylvania)

▢ East North Central (Indiana, Illinois, Michigan, Ohio, Wisconsin)

▢ West North Central (Iowa, Kansas, Minnesota, Missouri, Nebraska, North Dakota, South Dakota)

▢ South Atlantic (Delaware, DC, Florida, Georgia, Maryland, North Carolina, South Carolina, Virginia, West Virginia)

▢ East South Central (Alabama, Kentucky, Mississippi, Tennessee)

▢ West South Central (Arkansas, Louisiana, Oklahoma, Texas)

▢ Mountain (Arizona, Colorado, Idaho, New Mexico, Montana, Utah, Nevada, Wyoming)

▢ Pacific (Alaska, California, Hawaii, Oregon, Washington)

▢ Puerto Rico

▢ Guam

▢ US Virgin Islands

▢ Northern Mariana Islands

▢ American Samoa

☐ Other, please specify

____

Display this question:

If Q70 = All or most counties in 5 or less states

Q72 Which US states best describe your county level coverage?

- ☐ Alabama
- ☐ Alaska
- ☐ Arizona
- ☐ Arkansas
- ☐ California
- ☐ Colorado
- ☐ Connecticut
- ☐ Delaware
- ☐ District of Columbia
- ☐ Florida
- ☐ Georgia
- ☐ Hawaii
- ☐ Idaho
- ☐ Illinois
- ☐ Indiana

- [ ] Iowa
- [ ] Kansas
- [ ] Kentucky
- [ ] Louisiana
- [ ] Maine
- [ ] Maryland
- [ ] Massachusetts
- [ ] Michigan
- [ ] Minnesota
- [ ] Mississippi
- [ ] Missouri
- [ ] Montana
- [ ] Nebraska
- [ ] Nevada
- [ ] New Hampshire
- [ ] New Jersey

- ☐ New Mexico
- ☐ New York
- ☐ North Carolina
- ☐ North Dakota
- ☐ Ohio
- ☐ Oklahoma
- ☐ Oregon
- ☐ Pennsylvania
- ☐ Puerto Rico
- ☐ Rhode Island
- ☐ South Carolina
- ☐ South Dakota
- ☐ Tennessee
- ☐ Texas
- ☐ Utah
- ☐ Vermont

- ☐ Virginia
- ☐ Washington
- ☐ West Virginia
- ☐ Wisconsin
- ☐ Wyoming

Display this question:

If Q65 = Smaller than county or city level (e.g. census tract)

Or Q65 = City or metropolitan area

Q73 Please briefly describe the geographic resolution and coverage of your data (e.g. census tract level in Baltimore city)

____

Methods

Q74 Did your data collection focus on any of the following populations?

- ☐ Prisons
- ☐ Schools, excluding universities
- ☐ Universities
- ☐ Nursing homes
- ☐ Medical facilities
- ☐ Frontline workers (medical personnel)
- ☐ Essential workers (non-medical personnel)
- ☐ Other, please specify ____
- ☐ ⊗None of the above

Q75 Did you provide data that were broken down by any of these categories?

- ☐ Race or ethnicity
- ☐ Age
- ☐ Sex
- ☐ Other, please specify
- ☐ ⊗None of the above

Q76 Did you collect data with values that varied over time?

- ○ Yes
- ○ No

Display this question:

If Q76 = Yes

Q77 During the peak operation of your data collection effort, what was the reporting frequency of your data?

- ☐ Daily or more frequent
- ☐ Twice a week
- ☐ Weekly
- ☐ Twice a month
- ☐ Monthly
- ☐ Less frequent than monthly
- ☐ Other, please specify ________________

Q78 During the peak operation of your data collection effort, how long was the lag between when the data was current and when it was made available?

- ☐ Less than 1 day
- ☐ 1-3 days
- ☐ 4-7 days
- ☐ Between 1-2 weeks
- ☐ Between 2 weeks and 1 month
- ☐ 1-3 months
- ☐ More than 3 months
- ☐ Other, please specify

____

Challenges

Display this question:

If Q17 = Data Aggregation: collecting and centralizing existing data for ease of access (e.g. JHU CSSE, Delphi Epidata API)

Q79 Please rate the extent to which the following challenges impacted your work.

	Not impactful	Slightly impactful	Moderately impactful	Impactful	Strongly impactful
Obtaining a complete data set (no missing values)	○	○	○	○	○
Anonymizing the data	○	○	○	○	○
Adopting standard definitions of variables of interest	○	○	○	○	○
Maintaining a timely and regular reporting schedule	○	○	○	○	○
Insufficient funding	○	○	○	○	○
Insufficient human resources with necessary skills	○	○	○	○	○
Insufficient computational resources	○	○	○	○	○
Adapting to the changing needs of data users	○	○	○	○	○
Difficulty translating data/modeling work to public health practice (e.g. informing decision-makers on policy making, resource allocation, etc.)	○	○	○	○	○

Display this question:

If Q17 = Data Aggregation: collecting and centralizing existing data for ease of access (e.g. JHU CSSE, Delphi Epidata API)

Q80 Please rate to what extent the following limitations of the primary data impacted your work. Primary data refers to datasets that you clean, aggregate, curate, etc. to generate your final dataset.

	Not impactful	Slightly impactful	Moderately impactful	Impactful	Strongly impactful
Anomalies in data	○	○	○	○	○
Incompleteness of data (missing values)	○	○	○	○	○
Reporting frequency irregularities	○	○	○	○	○
Data accessibility: not publicly available	○	○	○	○	○
Data accessibility: not in machine readable format	○	○	○	○	○
Data accessibility: cost prohibitive (data available but too expensive)	○	○	○	○	○
Data accessibility: privacy or data use agreement issues	○	○	○	○	○
Lack of standardization in data definitions (e.g. across jurisdictions, over time)	○	○	○	○	○
Lack of documentation from data generators on how data was collected, anomalies in the data, etc.	○	○	○	○	○

Display this question:

If Q17 = Data Generation: generating data that was not previously available (e.g. Facebook COVID-19 Trends and Impact Survey, sequencing to produce genomic data, conducting wastewater surveillance)

Q81 Please rate to what extent the following challenges impacted your work.

	Not impactful	Slightly impactful	Moderately impactful	Impactful	Strongly impactful
Obtaining an acceptable sample size	○	○	○	○	○
Obtaining a sample that was representative of the population of interest	○	○	○	○	○
Obtaining a complete data set (no missing values)	○	○	○	○	○
Anonymizing the data	○	○	○	○	○
Adopting standard definitions of variables of interest	○	○	○	○	○
Maintaining a timely and regular reporting schedule	○	○	○	○	○
Insufficient funding	○	○	○	○	○
Insufficient human resources with necessary skills	○	○	○	○	○
Insufficient computational resources	○	○	○	○	○
Adapting to the changing needs of data users	○	○	○	○	○
Difficulty translating data/modeling work to public health practice (e.g. informing decision-makers on policymaking, resource allocation, etc.)	○	○	○	○	○

Q82 Were there any other significant challenges that impacted this project?

◯ Yes

◯ No

Display this question:

If Q82 = Yes

Q83 Please list and rate the impact of any other significant challenges that impacted this project

	Not impactful	Slightly impactful	Moderately impactful	Impactful	Strongly impactful
Challenge 1:	◯	◯	◯	◯	◯
Challenge 2:	◯	◯	◯	◯	◯
Challenge 3:	◯	◯	◯	◯	◯
Challenge 4:	◯	◯	◯	◯	◯
Challenge 5:	◯	◯	◯	◯	◯

Q84 Did your data have anomalies?

◯ Yes

◯ No

Display this question:

If Q84 = Yes

Q85 Did you provide corrections for these anomalies to data users?

- ◯ Yes
- ◯ No

Q86 Were any of the following sampling biases present in your dataset?

- ▢ Sampling was not geographically representative
- ▢ Different groups were over or under represented
- ▢ Sampling was not consistent over time
- ▢ Too small of a sample size
- ▢ Other, please specify ____
- ▢ ⊗None of the above

Display this question:
If Q86 != None of the above

Q87 Did you provide corrections for sampling bias to data users?

- ◯ Yes
- ◯ No

Q88 Did you get input from data users as you developed your data collection process?

☐ Yes, we got input in the early stages of our data collection effort

☐ Yes, we solicited feedback once our data was available

☐ ⊗No, we did not get input

Q89 Was your data shared publicly?

○ Yes

○ No

Q90 Is there any other information you'd like to share about your experience collecting data? Optional.

____

____

Building Datasets or Models – Translation

All tool builders (selected "building models, tools, and/or data sets" for Q13) see this section

Continue to answer these questions based on your one selected project.

Q91 How were your findings shared?

- [ ] Peer-reviewed journal
- [ ] Pre-print
- [ ] Report
- [ ] News or social media (including if your team was not involved in the sharing or coverage)

Display this choice:
If Q17 = Modeling or other forms of data analysis

- [ ] US Forecast Hub

Display this choice:
If Q17 = Modeling or other forms of data analysis

- [ ] US Scenario Modeling Hub
- [ ] Findings were shared within my institution
- [ ] Direct communication with stakeholders (e.g. public health institutions, other government officials, healthcare institutions)
- [ ] Other, please specify ____
- [ ] None of the above

Display this question:

If Q91 = Peer-reviewed journal

Or Q91 = Pre-print

Or Q91 = Report

Q92 Please share the links of any publications, preprints, or reports, if available.

____

____

News or Social Media

Display this question:

If Q91 = News or social media (including if your team was not involved in the sharing or coverage)

Q93 Which types of media were your findings shared on?

- ▢ COVID-19 digital dashboards, maps, and other visualization tools
- ▢ Print media, including their online versions (e.g. newspapers, magazines)
- ▢ Broadcast media (e.g. TV, radio)
- ▢ Podcasts
- ▢ Social media (e.g. Twitter, Instagram, YouTube)
- ▢ Other online sources (e.g. blogs)
- ▢ Other, please specify ____

Display this question:

If Q91 = News or social media (including if your team was not involved in the sharing or coverage)

Q94 When your findings were covered, how often did your team communicate directly with the media personnel covering your findings?

- ○ Never
- ○ Sometimes
- ○ About half of the time
- ○ Usually
- ○ Always
- ○ Not applicable, our findings were not covered by the media

---

Display this question:

If Q94 != Not applicable, our findings were not covered by the media

And Q91 = News or social media (including if your team was not involved in the sharing or coverage)

Q95 How much control did your team usually have over the resulting coverage?

- ▢ Complete control (i.e. an article written by our team was published with no significant modification)
- ▢ Some control (i.e. we did not write the article, but we were consulted or interviewed for the piece and were given an opportunity to correct it before publication)
- ▢ Little control (i.e. our team was consulted or interviewed for the piece, but had no other involvement)
- ▢ None (i.e. the media covered our findings without any communication with our team)

Display this question:

If Q94 != Not applicable, our findings were not covered by the media

And Q91 = News or social media (including if your team was not involved in the sharing or coverage)

Q96 Please rate to what extent you agree or disagree with the following statements about media covering your findings.

	Strongly disagree	Moderately disagree	Neither agree nor disagree	Moderately agree	Strongly agree
The media portrayed an accurate representation of my findings.	○	○	○	○	○
My work was presented in a way that served political purposes.	○	○	○	○	○

Display this question:

If Q91 = News or social media (including if your team was not involved in the sharing or coverage)

Q97 Is there anything else you want to share about media coverage of your findings? Optional.

____

____

Findings Shared Within Your Institution

Display this question:

If Q91 = Findings were shared within my institution

Q98 Did your team interact with relevant decision-makers? For PHIs, relevant decision-makers would be policymakers in your jurisdiction. For healthcare institutions, relevant decision-makers would be institutional leadership that can take action based on your work or recommendations.

- ☐ My team directly interacted with decision-makers
- ☐ The leadership of my institution interacted with decision-makers
- ☐ My institution did not interact with decision-makers
- ☐ Other, please specify
- ☐ I don't know

Display this question:

If Q91 = Findings were shared within my institution

Q99 Please rate the extent to which you agree with the following statements about your work's influence on relevant decision makers.

	I don't know	Strongly disagree	Disagree	Neither agree nor disagree	Agree	Strongly Agree
Decision-makers took into account our work and/or recommendations.	○	○	○	○	○	○
Decision-makers made decisions based on data, research, and/or public health knowledge when possible.	○	○	○	○	○	○
Politics (national, local, or institutional) mainly dictated decision-making.	○	○	○	○	○	○

Display this question:

If Q8 = Response

And Q91 != Findings were shared within my institution

Q100 Why weren't your findings shared within your institution?

- ☐ I did not think my work was relevant for these types of stakeholders.
- ☐ Communicating with these stakeholders was not part of my focus or goals.
- ☐ I did not have enough time or capacity to communicate with these stakeholders.
- ☐ I did not know how to get in contact with one of these stakeholders.
- ☐ I tried to communicate with one or more of these stakeholders but was unsuccessful.
- ☐ Other, please specify ____

Display this question:

If Q91 = Findings were shared within my institution

Or Q8 = Response

And Q91 != Findings were shared within my institution

Q101 Is there anything else you want to share about sharing findings within your institution? Optional.

____

Communication with Stakeholders

Display this question:

If Q91 = Direct communication with stakeholders (e.g. public health institutions, other government officials, healthcare institutions)

Q102 What stakeholders did you communicate with about your findings?

- ▢ Public health institutions (e.g. CDC or state, local, tribal health departments)
- ▢ Other government officials (e.g. governors, mayors, congress members, judicial branch personnel)
- ▢ Healthcare institutions (e.g. hospital networks, nursing homes)
- ▢ Other, please specify ____

Display this question:

If Q102 != Public health institutions (e.g. CDC or state, local, tribal health departments)

And Q102 != Other government officials (e.g. governors, mayors, congress members, judicial branch personnel)

And Q102 != Healthcare institutions (e.g. hospital networks, nursing homes)

And Q8 = Research

Q103 Why didn't you communicate with any public health institutions, healthcare institutions, or government officials?

- [ ] I did not think my work was relevant for these types of stakeholders.
- [ ] Communicating with these stakeholders was not part of my focus or goals.
- [ ] I did not have enough time or capacity to communicate with these stakeholders.
- [ ] I did not know how to get in contact with one of these stakeholders.
- [ ] I tried to communicate with one or more of these stakeholders but was unsuccessful.
- [ ] Other, please specify ____

Display this question:

If Q8 = Research

And Q91 != Direct communication with stakeholders (e.g. public health institutions, other government officials, healthcare institutions)

Q104 Please rate the extent to which the following challenges impacted your efforts to collaborate with stakeholders in general (not specific to the selected project).

	Not applicable	Not impactful	Slightly impactful	Moderately impactful	Impactful	Strongly impactful
Difficulty finding and getting in contact with relevant stakeholders	○	○	○	○	○	○
Difficulty with data sharing or establishing data use agreements	○	○	○	○	○	○
Lack of expertise on effective communication with stakeholders	○	○	○	○	○	○
Lack of data or information available to answer stakeholder questions	○	○	○	○	○	○
Insufficient funding	○	○	○	○	○	○
Logistical or scheduling challenges	○	○	○	○	○	○
Insufficient time with stakeholders to adequately explain our work	○	○	○	○	○	○
Limited time or capacity for collaborations with stakeholders due to competing priorities, overstretched human resources, etc.	○	○	○	○	○	○
Adapting to changing stakeholder needs	○	○	○	○	○	○

Limited feedback from stakeholders or data/model users	○	○	○	○	○	○
Difficulty translating data or modeling work to public health practice (e.g. informing decision-makers on policymaking, resource allocation, etc.)	○	○	○	○	○	○

Display this question:

If Q8 = Research

And Q91 != Direct communication with stakeholders (e.g. public health institutions, other government officials, healthcare institutions)

Q105 Were there any other significant challenges that impacted your efforts to collaborate with stakeholders in general (not specific to the selected project).

○ Yes

○ No

Display this question:

If Q105 = Yes

Q106 Please list and rate the impact of any other significant challenges that impacted your efforts to collaborate with stakeholders in general (not specific to the selected project).

	Not impactful	Slightly impactful	Moderately impactful	Impactful	Strongly impactful
Challenge 1:	○	○	○	○	○
Challenge 2:	○	○	○	○	○
Challenge 3:	○	○	○	○	○
Challenge 4:	○	○	○	○	○
Challenge 5:	○	○	○	○	○

Display this question:

If Q8 = Research

And Q91 != Direct communication with stakeholders (e.g. public health institutions, other government officials, healthcare institutions)

Q107 Is there anything else you want to share about collaborations with stakeholders? Optional.

____

Display this question:

If Q91 = Direct communication with stakeholders (e.g. public health institutions, other government officials, healthcare institutions)

Please select one collaboration with a stakeholder to answer further questions on. We recommend you select the most impactful collaboration.

Display this question:

If Q91 = Direct communication with stakeholders (e.g. public health institutions, other government officials, healthcare institutions)

Q108 What type of stakeholder is your selected collaboration with?
Only show answer choices that were selected in Q102

○ Public health institutions (e.g. CDC or state, local, tribal health departments)

○ Other government officials (e.g. governors, mayors, congress members, judicial branch personnel)

○ Healthcare institutions (e.g. hospital networks, nursing homes)

○ Other, please specify ____

Display this question:

If Q91 = Direct communication with stakeholders (e.g. public health institutions, other government officials, healthcare institutions)

And Q108 = Public health institutions (e.g. CDC or state, local, tribal health departments) Is Selected

Q109 Which of the following public health institutions did you communicate with for the selected collaboration?

▢ CDC

▢ State health department

▢ County health department

▢ City or metropolitan health department

▢ Tribal health department

▢ Other, please specify ____

Display this question:

If Q91 = Direct communication with stakeholders (e.g. public health institutions, other government officials, healthcare institutions)

And Q108 = Other government officials (e.g. governors, mayors, congress members, judicial branch personnel) Is Selected

Q110 Which of the following government offices or officials did you communicate with for the selected collaboration?

- ☐ Mayor’s office or equivalent
- ☐ City council members or equivalent
- ☐ Local or municipal courts
- ☐ Other, please specify ________________

- ☐ Governor’s office
- ☐ State congress members
- ☐ State courts
- ☐ Other, please specify ________________

- ☐ President’s office
- ☐ US Center for Disease Control
- ☐ US Department of Health and Human Services
- ☐ US Congress

- [ ] Federal courts
- [ ] Other, please specify

____

- [ ] Tribal jurisdiction
- [ ] Other, please specify

____

Display this question:

If Q91 = Direct communication with stakeholders (e.g. public health institutions, other government officials, healthcare institutions)

And Q108 = Healthcare institutions (e.g. hospital networks, nursing homes) Is Selected

Q111 Which of the following healthcare institutions did you communicate with for the selected collaboration?

- [ ] Hospital
- [ ] Health clinic or outpatient care facility
- [ ] Nursing home
- [ ] Rehabilitation facility
- [ ] Other institution devoted to the care of sick, infirm, or aged person
- [ ] Other, please specify

____

Display this question:

If Q91 = Direct communication with stakeholders (e.g. public health institutions, other government officials, healthcare institutions)

Q112 Before the COVID-19 pandemic, had you communicated or worked with this stakeholder before?

- ◯ Yes, I had been in contact with this stakeholder before
- ◯ Yes, I had worked with this stakeholder before
- ◯ No, I had not communicated with this stakeholder before the COVID-19 pandemic

Display this question:

If Q91 = Direct communication with stakeholders (e.g. public health institutions, other government officials, healthcare institutions)

Q113 Did this stakeholder provide access to data that was not publicly available?

- ◯ Yes
- ◯ No

Display this question:

If Q91 = Direct communication with stakeholders (e.g. public health institutions, other government officials, healthcare institutions)

Q114 Which of the following tools did you provide to this stakeholder?

- ▢ Access to data that was not publicly available
- ▢ Modeling tools or expertise
- ▢ Access to expert advice and/or policy recommendations
- ▢ Synthesis of current COVID-19 information
- ▢ Other, please specify ____

Display this question:

If Q91 = Direct communication with stakeholders (e.g. public health institutions, other government officials, healthcare institutions)

Q115 How frequently did you communicate with this stakeholder during the peak of your collaboration?

- ○ More than 10 times
- ○ 5-10 times
- ○ 3-5 times
- ○ 1-2 times
- ○ Every 2 weeks or more often
- ○ Monthly
- ○ Every 2 months
- ○ Quarterly or less often

Display this question:

If Q91 = Direct communication with stakeholders (e.g. public health institutions, other government officials, healthcare institutions)

And Q17 = Modeling or other forms of data analysis

Q116 How much influence did stakeholder needs have on the development of your model?

- ▢ We developed a model from scratch to meet stakeholder needs
- ▢ We customized an existing model to meet stakeholder needs
- ▢ We did not adopt our model to stakeholder needs

Display this question:

If Q91 = Direct communication with stakeholders (e.g. public health institutions, other government officials, healthcare institutions)

And Q17 = Modeling or other forms of data analysis

Q117 During which stages of model development did you get stakeholder feedback?

- ▢ In the early stages of model development
- ▢ Once our model was operational
- ▢ ⊗We did not get feedback from this stakeholder

Display this question:

If Q91 = Direct communication with stakeholders (e.g. public health institutions, other government officials, healthcare institutions)

and

If Q17 = Data Aggregation: collecting and centralizing existing data for ease of access (e.g. JHU CSSE, Delphi Epidata API)

Or Q17 = Data Generation: generating data that was not previously available (e.g. Facebook COVID-19 Trends and Impact Survey, sequencing to produce genomic data, conducting wastewater surveillance)

Q118 How much influence did stakeholder needs have on the development of your data collection process or analysis?

- ☐ We developed a data collection process or analysis from scratch to meet stakeholder needs

- ☐ We customized an existing data collection process or analysis to meet stakeholder needs

- ☐ We did not adopt our data collection process or analysis to stakeholder needs

---

Display this question:

If Q91 = Direct communication with stakeholders (e.g. public health institutions, other government officials, healthcare institutions)

and

If Q17 = Data Aggregation: collecting and centralizing existing data for ease of access (e.g. JHU CSSE, Delphi Epidata API)

Or Q17 = Data Generation: generating data that was not previously available (e.g. Facebook COVID-19 Trends and Impact Survey, sequencing to produce genomic data, conducting wastewater surveillance)

Q119 During which stages of data collection or analysis did you get stakeholder feedback?

- [ ] In the early stages of data collection or analysis
- [ ] Once our data or its analysis was available
- [ ] ⊗We did not get feedback from this stakeholder

Display this question:

If Q91 = Direct communication with stakeholders (e.g. public health institutions, other government officials, healthcare institutions)

Q120 Rate the extent to which the following challenges impacted your collaboration with this stakeholder.

	Not impactful	Slightly impactful	Moderately impactful	Impactful	Strongly impactful
Difficulty with data sharing or establishing data use agreements	○	○	○	○	○
Lack of expertise on effective communication with stakeholders	○	○	○	○	○
Lack of data or information available to answer stakeholder questions	○	○	○	○	○
Insufficient funding	○	○	○	○	○
Logistical or scheduling challenges	○	○	○	○	○
Insufficient time with stakeholders to adequately explain our work	○	○	○	○	○
Limited time or capacity for collaborations with stakeholders due to competing priorities, overstretched human resources, etc.	○	○	○	○	○
Adapting to changing stakeholder needs	○	○	○	○	○
Difficulty producing results fast enough for decision-making timelines	○	○	○	○	○
Limited feedback from stakeholders or data/model users	○	○	○	○	○
Difficulty translating data/modeling work to public health practice (e.g. informing decision-makers on policymaking, resource allocation, etc.)	○	○	○	○	○

Display this question:

If Q91 = Direct communication with stakeholders (e.g. public health institutions, other government officials, healthcare institutions)

Q121 Were there any other significant challenges that impacted your collaboration with this stakeholder?

○ Yes

○ No

Display this question:

If Q91 = Direct communication with stakeholders (e.g. public health institutions, other government officials, healthcare institutions)

And Q121 = Yes

Q122 Please list and rate the impact of any other significant challenges that impacted your collaboration with this stakeholder.

	Not impactful	Slightly impactful	Moderately impactful	Impactful	Strongly impactful
Challenge 1:	○	○	○	○	○
Challenge 2:	○	○	○	○	○
Challenge 3:	○	○	○	○	○
Challenge 4:	○	○	○	○	○
Challenge 5:	○	○	○	○	○

If Q91 = Direct communication with stakeholders (e.g. public health institutions, other government officials, healthcare institutions)

Q123 To what extent do you agree or disagree with the following statements about your experiences engaging with this stakeholder?

	Strongly disagree	Moderately disagree	Neither agree nor disagree	Moderately agree	Strongly agree
Stakeholders clearly communicated their needs.	○	○	○	○	○
Stakeholders were able to understand our findings and appropriate interpretations.	○	○	○	○	○
Stakeholders were able to understand the uncertainty and limitations of our findings.	○	○	○	○	○
We built a positive working relationship with this stakeholder that will continue into the future.	○	○	○	○	○

If Q91 = Direct communication with stakeholders (e.g. public health institutions, other government officials, healthcare institutions)

Q124 To what extent did your engagement with this stakeholder inform their public health work (e.g. decision-making, policy, resource allocation, situational awareness)?

○ Stakeholders informed us how our findings were used to inform their work

○ We got positive feedback on the usefulness of our work, but we are not sure if or how it was used

○ We did not receive feedback on whether our work was useful

If Q91 = Direct communication with stakeholders (e.g. public health institutions, other government officials, healthcare institutions)

Q125 Is there anything else you want to share about collaborations with stakeholders? Optional.

____

Communication with Researchers

This section is shown to respondents that indicated that their institution primarily does response work, rather than research (selected “response” for Q8)

If Q8 = Response

Q126 Did you collaborate with any researchers outside of your institution?   By collaborate, we mean having meetings with researchers to discuss COVID-19, for which researchers may have presented data and/or models or gave advice

- ○ Yes
- ○ No

Display this question:

If Q8 = Response

And Q126 = No

Q127 Why didn’t you collaborate with researchers outside of your institution?

- ☐ I didn't think researchers' work was relevant for my COVID-19 response work.
- ☐ Communicating with researchers was not part of my focus or goals.
- ☐ I did not have enough time or capacity to communicate with researchers.
- ☐ I did not know how to get in contact with researchers.
- ☐ I tried to communicate with researchers but was unsuccessful.
- ☐ Other, please specify ____

Display this question:

If Q8 = Response

And Q126 = No

Q128 Please rate how impactful the following challenges were for establishing successful collaborations with researchers in general (no longer specific to the selected project).

	Not applicable	Not impactful	Slightly impactful	Moderately impactful	Impactful	Strongly impactful
Difficulty finding and getting in contact with relevant researchers	○	○	○	○	○	○
Difficulty with data sharing or establishing data use agreements	○	○	○	○	○	○
Lack of data or information available to answer our questions	○	○	○	○	○	○
Insufficient funding	○	○	○	○	○	○
Logistical or scheduling challenges	○	○	○	○	○	○
Insufficient time with researchers for them to adequately explain their work	○	○	○	○	○	○
Limited time or capacity for collaborations with researchers due to competing priorities, overstretched human resources, etc.	○	○	○	○	○	○
Researchers struggling to adapt to our changing needs	○	○	○	○	○	○
Researchers unable to produce results fast enough for decision-making timelines	○	○	○	○	○	○
Limited opportunities to provide feedback to researchers	○	○	○	○	○	○

Difficulty translating data or modeling work to public health practice (e.g. informing decision-makers on policymaking, resource allocation, etc.)	◯	◯	◯	◯	◯	◯

Display this question:

If Q8 = Response

And Q126 = No

Q129 Were there any other significant challenges that impacted your efforts to establish collaborations with researchers?

◯ Yes

◯ No

Display this question:

If Q8 = Response

And Q129 = Yes

Q130 Please list and rate the impact of any other significant challenges that impacted your efforts to establish collaborations with researchers.

	Not impactful	Slightly impactful	Moderately impactful	Impactful	Strongly impactful
Challenge 1:	○	○	○	○	○
Challenge 2:	○	○	○	○	○
Challenge 3:	○	○	○	○	○
Challenge 4:	○	○	○	○	○
Challenge 5:	○	○	○	○	○

Display this question:

If Q8 = Response

And Q126 = Yes

Q131 Did you collaborate with more than one group of researchers?

○ Yes

○ No

Display this question:

If Q8 = Response

And Q131 = Yes

Q203 Please select one collaboration to report on. We recommend that you select the collaboration that was most useful for your work.

Display this question:

If Q8 = Response

And Q126 = Yes

Q132 Before the COVID-19 pandemic, had you communicated or worked with these researchers before?

- ○ Yes, I had been in contact with these researchers before
- ○ Yes, I had worked with these researchers before
- ○ No, I had not communicated with these researchers before the COVID-19 pandemic

Display this question:

If Q8 = Response

And Q126 = Yes

Q133 Was this collaboration funded?

- ▢ Yes, by my institution
- ▢ Yes, by the researchers or their institution
- ▢ Yes, by grant or award specific to the project
- ▢ No
- ▢ I don’t know

Display this question:

If Q8 = Response

And Q126 = Yes

Q134 Did you provide researchers with access to data that was not publicly available?

○ Yes

○ No

Display this question:

If Q8 = Response

And Q126 = Yes

Q135 Which of the following tools did researchers provide in this collaboration?

☐ Access to data that was not publicly available

☐ Modeling tools or expertise

☐ Access to expert advice or policy recommendations

☐ Synthesis of current COVID-19 data or information

☐ Other, please specify ____

Display this question:

If Q8 = Response

And Q126 = Yes

Q136 How frequently did you communicate with these researchers during the peak of your collaboration?

- ◯ More than 10 times
- ◯ 6-10 times
- ◯ 3-5 times
- ◯ 1-2 times
- ◯ Every 2 weeks or more often
- ◯ Monthly
- ◯ Every 2 months
- ◯ Quarterly or less often

Display this question:

If Q8 = Response

And Q135 = Modeling tools or expertise

Q137 How much influence did your needs have on the models discussed?

- ▢ Researchers developed a model from scratch to meet our needs
- ▢ Researchers customized an existing model to meet our needs
- ▢ Researchers did not adopt the model to our needs
- ▢ Other, please specify

____

Display this question:

If Q8 = Response

And Q135 = Modeling tools or expertise

Q138 During which stages of model development did researchers ask for feedback?

☐ In the early stages of model development

☐ Once the model was operational

☐ Researchers did not ask for feedback

☐ Other, please specify

____

Display this question:

If Q8 = Response

and

If Q135 = Access to data that was not publicly available

Or Q135 = Synthesis of current COVID-19 data or information

Q139 How much influence did your needs have on the data discussed?

☐ Researchers developed a data analysis or data collection process from scratch to meet our needs

☐ Researchers customized an existing data analysis or data collection process or to meet our needs

☐ Researchers did not adopt the data analysis or data collection process to our needs

☐ Other, please specify

____

Display this question:

If Q8 = Response

And

If Q135 = Access to data that was not publicly available

Or Q135 = Synthesis of current COVID-19 data or information

Q140 During which stages of data analysis or data collection process development did researchers ask for feedback?

- ☐ In the early stages of data collection or analysis
- ☐ Once the data or its analysis was available
- ☐ Researchers did not ask for feedback
- ☐ Other, please specify

Display this question:

If Q8 = Response

And Q126 = Yes

Q141 Please rate the extent to which the following challenges impacted your collaboration.

	Not impactful	Slightly impactful	Moderately impactful	Impactful	Strongly impactful
Difficulty with data sharing or establishing data use agreements	○	○	○	○	○
Lack of data or information available to answer our questions	○	○	○	○	○
Insufficient funding	○	○	○	○	○
Logistical or scheduling challenges	○	○	○	○	○
Insufficient time with researchers for them to adequately explain their work	○	○	○	○	○
Limited time or capacity for collaborations with researchers due to competing priorities, overstretched human resources, etc.	○	○	○	○	○
Researchers struggling to adapt to our changing needs	○	○	○	○	○
Researchers unable to produce results fast enough for decision-making timelines	○	○	○	○	○
Limited opportunities to provide feedback to researchers	○	○	○	○	○
Difficulty translating data/modeling work to public health practice (e.g. informing decision-makers on policymaking, resource allocation, etc.)	○	○	○	○	○

Display this question:

If Q8 = Response

And Q126 = Yes

Q142 Were there any other significant challenges that impacted your collaboration with researchers?

◯ Yes

◯ No

Display this question:

If Q8 = Response

And Q142 = Yes

Q143 Please list and rate the impact of any other significant challenges that impacted your collaboration.

	Not impactful	Slightly impactful	Moderately impactful	Impactful	Strongly impactful
Challenge 1:	◯	◯	◯	◯	◯
Challenge 2:	◯	◯	◯	◯	◯
Challenge 3:	◯	◯	◯	◯	◯
Challenge 4:	◯	◯	◯	◯	◯
Challenge 5:	◯	◯	◯	◯	◯

Display this question:

If Q8 = Response

And Q126 = Yes

Q144 To what extent do you agree or disagree with the following statements about your experiences engaging with researchers?

	Strongly disagree	Disagree	Neither agree nor disagree	Agree	Strongly agree
Researchers communicated the uncertainty and limitations of their data or modeling results.	○	○	○	○	○
After communicating with researchers, I was still confused about the data or modeling results and appropriate interpretations.	○	○	○	○	○
I built a positive working relationship with researchers that will continue into the future.	○	○	○	○	○

Display this question:

If Q8 = Response

And Q126 = Yes

Q145 To what extent did your engagement with researchers inform your public health work (e.g. decision-making, policy, resource allocation, situational awareness)?

- ☐ What we learned from researchers directly impacted our decision-making
- ☐ What we learned from researchers informed our situational awareness
- ☐ What we learned from researchers did not impact our public health work
- ☐ Other, please specify ____

Display this question:

If Q8 = Response

Q146 Is there anything else you want to share about collaborations with researchers? Optional.

____

Building Datasets and Models – Concluding Questions

All tool builders (selected “building models, tools, and/or data sets” for Q13) see this section

Q147 That you know of, were your findings from your selected project used for the following types of COVID-19 decision-making?

- ☐ ⊗Not that I am aware of
- ☐ Vaccination policies
- ☐ Resource ordering or allocation
- ☐ Healthcare institution operational decisions (e.g. hospital capacity management)
- ☐ Implementation of non-pharmaceutical interventions (e.g. mask mandates, stay at home orders, capacity limits, testing requirements)
- ☐ Relaxing of non-pharmaceutical interventions (e.g. mask mandates, stay at home orders, capacity limits, testing requirements)
- ☐ School closures or reopening
- ☐ Investing in public education campaigns
- ☐ Investing in contact tracing
- ☐ Investing in testing capacity
- ☐ Other, please specify ____

Display this question:

If Q147 = Not that I am aware of

Q148 Why do you think your findings were not used for decision-making?

- [ ] This work was not relevant for guiding decision-makers.
- [ ] Decision-makers were not aware of my findings.
- [ ] Decision-makers were not able to adequately interpret my findings for their purposes.
- [ ] Decision-makers were aware of and understood my findings but chose not to use them to guide decision-making.
- [ ] I think it's likely that my findings were used to inform decision-making, I'm just not aware of it.
- [ ] Other, please specify ____

Q149 Is there anything else you want to share about why your findings may or may not have been used to make COVID-19-related decisions? Optional.

____

____

Q150 Is this project ongoing?

- ○ Yes
- ○ No

Display this question:

If Q150 = No

Q151 Why did this project end?

- ☐ Ran out of funding
- ☐ We did not think the work remained relevant
- ☐ The research question was answered
- ☐ Burn-out from COVID-19 work
- ☐ Moved on to other, higher priority COVID-19 related projects
- ☐ Moved on to other, higher priority non-COVID-19 related projects
- ☐ Other, please specify

____

Q152 Please select the most impactful challenges in COVID-19 data and modeling work in general (no longer specific to your selected project). Maximum of 5 total selections.

- ☐ Insufficient funding
- ☐ Insufficient human resources with necessary skills
- ☐ Insufficient computational resources
- ☐ Desired data was never collected
- ☐ Not publicly available
- ☐ Not in machine readable format
- ☐ Cost prohibitive (data available but too expensive)
- ☐ Privacy or data use agreement issues
- ☐ Data is not timely (too much lag between when data is current and when it's made available)
- ☐ Sampling biases
- ☐ Lack of standardization in data definitions (e.g. across jurisdictions, over time)
- ☐ Other data quality issues (e.g. anomalies, missing values, irregular reporting schedules)
- ☐ Lack of direct communication with stakeholders
- ☐ Lack of expertise on effective communication with stakeholders

- [ ] Difficulty producing results fast enough for decision-making timelines

- [ ] Difficulty translating data/modeling work to public health practice (e.g. informing decision-makers on policymaking, resource allocation, etc.)

- [ ] Other, please specify ____________________

Q153 Please select your top priority areas to invest in for informing future epidemic response (maximum of 7 total selections).
Investment in data could translate to higher quality data (e.g. COVID-19 cases or deaths data), more widespread collection of this data (e.g. genomic surveillance), or new data sources being available (e.g. data that captures COVID-19 risk reduction behaviors)

- ☐ Better understanding of the current situation or situational awareness
- ☐ Predictions for the near future
- ☐ Determining factors that impact transmission or severity of disease
- ☐ Determining the effectiveness of different interventions (e.g. policies, vaccinations) or surveillance systems
- ☐ Insight on potential longer-term scenarios
- ☐ Optimal management or allocation of resources
- ☐ Other, please specify ____
- ☐ Cases or deaths data
- ☐ Hospitalizations data
- ☐ Other epidemiological data (e.g. influenza, SARS-CoV-1)
- ☐ Vaccines allocated or administered
- ☐ Testing data (e.g. number of tests, test positivity rate)
- ☐ Serological surveillance

- ☐ Electronic medical records or individual-level health data
- ☐ Genomic surveillance (e.g. variant prevalence)
- ☐ Wastewater surveillance
- ☐ Mobility data
- ☐ Other behavioral data (e.g. survey, social media or news sentiment analysis)
- ☐ Policy data
- ☐ Climate or environmental data (e.g. temperature, humidity)
- ☐ Consumer data (e.g. at-home test, mask, or disinfectant purchases)
- ☐ Health risk factors data
- ☐ Other demographic data
- ☐ Other, please specify ____
- ☐ Other, please specify ____

Q154 If you have any feedback you would like to provide on the survey, please enter it here. Optional

____

____

End of survey for tool builders

Using Datasets or Models – Introduction

The “Using Datasets or Models” sections of the survey are shown to respondents that selected “Using models, tools, and/or datasets” for Q13

This survey will consist of the following sections: 1) A brief introduction 2) Use of data 3) Use of models 4) Collaborations with researchers 5) A brief conclusion Time spent on each section will depend on whether you were involved in this type of COVID-19 response work. For example, if you were not involved in using models or a collaboration with researchers, expect the corresponding sections to be brief.

Q155 In one or two sentences, how would you describe your role or responsibilities related to COVID-19 response? How were you involved in COVID-19 decision-making?

____

____

Q156 If there is any public documentation of your COVID-19 response work (e.g. preprint, publication, report, dashboard), please enter the link here.

____

Q157 Please select the time periods in which you were involved in COVID-19 response work.

▢ Early in the COVID-19 pandemic (January-April 2020)

▢ During the middle or control phase of the COVID-19 pandemic (approximately late 2020 - mid 2021, when non-pharmaceutical interventions, like capacity limits on indoor gatherings, and vaccinations, were used to keep the pandemic under control)

▢ ⊗None of the above

Display this question:

If Q157 = Early in the COVID-19 pandemic (January-April 2020)

Q158 For your role, please rate the importance of these research objectives early in the COVID-19 pandemic (January-April 2020)

	Not at all important	A little important	Somewhat important	Very important	Extremely important
To learn about the current situation and increase situational awareness	○	○	○	○	○
To predict the near future	○	○	○	○	○
To learn about factors that impact SARS-CoV-2 transmission	○	○	○	○	○
To learn about the effectiveness of different policy interventions and/or surveillance systems	○	○	○	○	○
To learn about possible long-term scenarios	○	○	○	○	○
To learn which populations are most at-risk	○	○	○	○	○
To inform resource management (e.g. hospital capacity, medical supplies, testing capacity, vaccine allocation)	○	○	○	○	○
To communicate information about COVID-19 and/or risk reduction behaviors to the public	○	○	○	○	○

Display this question:

If Q157 = During the middle or control phase of the COVID-19 pandemic (approximately late 2020 - mid 2021, when non-pharmaceutical interventions, like capacity limits on indoor gatherings, and vaccinations, were used to keep the pandemic under control)

Q159 For your role, please rate the importance of these research objectives during the middle/control phase of the COVID-19 pandemic (approximately late 2020 to mid 2021, when

non-pharmaceutical interventions, like capacity limits on indoor gatherings, and vaccinations were used to keep the pandemic under control)

	Not at all important	A little important	Somewhat important	Very important	Extremely important
To learn about the current situation and increase situational awareness	○	○	○	○	○
To predict the near future	○	○	○	○	○
To learn about factors that impact SARS-CoV-2 transmission	○	○	○	○	○
To learn about the effectiveness of vaccination	○	○	○	○	○
To learn about the effectiveness of different policy interventions and/or surveillance systems	○	○	○	○	○
To learn about possible long-term scenarios	○	○	○	○	○
To learn which populations are most at-risk	○	○	○	○	○
To inform resource management (e.g. hospital capacity, medical supplies, testing capacity, vaccine allocation)	○	○	○	○	○
To communicate information about COVID-19 and/or risk reduction behaviors to the public	○	○	○	○	○

Display this question:

If Q157 = None of the above

Q160 For your role, please rate the importance of these research objectives.

	Not at all important	A little important	Somewhat important	Very important	Extremely important
To learn about the current situation and increase situational awareness	○	○	○	○	○
To predict the near future	○	○	○	○	○
To learn about factors that impact SARS-CoV-2 transmission	○	○	○	○	○
To learn about the effectiveness of vaccination	○	○	○	○	○
To learn about the effectiveness of different policy interventions and/or surveillance systems	○	○	○	○	○
To learn about possible long-term scenarios	○	○	○	○	○
To learn which populations are most at-risk	○	○	○	○	○
To inform resource management (e.g. hospital capacity, medical supplies, testing capacity, vaccine allocation)	○	○	○	○	○
To communicate information about COVID-19 and/or risk reduction behaviors to the public	○	○	○	○	○

Q161 Were there any other research objectives that were important for your work?

○ Yes

○ No

Display this question:

If Q161 = Yes

Q162 Please list and rate the importance of any other research objectives.

	Not at all important	A little important	Somewhat important	Very important	Extremely important
Research Objective 1:	○	○	○	○	○
Research Objective 2:	○	○	○	○	○
Research Objective 3:	○	○	○	○	○
Research Objective 4:	○	○	○	○	○
Research Objective 5:	○	○	○	○	○

Using Datasets or Models – Use of Data

Q163 Did you do any of the following types of work with COVID-19 data?

- ▢ I managed data collected or aggregated within my institution
- ▢ I used data collected or managed internally to inform our COVID-19 response work
- ▢ I used external data to inform our COVID-19 response work
- ▢ Other, please specify ____

Display this question:

If Q163 = I managed data collected or aggregated within my institution

Q164 Which of the following data types did you collect or manage internally for your institution?

- ☐ COVID-19 case or death data
- ☐ COVID-19 hospitalizations data
- ☐ Other epidemiological data (e.g. influenza, SARS-CoV-1)
- ☐ Vaccines allocated or administered data
- ☐ Testing data (e.g. number of tests, positivity rate)
- ☐ Serological surveillance data
- ☐ Electronic medical records or individual-level health data
- ☐ Genomic surveillance data (e.g. variant prevalence)
- ☐ Wastewater surveillance data
- ☐ Mobility data
- ☐ Other behavioral data (e.g. survey, social media or news sentiment analysis)
- ☐ COVID-19 response policy data
- ☐ Climate or environmental data (e.g. temperature, humidity)
- ☐ Consumer data (e.g. home test, mask, and disinfectant purchases)
- ☐ Health risk factors data

☐ Other demographic data

☐ Other, please specify ____

Display this question:

If Q163 = I managed data collected or aggregated within my institution

Q165 Please rate to what extent the following challenges impacted your work managing internal COVID-19 data.

	Not impactful	Slightly impactful	Moderately impactful	Impactful	Strongly impactful
Issues with data systems (e.g. ease of use, lack of interoperability between data systems)	○	○	○	○	○
Inconsistencies in reporting (e.g. between jurisdictions or departments)	○	○	○	○	○
Data privacy concerns	○	○	○	○	○
Difficulties with data sharing or establishing data use agreements	○	○	○	○	○
Difficulties with adopting standard definitions of variables of interest	○	○	○	○	○
Political (national, local, or institutional) influences	○	○	○	○	○
Insufficient funding	○	○	○	○	○
Insufficient human resources with necessary skills	○	○	○	○	○

Display this question:

If Q163 = I used data collected or managed internally to inform our COVID-19 response work

Or Q163 = I used external data to inform our COVID-19 response work

Q166 Please select which of the following data types you used to inform COVID-19 response.

- ☐ COVID-19 case or death data
- ☐ COVID-19 hospitalizations data
- ☐ Other epidemiological data (e.g. influenza, SARS-CoV-1)
- ☐ Vaccines allocated or administered data
- ☐ Testing data (e.g. number of tests, positivity rate)
- ☐ Serological surveillance data
- ☐ Electronic medical records or individual-level health data
- ☐ Genomic surveillance data (e.g. variant prevalence)
- ☐ Wastewater surveillance data
- ☐ Mobility data
- ☐ Other behavioral data (e.g. survey, social media or news sentiment analysis)
- ☐ COVID-19 response policy data
- ☐ Climate or environmental data (e.g. temperature, humidity)
- ☐ Consumer data (e.g. home test, mask, and disinfectant purchases)
- ☐ Health risk factors data

- [ ] Other demographic data
- [ ] Other, please specify ________________
- [ ] Other, please specify ________________
- [ ] Other, please specify ________________

Display this question:

If Q163 = I used data collected or managed internally to inform our COVID-19 response work

And Q163 = I used external data to inform our COVID-19 response work

Q167 Which of the data types were accessed internally versus externally?

Only show options selected in Q166

	Internally	Externally
COVID-19 case or death data	○	○
COVID-19 hospitalizations data	○	○
Other epidemiological data (e.g. influenza, SARS-CoV-1)	○	○
Vaccines allocated or administered data	○	○
Testing data (e.g. number of tests, positivity rate)	○	○
Serological surveillance data	○	○
Electronic medical records or individual-level health data	○	○
Genomic surveillance data (e.g. variant prevalence)	○	○
Wastewater surveillance data	○	○
Mobility data	○	○
Other behavioral data (e.g. survey, social media or news sentiment analysis)	○	○
COVID-19 response policy data	○	○
Climate or environmental data (e.g. temperature, humidity)	○	○
Consumer data (e.g. home test, mask, and disinfectant purchases)	○	○
Health risk factors data	○	○
Other demographic data	○	○

Other, please specify	○	○
Other, please specify	○	○
Other, please specify	○	○

Display this question:

If Q163 = I used external data to inform our COVID-19 response work

Q168 How did you access COVID-19 data?

- ☐ The data was publicly available
- ☐ Collaboration with researchers within my institution
- ☐ Collaboration with external researchers
- ☐ Private source, paid for access
- ☐ Other private source, did not pay for access
- ☐ Other, please specify

____

Display this question:

If Q163 = I used data collected or managed internally to inform our COVID-19 response work

Or Q163 = I used external data to inform our COVID-19 response work

Q169 What was the purpose of using this COVID-19 data?

- ☐ To learn about the current situation and increase situational awareness
- ☐ To predict the near future
- ☐ To learn about factors that impact SARS-CoV-2 transmission
- ☐ To learn about the effectiveness of vaccination
- ☐ To learn about the effectiveness of different policy interventions and/or surveillance systems
- ☐ To learn about possible long-term scenarios
- ☐ To learn which populations are most at-risk
- ☐ To inform resource management (e.g. hospital capacity, medical supplies, testing capacity, vaccine allocation)
- ☐ To communicate information about COVID-19 and/or risk reduction behaviors to the public
- ☐ Other, please specify

____

Display this question:

If Q163 = I used data collected or managed internally to inform our COVID-19 response work

Or Q163 = I used external data to inform our COVID-19 response work

Q170 Did you use datasets from any of the following sources?

- ☐ JHU CSSE COVID-19 case and death data or dashboard
- ☐ HHS Hospitalization Data
- ☐ Delphi Epidata API (COVIDcast data, data about other diseases)
- ☐ The COVID Tracking Project
- ☐ Apple Mobility Trends Reports
- ☐ Talus Analytics policy/intervention data (COVID AMP)
- ☐ Oxford COVID-19 Government Response Tracker (OxCGRT)
- ☐ SafeGraph mobility data
- ☐ Covariants.org
- ☐ GISAID variant tracker
- ☐ US Census
- ☐ Real-time Assessment of Community Transmission (REACT) Studies – Imperial College London
- ☐ Cuebiq mobility data
- ☐ Policy/intervention data

- [ ] Epidemiological data (e.g. cases or deaths, including for a particular sub-group such as prisons or colleges)
- [ ] Mask-wearing survey data
- [ ] Other data from the New York Times
- [ ] Wastewater surveillance data
- [ ] Testing data
- [ ] Vaccination data
- [ ] Epidemiological data (e.g. cases, deaths)
- [ ] Seroprevalence data
- [ ] Other data from CDC
- [ ] Vaccine data
- [ ] Other data from Our World in Data
- [ ] COVID-19 Trends and Impact Survey
- [ ] Mobility data
- [ ] Other data from Facebook
- [ ] Community Mobility Reports

☐ Search Trends

☐ Other data from Google

☐ Other, please specify

____

☐ ⊗None of the above

Display this question:

If Q163 = I used data collected or managed internally to inform our COVID-19 response work

Or Q163 = I used external data to inform our COVID-19 response work

Q171 Please rate the usefulness of each data type for your COVID-19 work.
Only show options selected in Q166

	Not at all useful	Slightly useful	Moderately useful	Very useful	Extremely useful
COVID-19 case or death data	○	○	○	○	○
COVID-19 hospitalizations data	○	○	○	○	○
Other epidemiological data (e.g. influenza, SARS-CoV-1)	○	○	○	○	○
Vaccines allocated or administered data	○	○	○	○	○
Testing data (e.g. number of tests, positivity rate)	○	○	○	○	○
Serological surveillance data	○	○	○	○	○
Electronic medical records or individual-level health data	○	○	○	○	○
Genomic surveillance data (e.g. variant prevalence)	○	○	○	○	○
Wastewater surveillance data	○	○	○	○	○
Mobility data	○	○	○	○	○
Other behavioral data (e.g. survey, social media or news sentiment analysis)	○	○	○	○	○
COVID-19 response policy data	○	○	○	○	○
Climate or environmental data (e.g. temperature, humidity)	○	○	○	○	○
Consumer data (e.g. home test, mask, and disinfectant purchases)	○	○	○	○	○
Health risk factors data	○	○	○	○	○

Other demographic data	○	○	○	○	○
Other, please specify	○	○	○	○	○
Other, please specify	○	○	○	○	○
Other, please specify	○	○	○	○	○

Challenges of Using Data

Display this question:

If Q163 = I used data collected or managed internally to inform our COVID-19 response work

Or Q163 = I used external data to inform our COVID-19 response work

And

If Q166 = COVID-19 case or death data

Q172 Which of the following were significant challenges of using COVID-19 cases or deaths data?

- ☐ Reliability or accuracy of the data
- ☐ Timeliness of the data: whether the data available at a particular time provide information about the current time period or are out-of-date
- ☐ Limited data at the geographic resolution (state, county, tribal, city) of interest to me
- ☐ Limited data that focused on sub-groups of interest to me. Example sub-groups include children, nursing homes, prisons, racial or ethnic groups, etc.
- ☐ Lack of expertise to interpret or use data
- ☐ Challenges related to privacy or data use agreements
- ☐ Other, please specify ____
- ☐ None of the above

Display this question:

If Q163 = I used data collected or managed internally to inform our COVID-19 response work

Or Q163 = I used external data to inform our COVID-19 response work

And

If Q166 = COVID-19 hospitalizations data

Q173 Which of the following were significant challenges of using COVID-19 hospitalizations data?

- ▢ Reliability or accuracy of the data
- ▢ Timeliness of the data: whether the data available at a particular time provide information about the current time period or are out-of-date
- ▢ Limited data at the geographic resolution (state, county, tribal, city) of interest to me
- ▢ Limited data that focused on sub-groups of interest to me. Example sub-groups include children, nursing homes, prisons, racial or ethnic groups, etc.
- ▢ Lack of expertise to interpret or use data
- ▢ Challenges related to privacy or data use agreements
- ▢ Other, please specify ____
- ▢ ⊗None of the above

Display this question:

If Q163 = I used data collected or managed internally to inform our COVID-19 response work

Or Q163 = I used external data to inform our COVID-19 response work

And

If Q166 = Other epidemiological data (e.g. influenza, SARS-CoV-1)

Q174 Which of the following were significant challenges of using other epidemiological data (e.g. influenza, SARS-CoV-1)?

- ▢ Reliability or accuracy of the data
- ▢ Timeliness of the data: whether the data available at a particular time provide information about the current time period or are out-of-date
- ▢ Limited data at the geographic resolution (state, county, tribal, city) of interest to me
- ▢ Limited data that focused on sub-groups of interest to me. Example sub-groups include children, nursing homes, prisons, racial or ethnic groups, etc.
- ▢ Lack of expertise to interpret or use data
- ▢ Challenges related to privacy or data use agreements
- ▢ Other, please specify ____
- ▢ ⊗None of the above

Display this question:

If Q163 = I used data collected or managed internally to inform our COVID-19 response work

Or Q163 = I used external data to inform our COVID-19 response work

And

If Q166 = Vaccines allocated or administered data

Q175 Which of the following were significant challenges of using vaccines allocated or administered data?

- ▢ Reliability or accuracy of the data
- ▢ Timeliness of the data: whether the data available at a particular time provide information about the current time period or are out-of-date
- ▢ Limited data at the geographic resolution (state, county, tribal, city) of interest to me
- ▢ Limited data that focused on sub-groups of interest to me. Example sub-groups include children, nursing homes, prisons, racial or ethnic groups, etc.
- ▢ Lack of expertise to interpret or use data
- ▢ Challenges related to privacy or data use agreements
- ▢ Other, please specify ____
- ▢ ⊗None of the above

Display this question:

If Q163 = I used data collected or managed internally to inform our COVID-19 response work

Or Q163 = I used external data to inform our COVID-19 response work

And

If Q166 = Testing data (e.g. number of tests, positivity rate)

Q176 Which of the following were significant challenges of using testing data (e.g. number of tests, test positivity rate)?

- ▢ Reliability or accuracy of the data
- ▢ Timeliness of the data: whether the data available at a particular time provide information about the current time period or are out-of-date
- ▢ Limited data at the geographic resolution (state, county, tribal, city) of interest to me
- ▢ Limited data that focused on sub-groups of interest to me. Example sub-groups include children, nursing homes, prisons, racial or ethnic groups, etc.
- ▢ Lack of expertise to interpret or use data
- ▢ Challenges related to privacy or data use agreements
- ▢ Other, please specify ____
- ▢ ⊗None of the above

Display this question:

If Q163 = I used data collected or managed internally to inform our COVID-19 response work

Or Q163 = I used external data to inform our COVID-19 response work

And

If Q166 = Serological surveillance data

Q177 Which of the following were significant challenges of using serological surveillance data?

- ▢ Reliability or accuracy of the data
- ▢ Timeliness of the data: whether the data available at a particular time provide information about the current time period or are out-of-date
- ▢ Limited data at the geographic resolution (state, county, tribal, city) of interest to me
- ▢ Limited data that focused on sub-groups of interest to me. Example sub-groups include children, nursing homes, prisons, racial or ethnic groups, etc.
- ▢ Lack of expertise to interpret or use data
- ▢ Challenges related to privacy or data use agreements
- ▢ Other, please specify ____
- ▢ ⊗None of the above

Display this question:

If Q163 = I used data collected or managed internally to inform our COVID-19 response work

Or Q163 = I used external data to inform our COVID-19 response work

And

If Q166 = Electronic medical records or individual-level health data

Q178 Which of the following were significant challenges of using electronic medical records or individual-level health data?

- ▢ Reliability or accuracy of the data
- ▢ Timeliness of the data: whether the data available at a particular time provide information about the current time period or are out-of-date
- ▢ Limited data at the geographic resolution (state, county, tribal, city) of interest to me
- ▢ Limited data that focused on sub-groups of interest to me. Example sub-groups include children, nursing homes, prisons, racial or ethnic groups, etc.
- ▢ Lack of expertise to interpret or use data
- ▢ Challenges related to privacy or data use agreements
- ▢ Other, please specify ____
- ▢ ⊗None of the above

Display this question:

If Q163 = I used data collected or managed internally to inform our COVID-19 response work

Or Q163 = I used external data to inform our COVID-19 response work

And

If Q166 = Genomic surveillance data (e.g. variant prevalence)

Q179 Which of the following were significant challenges of using genomic surveillance data (e.g. variant prevalence)?

☐ Reliability or accuracy of the data

☐ Timeliness of the data: whether the data available at a particular time provide information about the current time period or are out-of-date

☐ Limited data at the geographic resolution (state, county, tribal, city) of interest to me

☐ Limited data that focused on sub-groups of interest to me. Example sub-groups include children, nursing homes, prisons, racial or ethnic groups, etc.

☐ Lack of expertise to interpret or use data

☐ Challenges related to privacy or data use agreements

☐ Other, please specify

____

☐ ⊗None of the above

Display this question:

If Q163 = I used data collected or managed internally to inform our COVID-19 response work

Or Q163 = I used external data to inform our COVID-19 response work

And

If Q166 = Wastewater surveillance data

Q180 Which of the following were significant challenges of using wastewater surveillance data?

▢ Reliability or accuracy of the data

▢ Timeliness of the data: whether the data available at a particular time provide information about the current time period or are out-of-date

▢ Limited data at the geographic resolution (state, county, tribal, city) of interest to me

▢ Limited data that focused on sub-groups of interest to me. Example sub-groups include children, nursing homes, prisons, racial or ethnic groups, etc.

▢ Lack of expertise to interpret or use data

▢ Challenges related to privacy or data use agreements

▢ Other, please specify ____

▢ ⊗None of the above

Display this question:

If Q163 = I used data collected or managed internally to inform our COVID-19 response work

Or Q163 = I used external data to inform our COVID-19 response work

And

If Q166 = Mobility data

Q181 Which of the following were significant challenges of using mobility data?

- ☐ Reliability or accuracy of the data
- ☐ Timeliness of the data: whether the data available at a particular time provide information about the current time period or are out-of-date
- ☐ Limited data at the geographic resolution (state, county, tribal, city) of interest to me
- ☐ Limited data that focused on sub-groups of interest to me. Example sub-groups include children, nursing homes, prisons, racial or ethnic groups, etc.
- ☐ Lack of expertise to interpret or use data
- ☐ Challenges related to privacy or data use agreements
- ☐ Other, please specify ____
- ☐ ⊗None of the above

Display this question:

If Q163 = I used data collected or managed internally to inform our COVID-19 response work

Or Q163 = I used external data to inform our COVID-19 response work

And

If Q166 = Other behavioral data (e.g. survey, social media or news sentiment analysis)

Q182 Which of the following were significant challenges of using other behavioral data (e.g. social media, news sentiment analysis)?

- ▢ Reliability or accuracy of the data
- ▢ Timeliness of the data: whether the data available at a particular time provide information about the current time period or are out-of-date
- ▢ Limited data at the geographic resolution (state, county, tribal, city) of interest to me
- ▢ Limited data that focused on sub-groups of interest to me. Example sub-groups include children, nursing homes, prisons, racial or ethnic groups, etc.
- ▢ Lack of expertise to interpret or use data
- ▢ Challenges related to privacy or data use agreements
- ▢ Other, please specify ____
- ▢ ⊗None of the above

Display this question:

If Q163 = I used data collected or managed internally to inform our COVID-19 response work

Or Q163 = I used external data to inform our COVID-19 response work

And

If Q166 = COVID-19 response policy data

Q183 Which of the following were significant challenges of using COVID-19 response policy data?

▢ Reliability or accuracy of the data

▢ Timeliness of the data: whether the data available at a particular time provide information about the current time period or are out-of-date

▢ Limited data at the geographic resolution (state, county, tribal, city) of interest to me

▢ Limited data that focused on sub-groups of interest to me. Example sub-groups include children, nursing homes, prisons, racial or ethnic groups, etc.

▢ Lack of expertise to interpret or use data

▢ Challenges related to privacy or data use agreements

▢ Other, please specify

____

▢ ⊗None of the above

Display this question:

If Q163 = I used data collected or managed internally to inform our COVID-19 response work

Or Q163 = I used external data to inform our COVID-19 response work

And

If Q166 = Climate or environmental data (e.g. temperature, humidity)

Q184 Which of the following were significant challenges of using climate or environmental data (e.g. temperature, humidity)?

- ☐ Reliability or accuracy of the data
- ☐ Timeliness of the data: whether the data available at a particular time provide information about the current time period or are out-of-date
- ☐ Limited data at the geographic resolution (state, county, tribal, city) of interest to me
- ☐ Limited data that focused on sub-groups of interest to me. Example sub-groups include children, nursing homes, prisons, racial or ethnic groups, etc.
- ☐ Lack of expertise to interpret or use data
- ☐ Challenges related to privacy or data use agreements
- ☐ Other, please specify ____
- ☐ ⊗None of the above

Display this question:

If Q163 = I used data collected or managed internally to inform our COVID-19 response work

Or Q163 = I used external data to inform our COVID-19 response work

And

If Q166 = Consumer data (e.g. home test, mask, and disinfectant purchases)

Q185 Which of the following were significant challenges of using consumer data (e.g. at-home test, mask, or disinfectant purchases)?

▢ Reliability or accuracy of the data

▢ Timeliness of the data: whether the data available at a particular time provide information about the current time period or are out-of-date

▢ Limited data at the geographic resolution (state, county, tribal, city) of interest to me

▢ Limited data that focused on sub-groups of interest to me. Example sub-groups include children, nursing homes, prisons, racial or ethnic groups, etc.

▢ Lack of expertise to interpret or use data

▢ Challenges related to privacy or data use agreements

▢ Other, please specify
____

▢ ⊗None of the above

Display this question:

If Q163 = I used data collected or managed internally to inform our COVID-19 response work

Or Q163 = I used external data to inform our COVID-19 response work

And

If Q166 = Health risk factors data

Q186 Which of the following were significant challenges of using health risk factors data?

- ▢ Reliability or accuracy of the data
- ▢ Timeliness of the data: whether the data available at a particular time provide information about the current time period or are out-of-date
- ▢ Limited data at the geographic resolution (state, county, tribal, city) of interest to me
- ▢ Limited data that focused on sub-groups of interest to me. Example sub-groups include children, nursing homes, prisons, racial or ethnic groups, etc.
- ▢ Lack of expertise to interpret or use data
- ▢ Challenges related to privacy or data use agreements
- ▢ Other, please specify ____
- ▢ ⊗None of the above

Display this question:

If Q163 = I used data collected or managed internally to inform our COVID-19 response work

Or Q163 = I used external data to inform our COVID-19 response work

And

If Q166 = Other demographic data

Q187 Which of the following were significant challenges of using other demographic data?

- Reliability or accuracy of the data
- Timeliness of the data: whether the data available at a particular time provide information about the current time period or are out-of-date
- Limited data at the geographic resolution (state, county, tribal, city) of interest to me
- Limited data that focused on sub-groups of interest to me. Example sub-groups include children, nursing homes, prisons, racial or ethnic groups, etc.
- Lack of expertise to interpret or use data
- Challenges related to privacy or data use agreements
- Other, please specify ____
- ⊗None of the above

Display this question:

If Q163 = I used data collected or managed internally to inform our COVID-19 response work

Or Q163 = I used external data to inform our COVID-19 response work

And

If Q166 = Other, please specify

Q188 Which of the following were significant challenges of using other data: ${other data entry 1 entered in Q166}?

- ☐ Reliability or accuracy of the data
- ☐ Timeliness of the data: whether the data available at a particular time provide information about the current time period or are out-of-date
- ☐ Limited data at the geographic resolution (state, county, tribal, city) of interest to me
- ☐ Limited data that focused on sub-groups of interest to me. Example sub-groups include children, nursing homes, prisons, racial or ethnic groups, etc.
- ☐ Lack of expertise to interpret or use data
- ☐ Challenges related to privacy or data use agreements
- ☐ Other, please specify ____
- ☐ ⊗None of the above

Display this question:

If Q163 = I used data collected or managed internally to inform our COVID-19 response work

Or Q163 = I used external data to inform our COVID-19 response work

And

If Q166 = Other, please specify

Q189 Which of the following were significant challenges of using other data: ${ other data entry 2 entered in Q166}?

- ☐ Reliability or accuracy of the data
- ☐ Timeliness of the data: whether the data available at a particular time provide information about the current time period or are out-of-date
- ☐ Limited data at the geographic resolution (state, county, tribal, city) of interest to me
- ☐ Limited data that focused on sub-groups of interest to me. Example sub-groups include children, nursing homes, prisons, racial or ethnic groups, etc.
- ☐ Lack of expertise to interpret or use data
- ☐ Challenges related to privacy or data use agreements
- ☐ Other, please specify ____
- ☐ ⊗None of the above

Display this question:

If Q163 = I used data collected or managed internally to inform our COVID-19 response work

Or Q163 = I used external data to inform our COVID-19 response work

And

If Q166 = Other, please specify

Q190 Which of the following were significant challenges of using other data: ${other data entry 3 entered in Q166}?

- ☐ Reliability or accuracy of the data
- ☐ Timeliness of the data: whether the data available at a particular time provide information about the current time period or are out-of-date
- ☐ Limited data at the geographic resolution (state, county, tribal, city) of interest to me
- ☐ Limited data that focused on sub-groups of interest to me. Example sub-groups include children, nursing homes, prisons, racial or ethnic groups, etc.
- ☐ Lack of expertise to interpret or use data
- ☐ Challenges related to privacy or data use agreements
- ☐ Other, please specify ____
- ☐ ⊗None of the above

Display this question:

If Q163 = I used data collected or managed internally to inform our COVID-19 response work

Or Q163 = I used external data to inform our COVID-19 response work

Q191 Please rate to what extent the following challenges impacted your efforts to use data.

	Not impactful	Slightly impactful	Moderately impactful	Impactful	Extremely impactful
Lack of expertise within your institution to interpret and/or use data	○	○	○	○	○
Lack of effective communication with external researchers with expertise who could help you interpret or use data	○	○	○	○	○
Competing priorities or limited resources, like overstretched personnel or time constraints	○	○	○	○	○

Data Not Used

Q192 Were there any data sources you wanted to use but were not able to?

- ☐ COVID-19 case or death data
- ☐ COVID-19 hospitalizations data
- ☐ Other epidemiological data (e.g. influenza, SARS-CoV-1)
- ☐ Vaccines allocated or administered data
- ☐ Testing data (e.g. number of tests, positivity rate)
- ☐ Serological surveillance data
- ☐ Electronic medical records or individual-level health data
- ☐ Genomic surveillance data (e.g. variant prevalence)
- ☐ Wastewater surveillance data
- ☐ Mobility data
- ☐ Other behavioral data (e.g. survey, social media or news sentiment analysis)
- ☐ COVID-19 response policy data
- ☐ Climate or environmental data (e.g. temperature, humidity)
- ☐ Consumer data (e.g. home test, mask, and disinfectant purchases)

- ☐ Health risk factors data
- ☐ Other demographic data
- ☐ Other, please specify ____________________
- ☐ Other, please specify ____________________
- ☐ Other, please specify ____________________
- ☐ ⊗None of the above

Display this question:

If Q192 = COVID-19 case or death data

Q193 Why weren't you able to use COVID-19 cases or deaths data for your response work?

- ▢ Did not know how to access this data
- ▢ Concerns about the reliability or quality of the data
- ▢ I found this data was not useful for my purposes
- ▢ Data wasn't available when I needed it
- ▢ Challenges related to privacy or data use agreements
- ▢ Not available for the geographic resolution needed
- ▢ Not available for the sub-groups needed (e.g. children, nursing homes, prisons, racial or ethnic groups, etc.)
- ▢ Lack of expertise to interpret or use data
- ▢ No time to analyze or interpret this type of data due to competing priorities or limited resources (e.g., overstretched personnel, time constraints).
- ▢ Other, please specify ____

Display this question:

If Q192 = COVID-19 hospitalizations data

Q194 Why weren't you able to use COVID-19 hospitalizations data for your response work?

- [ ] Did not know how to access this data
- [ ] Concerns about the reliability or quality of the data
- [ ] I found this data was not useful for my purposes
- [ ] Data wasn't available when I needed it
- [ ] Challenges related to privacy or data use agreements
- [ ] Not available for the geographic resolution needed
- [ ] Not available for the sub-groups needed (e.g. children, nursing homes, prisons, racial or ethnic groups, etc.)
- [ ] Lack of expertise to interpret or use data
- [ ] No time to analyze or interpret this type of data due to competing priorities or limited resources (e.g., overstretched personnel, time constraints).
- [ ] Other, please specify ____

Display this question:

If Q192 = Other epidemiological data (e.g. influenza, SARS-CoV-1)

Q195 Why weren't you able to use other epidemiological data (e.g., influenza, SARS-CoV-1) for your response work?

- ▢ Did not know how to access this data
- ▢ Concerns about the reliability or quality of the data
- ▢ I found this data was not useful for my purposes
- ▢ Data wasn't available when I needed it
- ▢ Challenges related to privacy or data use agreements
- ▢ Not available for the geographic resolution needed
- ▢ Not available for the sub-groups needed (e.g. children, nursing homes, prisons, racial or ethnic groups, etc.)
- ▢ Lack of expertise to interpret or use data
- ▢ No time to analyze or interpret this type of data due to competing priorities or limited resources (e.g., overstretched personnel, time constraints).
- ▢ Other, please specify ____

Display this question:

If Q192 = Vaccines allocated or administered data

Q196 Why weren't you able to use vaccines allocated or administered data for your response work?

- ☐ Did not know how to access this data
- ☐ Concerns about the reliability or quality of the data
- ☐ I found this data was not useful for my purposes
- ☐ Data wasn't available when I needed it
- ☐ Challenges related to privacy or data use agreements
- ☐ Not available for the geographic resolution needed
- ☐ Not available for the sub-groups needed (e.g. children, nursing homes, prisons, racial or ethnic groups, etc.)
- ☐ Lack of expertise to interpret or use data
- ☐ No time to analyze or interpret this type of data due to competing priorities or limited resources (e.g., overstretched personnel, time constraints).
- ☐ Other, please specify ____

Display this question:

If Q192 = Testing data (e.g. number of tests, positivity rate)

Q197 Why weren't you able to use testing data (e.g. number of tests, test positivity rate) for your response work?

- ▢ Did not know how to access this data
- ▢ Concerns about the reliability or quality of the data
- ▢ I found this data was not useful for my purposes
- ▢ Data wasn't available when I needed it
- ▢ Challenges related to privacy or data use agreements
- ▢ Not available for the geographic resolution needed
- ▢ Not available for the sub-groups needed (e.g. children, nursing homes, prisons, racial or ethnic groups, etc.)
- ▢ Lack of expertise to interpret or use data
- ▢ No time to analyze or interpret this type of data due to competing priorities or limited resources (e.g., overstretched personnel, time constraints).
- ▢ Other, please specify ____

Display this question:

If Q192 = Serological surveillance data

Q198 Why weren’t you able to use serological surveillance data for your response work?

- ☐ Did not know how to access this data
- ☐ Concerns about the reliability or quality of the data
- ☐ I found this data was not useful for my purposes
- ☐ Data wasn’t available when I needed it
- ☐ Challenges related to privacy or data use agreements
- ☐ Not available for the geographic resolution needed
- ☐ Not available for the sub-groups needed (e.g. children, nursing homes, prisons, racial or ethnic groups, etc.)
- ☐ Lack of expertise to interpret or use data
- ☐ No time to analyze or interpret this type of data due to competing priorities or limited resources (e.g., overstretched personnel, time constraints).
- ☐ Other, please specify

____

Display this question:

If Q192 = Electronic medical records or individual-level health data

Q199 Why weren't you able to use electronic medical records or individual-level health data for your response work?

- ☐ Did not know how to access this data
- ☐ Concerns about the reliability or quality of the data
- ☐ I found this data was not useful for my purposes
- ☐ Data wasn't available when I needed it
- ☐ Challenges related to privacy or data use agreements
- ☐ Not available for the geographic resolution needed
- ☐ Not available for the sub-groups needed (e.g. children, nursing homes, prisons, racial or ethnic groups, etc.)
- ☐ Lack of expertise to interpret or use data
- ☐ No time to analyze or interpret this type of data due to competing priorities or limited resources (e.g., overstretched personnel, time constraints).
- ☐ Other, please specify ____

Display this question:

If Q192 = Genomic surveillance data (e.g. variant prevalence)

Q200 Why weren't you able to use genomic surveillance data (e.g. variant prevalence) for your response work?

- ☐ Did not know how to access this data
- ☐ Concerns about the reliability or quality of the data
- ☐ I found this data was not useful for my purposes
- ☐ Data wasn't available when I needed it
- ☐ Challenges related to privacy or data use agreements
- ☐ Not available for the geographic resolution needed
- ☐ Not available for the sub-groups needed (e.g. children, nursing homes, prisons, racial or ethnic groups, etc.)
- ☐ Lack of expertise to interpret or use data
- ☐ No time to analyze or interpret this type of data due to competing priorities or limited resources (e.g., overstretched personnel, time constraints).
- ☐ Other, please specify

____

Display this question:

If Q192 = Wastewater surveillance data

Q201 Why weren't you able to use wastewater surveillance data for your response work?

▢ Did not know how to access this data

▢ Concerns about the reliability or quality of the data

▢ I found this data was not useful for my purposes

▢ Data wasn't available when I needed it

▢ Challenges related to privacy or data use agreements

▢ Not available for the geographic resolution needed

▢ Not available for the sub-groups needed (e.g. children, nursing homes, prisons, racial or ethnic groups, etc.)

▢ Lack of expertise to interpret or use data

▢ No time to analyze or interpret this type of data due to competing priorities or limited resources (e.g., overstretched personnel, time constraints).

▢ Other, please specify ____

Display this question:

If Q192 = Mobility data

Q202 Why weren't you able to use mobility data for your response work?

- ☐ Did not know how to access this data
- ☐ Concerns about the reliability or quality of the data
- ☐ I found this data was not useful for my purposes
- ☐ Data wasn't available when I needed it
- ☐ Challenges related to privacy or data use agreements
- ☐ Not available for the geographic resolution needed
- ☐ Not available for the sub-groups needed (e.g. children, nursing homes, prisons, racial or ethnic groups, etc.)
- ☐ Lack of expertise to interpret or use data
- ☐ No time to analyze or interpret this type of data due to competing priorities or limited resources (e.g., overstretched personnel, time constraints).
- ☐ Other, please specify ____

Display this question:

If Q192 = Other behavioral data (e.g. survey, social media or news sentiment analysis)

Q203 Why weren't you able to use other behavioral data (e.g. social media, news sentiment analysis) for your response work?

- ▢ Did not know how to access this data
- ▢ Concerns about the reliability or quality of the data
- ▢ I found this data was not useful for my purposes
- ▢ Data wasn't available when I needed it
- ▢ Challenges related to privacy or data use agreements
- ▢ Not available for the geographic resolution needed
- ▢ Not available for the sub-groups needed (e.g. children, nursing homes, prisons, racial or ethnic groups, etc.)
- ▢ Lack of expertise to interpret or use data
- ▢ No time to analyze or interpret this type of data due to competing priorities or limited resources (e.g., overstretched personnel, time constraints).
- ▢ Other, please specify ____

Display this question:

If Q192 = COVID-19 response policy data

Q204 Why weren't you able to use COVID-19 response policy data for your response work?

- ▢ Did not know how to access this data
- ▢ Concerns about the reliability or quality of the data
- ▢ I found this data was not useful for my purposes
- ▢ Data wasn't available when I needed it
- ▢ Challenges related to privacy or data use agreements
- ▢ Not available for the geographic resolution needed
- ▢ Not available for the sub-groups needed (e.g. children, nursing homes, prisons, racial or ethnic groups, etc.)
- ▢ Lack of expertise to interpret or use data
- ▢ No time to analyze or interpret this type of data due to competing priorities or limited resources (e.g., overstretched personnel, time constraints).
- ▢ Other, please specify ____

Display this question:

If Q192 = Climate or environmental data (e.g. temperature, humidity)

Q205 Why weren't you able to use climate or environmental data (e.g. temperature, humidity) for your response work?

- ☐ Did not know how to access this data
- ☐ Concerns about the reliability or quality of the data
- ☐ I found this data was not useful for my purposes
- ☐ Data wasn't available when I needed it
- ☐ Challenges related to privacy or data use agreements
- ☐ Not available for the geographic resolution needed
- ☐ Not available for the sub-groups needed (e.g. children, nursing homes, prisons, racial or ethnic groups, etc.)
- ☐ Lack of expertise to interpret or use data
- ☐ No time to analyze or interpret this type of data due to competing priorities or limited resources (e.g., overstretched personnel, time constraints).
- ☐ Other, please specify ____

Display this question:

If Q192 = Consumer data (e.g. home test, mask, and disinfectant purchases)

Q206 Why weren't you able to use consumer data (e.g. at-home test, mask, or disinfectant purchases) for your response work?

- ☐ Did not know how to access this data
- ☐ Concerns about the reliability or quality of the data
- ☐ I found this data was not useful for my purposes
- ☐ Data wasn't available when I needed it
- ☐ Challenges related to privacy or data use agreements
- ☐ Not available for the geographic resolution needed
- ☐ Not available for the sub-groups needed (e.g. children, nursing homes, prisons, racial or ethnic groups, etc.)
- ☐ Lack of expertise to interpret or use data
- ☐ No time to analyze or interpret this type of data due to competing priorities or limited resources (e.g., overstretched personnel, time constraints).
- ☐ Other, please specify ____

Display this question:

If Q192 = Health risk factors data

Q207 Why weren't you able to use health risk factors data for your response work?

- ☐ Did not know how to access this data
- ☐ Concerns about the reliability or quality of the data
- ☐ I found this data was not useful for my purposes
- ☐ Data wasn't available when I needed it
- ☐ Challenges related to privacy or data use agreements
- ☐ Not available for the geographic resolution needed
- ☐ Not available for the sub-groups needed (e.g. children, nursing homes, prisons, racial or ethnic groups, etc.)
- ☐ Lack of expertise to interpret or use data
- ☐ No time to analyze or interpret this type of data due to competing priorities or limited resources (e.g., overstretched personnel, time constraints).
- ☐ Other, please specify ____

Display this question:

If Q192 = Other demographic data

Q208 Why weren’t you able to use other demographic data for your response work?

- ☐ Did not know how to access this data
- ☐ Concerns about the reliability or quality of the data
- ☐ I found this data was not useful for my purposes
- ☐ Data wasn’t available when I needed it
- ☐ Challenges related to privacy or data use agreements
- ☐ Not available for the geographic resolution needed
- ☐ Not available for the sub-groups needed (e.g. children, nursing homes, prisons, racial or ethnic groups, etc.)
- ☐ Lack of expertise to interpret or use data
- ☐ No time to analyze or interpret this type of data due to competing priorities or limited resources (e.g., overstretched personnel, time constraints).
- ☐ Other, please specify ____

Display this question:

If Q192 = Other, please specify

Q209 Why weren't you able to use other data, ${Other data entry 1 from Q192}, for your response work?

- ☐ Did not know how to access this data
- ☐ Concerns about the reliability or quality of the data
- ☐ I found this data was not useful for my purposes
- ☐ Data wasn't available when I needed it
- ☐ Challenges related to privacy or data use agreements
- ☐ Not available for the geographic resolution needed
- ☐ Not available for the sub-groups needed (e.g. children, nursing homes, prisons, racial or ethnic groups, etc.)
- ☐ Lack of expertise to interpret or use data
- ☐ No time to analyze or interpret this type of data due to competing priorities or limited resources (e.g., overstretched personnel, time constraints).
- ☐ Other, please specify ____

Display this question:

If Q192 = Other, please specify

Q210 Why weren't you able to use other data, ${Other data entry 2 from Q192}, for your response work?

- ▢ Did not know how to access this data
- ▢ Concerns about the reliability or quality of the data
- ▢ I found this data was not useful for my purposes
- ▢ Data wasn't available when I needed it
- ▢ Challenges related to privacy or data use agreements
- ▢ Not available for the geographic resolution needed
- ▢ Not available for the sub-groups needed (e.g. children, nursing homes, prisons, racial or ethnic groups, etc.)
- ▢ Lack of expertise to interpret or use data
- ▢ No time to analyze or interpret this type of data due to competing priorities or limited resources (e.g., overstretched personnel, time constraints).
- ▢ Other, please specify ____

Display this question:

If Q192 = Other, please specify

Q211 Why weren't you able to use other data, ${Other data entry 3 from Q192}, for your response work?

- ▢ Did not know how to access this data
- ▢ Concerns about the reliability or quality of the data
- ▢ I found this data was not useful for my purposes
- ▢ Data wasn't available when I needed it
- ▢ Challenges related to privacy or data use agreements
- ▢ Not available for the geographic resolution needed
- ▢ Not available for the sub-groups needed (e.g. children, nursing homes, prisons, racial or ethnic groups, etc.)
- ▢ Lack of expertise to interpret or use data
- ▢ No time to analyze or interpret this type of data due to competing priorities or limited resources (e.g., overstretched personnel, time constraints).
- ▢ Other, please specify

____

Q212 Is there anything else you want to share about using COVID-19 data? Optional.

____

Using Datasets or Models – Use of Models

Q213 Did you use COVID-19 models?

- ○ Yes
- ○ No

Display this question:

If Q213 = No

Q214 Why didn't you use COVID-19 models?

- ☐ Did not know how to access models
- ☐ Concerns about the reliability or quality of modeling results
- ☐ Modeling results weren't available when I needed it
- ☐ Not available for the geographic resolution needed
- ☐ Not available for the sub-groups needed (e.g. children, nursing homes, prisons, racial or ethnic groups, etc.)
- ☐ No time to analyze or interpret models due to competing priorities or limited resources (e.g., overstretched personnel, time constraints).
- ☐ I found that models were not useful for my purposes
- ☐ Other, please specify ____

Display this question:

If Q213 = Yes

Q215 How did you access models?

- ☐ The model was publicly available
- ☐ Models were developed internally at my institution
- ☐ Collaboration with external researchers
- ☐ Private source, paid for access
- ☐ Other private source, did not pay for access
- ☐ Other, please specify

____

Display this question:

If Q213 = Yes

Q216 Did you access modeling results through any of the following sources?

- ☐ US Forecast Hub
- ☐ US Scenario Modeling Hub
- ☐ None of the above

Display this question:

If Q215 = Models were developed internally at my institution

Q217 Were you involved in developing internal models at your institution?

- ◯ Yes
- ◯ No

Internally Developed Models

Please answer the following questions about your internally developed model. If the answers to these questions changed over the lifespan of the project, please answer the questions based on the peak operation of the project.

Display this question:

If Q217 = Yes

Q218 Which of the following research objectives did your model address?

- ☐ To learn about the current situation and increase situational awareness
- ☐ To predict the near future
- ☐ To obtain virus and transmission characteristics (e.g. transmissibility, infection fatality ratio, generation time, asymptomatic proportion of cases)
- ☐ To learn about factors that impact SARS-CoV-2 transmission
- ☐ To learn about the effectiveness of vaccination
- ☐ To learn about the effectiveness of different policy interventions and/or surveillance systems
- ☐ To learn about possible long-term scenarios
- ☐ To learn which populations are most at-risk
- ☐ To inform resource management (e.g. hospital capacity, medical supplies, testing capacity, vaccine allocation)
- ☐ To communicate information about COVID-19 and/or risk reduction behaviors to the public
- ☐ Other, please specify ____

Display this question:

If Q217 = Yes

Q219 Which of the following types of modeling did your project include?

- ☐ Retrospective analysis: analyzes past data to better understand characteristics of SARS-CoV-2 or COVID-19 (such as drivers of COVID-19 dynamics or the impact of interventions)
- ☐ Nowcasting: focused on understanding current data, usually addressing reporting issues
- ☐ Forecasting: predicts near future outcomes without making assumptions about how the situation will change in the longer-term
- ☐ Projections: predicts longer-term outcomes, which requires making assumptions about the future (e.g. new variants, future interventions)
- ☐ Optimization: the model outputs provide recommendations for optimal decisions, like how to best allocate resources
- ☐ Other, please specify ____

Display this question:

If Q217 = Yes

Q220 What types of methodologies did you use for this model?

- ☐ Compartmental (SIR and variations)
- ☐ Statistical
- ☐ Agent-based
- ☐ Other, please specify ____

Display this question:

If Q217 = Yes

Q221 What was your model's geographic resolution?

- ☐ National
- ☐ Regional (smaller than national, larger than state)
- ☐ State
- ☐ County
- ☐ City or metropolitan area
- ☐ Smaller than county or city level (e.g. census tract)
- ☐ Other, please specify ________________

Display this question:

If Q217 = Yes

Q222 Please rate the extent to which the following challenges impacted your modeling work.

	Not impactful	Slightly impactful	Moderately impactful	Impactful	Strongly impactful
Model performance issues	○	○	○	○	○
Poor data quality	○	○	○	○	○
Poor data availability	○	○	○	○	○
Difficulty producing timely results	○	○	○	○	○
Insufficient funding	○	○	○	○	○
Insufficient human resources with necessary skills	○	○	○	○	○
Insufficient computational resources	○	○	○	○	○
Stakeholder or model user priorities changing	○	○	○	○	○
Limited feedback from stakeholders or model users	○	○	○	○	○
Difficulty translating data/modeling work to public health practice (e.g. informing decision-makers on policymaking, resource allocation, etc.)	○	○	○	○	○

Display this question:

If Q217 = Yes

Q223 Were there any other significant challenges that impacted your modeling work?

◯ Yes

◯ No

Display this question:

If Q223 = Yes

Q224 Please list and rate the impact of any other significant challenges that impacted your modeling work.

	Not impactful	Slightly impactful	Moderately impactful	Impactful	Strongly impactful
Challenge 1:	◯	◯	◯	◯	◯
Challenge 2:	◯	◯	◯	◯	◯
Challenge 3:	◯	◯	◯	◯	◯
Challenge 4:	◯	◯	◯	◯	◯
Challenge 5:	◯	◯	◯	◯	◯

Display this question:

If Q217 = Yes

Q225 How useful were internally developed models for COVID-19 response?

- ◯ Not at all useful
- ◯ A little useful
- ◯ Somewhat useful
- ◯ Very useful
- ◯ Extremely useful

Using Models

Display this question:

If Q213 = Yes

Q226 What was the purpose of using models?

▢ To learn about the current situation and increase situational awareness

▢ To predict the near future

▢ To learn about factors that impact SARS-CoV-2 transmission

▢ To learn about the effectiveness of vaccination

▢ To learn about the effectiveness of different policy interventions and/or surveillance systems

▢ To learn about possible long-term scenarios

▢ To learn which populations are most at-risk

▢ To inform resource management (e.g. hospital capacity, medical supplies, testing capacity, vaccine allocation)

▢ To communicate information about COVID-19 and/or risk reduction behaviors to the public

▢ Other, please specify ____

Display this question:

If Q213 = Yes

Q227 What types of models did you use?

- ☐ Retrospective analysis: analyzes past data to better understand characteristics of SARS-CoV-2 or COVID-19 (e.g. drivers of COVID-19 dynamics or the impact of interventions)
- ☐ Nowcasting: focused on understanding current data, usually addressing reporting issues
- ☐ Forecasting: predicts near future outcomes without making assumptions about how the situation will change in the longer-term
- ☐ Projections: predicts longer-term outcomes, which requires making assumptions about the future (like new variants, future interventions)
- ☐ Optimization: the model outputs provide recommendations for optimal decisions, like how to best allocate resources
- ☐ Other, please specify ____
- ☐ Other, please specify ____
- ☐ Other, please specify ____

Display this question:

If Q213 = Yes

Q228 Rate the usefulness of each model type for your COVID-19 work.
Only show options selected in Q227

	Not at all useful	A little useful	Somewhat useful	Very useful	Extremely useful
**Retrospective analysis:** analyzes past data to better understand characteristics of SARS-CoV-2 or COVID-19 (e.g. drivers of COVID-19 dynamics or the impact of interventions)	○	○	○	○	○
**Nowcasting:** focused on understanding current data, usually addressing reporting issues	○	○	○	○	○
**Forecasting:** predicts near future outcomes without making assumptions about how the situation will change in the longer-term	○	○	○	○	○
**Projections:** predicts longer-term outcomes, which requires making assumptions about the future (like new variants, future interventions)	○	○	○	○	○
**Optimization:** the model outputs provide recommendations for optimal decisions, like how to best allocate resources	○	○	○	○	○
Other, please specify	○	○	○	○	○
Other, please specify	○	○	○	○	○
Other, please specify	○	○	○	○	○

Display this question:

If Q227 = Nowcasting: focused on understanding current data, usually addressing reporting issues

Or Q227 = Forecasting: predicts near future outcomes without making assumptions about how the situation will change in the longer-term

Or Q227 = Projections: predicts longer-term outcomes, which requires making assumptions about the future (like new variants, future interventions)

Q229 Please rate the usefulness of the following types of model outputs for your COVID-19 work.   Examples of target variables include cases, deaths, or hospitalizations.

	Not at all useful	A little useful	Somewhat useful	Very useful	Extremely useful
Predictions of actual numbers of a target variable (without uncertainty estimates)	○	○	○	○	○
Predictions of actual numbers of a target variable with uncertainty estimates, such as confidence intervals	○	○	○	○	○
Predictions of critical target event points (e.g. peak size, peak timing, time to exceed specific thresholds)	○	○	○	○	○
Categorical representations of target variables (e.g. if deaths are increasing, flat, or decreasing)	○	○	○	○	○
Translations of targets to health care or public health metrics (e.g. hospital beds needed, personal protective equipment needs)	○	○	○	○	○

Display this question:

If Q213 = Yes

Q230 Please rate to what extent the following challenges impacted your efforts to use models.

	Not impactful	Slightly impactful	Moderately impactful	Impactful	Extremely impactful
Reliability of the modeling results: the degree to which the results can be depended on to be accurate	○	○	○	○	○
Timeliness of results: whether results available at a particular time provide information about the current time period or are out-of-date	○	○	○	○	○
Limited modeling at the geographic resolution (state, county, tribal, city) of interest to me	○	○	○	○	○
Limited modeling that focused on sub-groups of interest to me. Example sub-groups include children, nursing homes, prisons, racial or ethnic groups, etc.	○	○	○	○	○
Lack of expertise within your institution to interpret or use models	○	○	○	○	○
Lack of effective communication with external modelers or other researchers with expertise who could help you interpret and/or use models	○	○	○	○	○
Competing priorities or limited resources, like overstretched personnel or time constraints	○	○	○	○	○

Display this question:

If Q213 = Yes

Q231 Were there any other significant challenges that impacted your use of models?

○ Yes

○ No

Display this question:

If Q231 = Yes

Q232 Please list and rate the impact of any other significant challenges that impacted your use of models.

	Not impactful	Slightly impactful	Moderately impactful	Impactful	Strongly impactful
Challenge 1:	○	○	○	○	○
Challenge 2:	○	○	○	○	○
Challenge 3:	○	○	○	○	○
Challenge 4:	○	○	○	○	○
Challenge 5:	○	○	○	○	○

Q233 Is there anything else you want to share about using COVID-19 models? Optional.

____

Using Datasets or Models – Collaborations with Researchers

Q234 Did you collaborate with researchers? By collaborate, we mean having meetings with researchers to discuss COVID-19, for which researchers may have presented data and/or models or gave advice.

- ○ Yes
- ○ No

Display this question:

If Q234 = No

Q235 Why didn't you collaborate with researchers?

- ☐ I didn't think researchers' work was relevant for my COVID-19 response work.
- ☐ Communicating with researchers was not part of my focus or goals.
- ☐ I did not have enough time or capacity to communicate with researchers.
- ☐ I did not know how to get in contact with researchers.
- ☐ I tried to communicate with researchers but was unsuccessful.
- ☐ Other, please specify ____

Display this question:

If Q234 = No

Q236 Please rate how impactful the following challenges were for establishing successful collaborations with researchers in general.

	Not applicable	Not impactful	Slightly impactful	Moderately impactful	Impactful	Strongly impactful
Difficulty finding and getting in contact with relevant researchers	○	○	○	○	○	○
Difficulty with data sharing or establishing data use agreements	○	○	○	○	○	○
Lack of data or information available to answer our questions	○	○	○	○	○	○
Insufficient funding	○	○	○	○	○	○
Logistical or scheduling challenges	○	○	○	○	○	○
Insufficient time with researchers for them to adequately explain their work	○	○	○	○	○	○
Limited time or capacity for collaborations with researchers due to competing priorities, overstretched human resources, etc.	○	○	○	○	○	○
Researchers struggling to adapt to our changing needs	○	○	○	○	○	○
Researchers unable to produce results fast enough for decision-making timelines	○	○	○	○	○	○
Limited opportunities to provide feedback to researchers	○	○	○	○	○	○

Difficulty translating data or modeling work to public health practice (e.g. informing decision-makers on policymaking, resource allocation, etc.)	○	○	○	○	○	○

Display this question:

If Q234 = No

Q237 Were there any other significant challenges that impacted your efforts to establish collaborations with researchers?

○ Yes

○ No

Display this question:

If Q237 = Yes

Q238 Please list and rate the impact of any other significant challenges that impacted your efforts to establish collaborations with researchers.

	Not impactful	Slightly impactful	Moderately impactful	Impactful	Strongly impactful
Challenge 1:	○	○	○	○	○
Challenge 2:	○	○	○	○	○
Challenge 3:	○	○	○	○	○
Challenge 4:	○	○	○	○	○
Challenge 5:	○	○	○	○	○

Display this question:

If Q234 = Yes

Q239 Did you collaborate with more than one group of researchers?

○ Yes

○ No

Display:

If Q239 = Yes

Please select one collaboration to report on. We recommend that you select the collaboration that was most useful for your work.

Display this question:

If Q234 = Yes

Q240 Before the COVID-19 pandemic, had you communicated or worked with these researchers before?

- ◯ No, I had not communicated with these researchers before the COVID-19 pandemic
- ◯ Yes, I had been in contact with these researchers before
- ◯ Yes, I had worked with these researchers before

Display this question:

If Q234 = Yes

Q241 Was this collaboration funded?

- ▢ Yes, by my institution
- ▢ Yes, by the researchers or their institution
- ▢ Yes, by grant or award specific to the project
- ▢ No
- ▢ I don't know

Display this question:

If Q234 = Yes

Q242 Did you provide researchers with access to data that was not publicly available?

- ◯ Yes
- ◯ No

Display this question:

If Q234 = Yes

Q243 Which of the following tools did this collaboration with researchers provide?

- ▢ Access to data that was not publicly available
- ▢ Modeling tools or expertise
- ▢ Access to expert advice or policy recommendations
- ▢ Synthesis of current COVID-19 data or information
- ▢ Other, please specify ____

Display this question:

If Q234 = Yes

Q244 How frequently did you communicate with these researchers during the peak of your collaboration?

- ○ More than 10 times
- ○ 6-10 times
- ○ 3-5 times
- ○ 1-2 times
- ○ Every 2 weeks or more often
- ○ Monthly
- ○ Every 2 months
- ○ Quarterly or less often

Display this question:

If Q216 = Modeling tools or expertise

Q245 How much influence did your needs have on the modeling tools discussed?

- ☐ Researchers developed a model from scratch to meet our needs
- ☐ Researchers customized an existing model to meet our needs
- ☐ Researchers did not adopt a model to our needs
- ☐ Other, please specify

____

Display this question:

If Q216 = Modeling tools or expertise

Q246 During which stages of model development did researchers ask for feedback?

- ☐ In the early stages of model development
- ☐ Once the model was operational
- ☐ Researchers did not ask for feedback
- ☐ Other, please specify ____

Display this question:

If Q216 = Access to data that was not publicly available

Or Q216 = Synthesis of current COVID-19 data or information

Q247 How much influence did your needs have on the data discussed?

- ☐ Researchers developed a data analysis or data collection process from scratch to meet our needs
- ☐ Researchers customized an existing data analysis or data collection process or to meet our needs
- ☐ Researchers did not adopt the data analysis or data collection process to our needs
- ☐ Other, please specify ____

Display this question:

If Q216 = Access to data that was not publicly available

Or Q216 = Synthesis of current COVID-19 data or information

Q248 During which stages of data collection or analysis did researchers ask for feedback?

☐ In the early stages of data collection or analysis

☐ Once the data or its analysis was available

☐ Researchers did not ask for feedback

☐ Other, please specify ____

Display this question:

If Q234 = Yes

Q249 Please rate to what extent the following challenges impacted your collaboration.

	Not Impactful	Slightly impactful	Moderately impactful	Impactful	Strongly impactful
Difficulty with data sharing or establishing data use agreements	○	○	○	○	○
Lack of data or information available to answer our questions	○	○	○	○	○
Insufficient funding	○	○	○	○	○
Logistical or scheduling challenges	○	○	○	○	○
Insufficient time with researchers for them to adequately explain their work	○	○	○	○	○
Limited time or capacity for collaborations with researchers due to competing priorities, overstretched human resources, etc.	○	○	○	○	○
Researchers struggling to adapt to our changing needs	○	○	○	○	○
Researchers unable to produce results fast enough for decision-making timelines	○	○	○	○	○
Limited opportunities to provide feedback to researchers	○	○	○	○	○
Difficulty translating data/modeling work to public health practice (e.g. informing decision-makers on policymaking, resource allocation, etc.)	○	○	○	○	○

Display this question:

If Q234 = Yes

Q250 Were there any other significant challenges that impacted your collaboration?

◯ Yes

◯ No

Display this question:

If Q250 = Yes

Q251 Please list and rate the impact of any other significant challenges that impacted your collaboration.

	Not impactful	Slightly impactful	Moderately impactful	Impactful	Strongly impactful
Challenge 1:	◯	◯	◯	◯	◯
Challenge 2:	◯	◯	◯	◯	◯
Challenge 3:	◯	◯	◯	◯	◯
Challenge 4:	◯	◯	◯	◯	◯
Challenge 5:	◯	◯	◯	◯	◯

Display this question:

If Q234 = Yes

Q252 To what extent do you agree or disagree with the following statements about your experiences engaging with researchers?

	Strongly Disagree	Disagree	Neither agree nor disagree	Agree	Strongly agree
Researchers communicated the uncertainty and limitations of their data or modeling results.	○	○		○	○
After communicating with researchers, I was still confused about the data or modeling results and appropriate interpretations.	○	○		○	○
I built a positive working relationship with researchers that will continue into the future.	○	○		○	○

Display this question:

If Q234 = Yes

Q253 To what extent did your engagement with researchers inform your public health work (e.g. decision-making, policy, resource allocation, situational awareness)?

- ▢ What we learned from researchers directly impacted our decision-making
- ▢ What we learned from researchers informed our situational awareness
- ▢ What we learned from researchers did not impact our public health work
- ▢ Other, please specify ____

Q254 Is there anything else you want to share about collaborations with researchers? Optional.

____

Using Datasets or Models – Concluding Questions

Information Sources

Q255 Which of the following resources did you find useful for accessing information and staying informed on COVID-19 research?

- ☐ COVID-19 digital dashboards, maps, and other visualization tools
- ☐ Papers published in peer-reviewed journals
- ☐ Papers published on preprint servers (e.g. arXiv, medRxiv, bioRxiv)
- ☐ Platforms that allowed informal peer-review of papers (e.g. Pubpeer, Outbreak science PREreview)
- ☐ Print media, including their online versions (newspapers, magazines)
- ☐ In-depth science journalism (longer than a 5-minute read, online or printed)
- ☐ Broadcast media (e.g. TV, radio)
- ☐ Podcasts
- ☐ Social media (e.g. Twitter, Instagram, YouTube)
- ☐ Other online sources (e.g. blogs)
- ☐ Other, please specify ____
- ☐ ⊗None of the above

Q256 Did you rely on information from any of the following individuals or entities for staying informed on COVID-19?

- ☐ Friends or family
- ☐ Scientists or researchers
- ☐ Health professionals (e.g. doctors, nurses)
- ☐ Celebrities or media personalities
- ☐ Community leaders or activists
- ☐ Elected officials or political leaders
- ☐ Government or public health agencies
- ☐ Academic institutions
- ☐ Health care institutions, such as hospital networks
- ☐ Other, please specify ____
- ☐ ⊗None of the above

Impact on Decision-makers

In the following questions, we want to learn more about how your work impacted downstream decision making. For PHIs, relevant decision-makers would be policymakers in your jurisdiction. For healthcare institutions, relevant decision-makers would be institutional leadership that can take action based on your work or recommendations.

Q257 Did your institution interact with relevant decision-makers?

- ☐ My team directly interacted with decision-makers
- ☐ The leadership of my institution interacted with decision-makers
- ☐ My institution did not interact with decision-makers
- ☐ Other, please specify ____
- ☐ I don’t know

Q258 Please rate the extent to which you agree with the following statements about your work’s influence on relevant decision makers.

	I don't know	Strongly disagree	Disagree	Neither agree nor disagree	Agree	Strongly Agree
Decision-makers took into account our work and/or recommendations.	○	○	○	○	○	○
Decision-makers made decisions based on data, research, and/or public health knowledge when possible.	○	○	○	○	○	○
Politics (national, local, or institutional) mainly dictated decision-making.	○	○	○	○	○	○

Q259 Is there anything else you want to share about your work's influence on relevant decision-makers? Optional.

____

Concluding Questions

Q260 Over the course of the pandemic, did your institution recommend or implement any of the following interventions or policies?

☐ Stay-at-home recommendations

☐ School closures

☐ Restrictions on businesses (e.g. closures, capacity limits, limited hours)

☐ Capacity limits on indoor or outdoor gatherings

☐ Contact tracing

☐ Efforts to make COVID-19 testing more accessible

☐ Mask mandates

☐ Vaccination mandates

☐ Education or informational campaigns (e.g. dissemination of information on how COVID-19 spreads or reasons to mask or vaccinate)

☐ Addition of surge capacity for hospitals

☐ Pausing of elective procedures

☐ Interventions to better manage limited COVID-19 supplies

☐ Other, please specify

____

☐ ⊗None of the above

Q261 To what extent do you agree or disagree with the following statement: Available data and research were sufficient to support my work as a public health practitioner throughout the COVID-19 pandemic.

- ◯ Strongly disagree
- ◯ Disagree
- ◯ Neither agree nor disagree
- ◯ Agree
- ◯ Strongly agree

Q262 Were there certain data, analyses, or information you would have wanted to access that were not available to you (or not available at the needed time)? Please explain. Optional.

____

Q263 Please select the most impactful challenges of using data and modeling work to inform COVID-19 response (maximum of 5 total selections).

- ☐ Insufficient funding
- ☐ Insufficient human resources with necessary skills
- ☐ Insufficient computational resources
- ☐ Desired data was never collected
- ☐ Data accessbility issues (e.g. not publicly available, cost prohibitive, privacy or data use agreement issues)
- ☐ Data is not timely (too much lag between when data is current and when it's made available)
- ☐ Data quality issues (e.g. anomalies, lack of standardization in data definitions, sampling biases)
- ☐ Lack of direct communication between researchers and stakeholders
- ☐ Researchers unable to produce results fast enough for decision-making timelines
- ☐ Difficulty translating data/modeling work to public health practice (e.g. informing decision-makers on policymaking, resource allocation, etc.)
- ☐ Other, please specify ____

Q264 Please select your top priority areas to invest in for informing future epidemic response (maximum of 7 total selections). Investment in data could translate to higher quality data (e.g. COVID-19 cases or deaths data), more widespread collection of this data (e.g. genomic

surveillance), or new data sources being available (e.g. data that captures COVID-19 risk reduction behaviors)

- ☐ Predictions for the near future
- ☐ Determining factors that impact transmission or severity of disease
- ☐ Determining the effectiveness of different interventions (e.g. policies, vaccinations) or surveillance systems
- ☐ Insight on potential longer-term scenarios
- ☐ Optimal management or allocation of resources
- ☐ Other, please specify ____

- ☐ Cases or deaths data
- ☐ Hospitalizations data
- ☐ Other epidemiological data (e.g. influenza, SARS-CoV-1)
- ☐ Vaccines allocated or administered
- ☐ Testing data (e.g. number of tests, test positivity rate)
- ☐ Serological surveillance
- ☐ Electronic medical records or individual-level health data
- ☐ Genomic surveillance (e.g. variant prevalence)

- [ ] Wastewater surveillance
- [ ] Mobility data
- [ ] Other behavioral data (e.g. survey, social media or news sentiment analysis)
- [ ] Policy data
- [ ] Climate or environmental data (e.g. temperature, humidity)
- [ ] Consumer data (e.g. at-home test, mask, or disinfectant purchases)
- [ ] Health risk factors data
- [ ] Other demographic data
- [ ] Other, please specify ____
- [ ] Other, please specify ____

Q265 If you have any feedback you would like to provide on the survey, please enter it here. Optional.

____

End of survey for tool users
